# Rehabilitation of the lower extremities, standing and walking function in people with spinal cord injury or disease: Guideline of the German-Speaking Medical Society for Spinal Cord Injury

**DOI:** 10.3205/000338

**Published:** 2025-06-02

**Authors:** Sophie Irrgang, Sandra Himmelhaus, Kirstin Allek, Claudio Bartholet, Ines Bersch-Porada, Armin Curt, Burkhart Huber, Daniel Kuhn, Karen Kynast, Norbert Weidner, Anke Scheel-Sailer

**Affiliations:** 1 Swiss Paraplegic Research, Nottwil, Switzerland; 2 Spinal Cord Injury Center, Zentralklinik Bad Berka, Germany; 3 Spinal Cord Injury Center, University Hospital Balgrist, Zurich, Switzerland; 4 Swiss Paraplegic Center, Nottwil, Switzerland; 5 Spinal Cord Injury Rehabilitation Center, Bad Häring, Austria; 6 Clinic for Physical and Rehabilitative Medicine, Berufsgenossenschaftliche Kliniken Bergmannstrost, Halle, Germany; 7 Spinal Cord Injury Center, Heidelberg University Hospital, Heidelberg, Germany; 8 Centre for Rehabilitation and Sport Medicine, Insel Group, University Bern, Switzerland

**Keywords:** spinal cord injury, rehabilitation, lower extremities, standing, walking

## Abstract

**Introduction:**

According of the level and severity of the spinal cord injury or disease (SCI/D), and the impairment of motor, sensory, and autonomic functions, individuals with SCI/D recover some standing and walking capabilities. To increase quality of rehabilitation and use newest evidence, the clinical practice guideline (CPG) “S2e-Guideline Rehabilitation of lower extremities, standing and walking function in people with SCI/D” of the German speaking Medical Association for Paraplegiology (DMGP) was updated.

**Methods:**

Following a multi-tiered approach systematic searches were conducted to identify appropriate literature. For this purpose, the Databases PubMed, EMBASE, Cochrane Library and PEDro were searched. Recommendations on assessments were grouped to the categories “activity and participation” or “body functions/body structures”. Recommendations on interventions were labeled with outcomes standing, walking, strength, range of motion, pain and muscle tonus.

**Results:**

In total, 9,871 studies were identified during the search. Of these, four systematic reviews and eleven primary studies were utilized in composing the recommendations. A total of 25 recommendations were made, with 20 derived from the literature and 5 based on expert consensus. In total 14 functional assessments and 11 rehabilitation interventions became compiled. The assembled recommendations regarding assessments could be well built on published literature, while overall there is a paucity of literature proofing the evidence of specific interventions used in clinical practice. Therefore, the expertise of the international expert group and input from patient representatives were pivotal.

**Conclusion:**

The method of an evidence-based guideline was sufficient for the recommendation of functional assessments but showed the need scientific clarification in the field of clinically established interventions.

## Zusammenfassung

**Einleitung:**

Je nach Grad oder Ausmaß der Querschnittlähmung (QSL) und den motorischen, sensorischen und autonomen Dysfunktionen können Menschen mit einer QSL Steh- und Gehfähigkeiten entwickeln. Um die Qualität der Rehabilitation zu verbessern und die neuesten Erkenntnisse zu nutzen, wurde die „S2e-Leitlinie Rehabilitation der unteren Extremitäten, Steh- und Gehfunktion bei Menschen mit Querschnittlähmung“ der Deutschsprachigen Medizinischen Gesellschaft für Paraplegiologie (DMGP) aktualisiert.

**Methoden:**

Nach einem mehrstufigen Ansatz wurde eine systematische Suche durchgeführt, um geeignete Literatur zu identifizieren. Zu diesem Zweck wurden die Datenbanken PubMed, EMBASE, Cochrane Library und PEDro durchsucht. Empfehlungen zu Assessments wurden in die Kategorien „Aktivität und Partizipation“ oder „Körperfunktionen/Körperstrukturen“ eingeteilt. Empfehlungen zu Interventionen wurden mit den Ergebnissen Stehen, Gehen, Kraft, Bewegungsumfang, Schmerz und Muskeltonus gekennzeichnet.

**Ergebnisse:**

Insgesamt wurden bei der Suche 9.871 Studien ermittelt. Davon wurden vier systematische Übersichten und elf Primärstudien für die Erstellung der Empfehlungen herangezogen. Es wurden insgesamt 25 Empfehlungen ausgesprochen, von denen 20 aus der Literatur abgeleitet wurden und 5 auf einem Expertenkonsens basierten. Insgesamt wurden 14 Funktionsbewertungen und 11 Rehabilitationsmaßnahmen zusammengestellt. Die zusammengestellten Empfehlungen zu den Beurteilungen konnten sich gut auf die veröffentlichte Literatur stützen, während es insgesamt nur wenig Literatur gibt, die die Evidenz spezifischer, in der klinischen Praxis eingesetzter Interventionen belegt. Daher waren das Fachwissen der internationalen Expertengruppe und die Beiträge der Patientenvertreter von entscheidender Bedeutung.

**Schlussfolgerung:**

Die Methode einer evidenzbasierten Leitlinie war für die Assessments passend, zeigte aber den Bedarf an wissenschaftlicher Klärung im Bereich der klinisch etablierten Interventionen.

## 1 Introduction

A spinal cord injury or disease (SCI/D) can result in motor, sensory, and autonomic dysfunctions, significantly impacting the physical, psychological, and social well-being of affected individuals. The extent of these dysfunctions varies widely, from minimal impairment to complete loss of function below the lesion level. Therefore, depending on the level and severity of the spinal cord lesion, individuals with SCI/D may regain the ability to stand and walk [1], [2]. As a rare health condition, the population-adjusted incidence rate of SCI/D in Germany is 15.727 per million per year [3] and due to the complexity of impairment patterns usually rehabilitation of individuals with a SCI/D is best provided in specialized SCI centers. An evidence-based development of guidelines might help to summarize existing evidence, contextualize the evidence within the cultural framework of the national context and inform clinical practice across the continuum of care with the best recommendation for coordinating and choosing rehabilitation interventions. 

The guideline titled “Rehabilitation of the Lower Extremity, Standing, and Walking Function after Spinal Cord Injury” is a first update of the German speaking Society of Paraplegia (DMGP) in the framework of the Association of the Scientific Medical Societies in Germany (AWMF) and provides recommendations for the rehabilitation of patients with both complete and incomplete SCI/D across acute, subacute, and chronic phases. 

The recommendations address the patient’s functional capacity according to the “International Classification of Functioning, Disability and Health” (ICF) [4] and not on the level of lesion or the extent of motor impairment according to the “International Standards for Neurological Classification of Spinal Cord Injury” (ISNCSCI) [5]. Given that rehabilitation is based on the ICF concept, this guideline takes a particular interest in the assessment of functionality and incorporates it into the formulation of recommendations for rehabilitation interventions [6]. This allows for the planning of rehabilitation across the continuum of care.

Primarily, the guideline offers recommendations for adults, though certain recommendations can be adapted for children and adolescents as needed. Although children have different requirements, lesion characteristics and dynamic of recovery the chance to develop a guideline is even less, due to the gap of scientific publications related to the rare health condition [7]. Recognizing that functional changes may occur due to neurological recovery or based on adaptation/compensation [8], the rehabilitation process, including the selection of appropriate assessments and interventions, remains dynamic [9]. The quality of rehabilitation is enhanced through a tailored selection of interventions based on the patient’s needs, common goal formulations [10], [11], and considerations of restoration, neuroplasticity, neuromodulation, and neuroregeneration [12], [13]. Additionally, interventions may be chosen to address or prevent complications in secondary prevention [14].

Although a guideline will provide recommendations for assessments and interventions, the adherence to specific recommendations will depend on the responsible physicians and therapists, taking into account the patient’s condition, existing circumstances, and available resources [15], [16]. Because the rehabilitation of the lower extremity follows comparable treatment principles the guidelines are applicable across various care settings, including outpatient care, day-care, inpatient care, rehabilitation, specialized care, and lifelong aftercare for individuals with SCI/D [17]. The recommendations set forth in the guideline are intended for implementation by medical specialists, physiotherapists, and occupational therapists.

The aim of this guideline is to recommend assessments to measure standing or walking function and interventions to maintain or optimize standing or walking function in individuals with spinal cord injury or disease across the continuum of care. 

## 2 Methods

The methodology applied for the guideline preparation was aligned in the framework of the “AWMF”. The literature search was conducted systematically, similar to the approach used in systematic reviews [15]. The initial methodological analysis of the topic was carried out by two research assistants with therapeutic expertise. This systematic literature search ensured that the guideline was classified as an evidence-based guideline (Se2 guideline). Subsequent to the literature selection, the content was reviewed and refined by a multidisciplinary expert group. The precise timetable is outlined in Table 1 (Tab. 1). 

The expert group comprised nine professionals, including physiotherapists, occupational therapists, neurologists, orthopedic surgeons and rehabilitation physicians, delegated from the DMGP. 

### 2.1 Literature search

On March 29, 2023, the databases PubMed, EMBASE, Cochrane Library, and PEDro were searched for systematic reviews published since the completion of Version 1 (2015–2023). The basis for this search were the a priori defined key and research questions (Table 2 (Tab. 2)).

Search terms included text words and subject headings (MeSH). The following MeSH terms were used: “spinal cord injur*”, “paraplegia”, “assistive devices”, “therapeutic interventions”, “assessment”, “muscle strength”, and “walk*”. The search was complemented by screening references of the literature found and the screening of grey literature.

Due to a lack of literature found during the search for systematic reviews, 16 additional searches for primary literature were conducted in conjunction with the initial search for systematic reviews (Table 3 (Tab. 3)). 

These searches targeted studies on specific topics related to interventions or assessments. The basic text words and MeSH terms remained consistent across these searches, with additional terms incorporated to refine and specify the topics of interest. 

The comprehensive search strategy for systematic reviews and the searches for primary studies can be found in the Appendix 1 in Attachment 1.

### 2.2 Eligibility criteria

The eligibility criteria for this literature search were defined according to the PICO-framework [18] and are presented in Table 4 (Tab. 4).

For all searches, the inclusion criteria were defined as: adults (≥18 years) diagnosed with SCI/D, human studies, assessments to quantify the standing and walking function, functioning assessments, as well as validity, reliability and objectivity, therapeutic interventions to maintain or improve standing and walking function, outcomes on body functioning (strength, mobility, endurance, balance, fine and gross motors skills). Reasons for exclusion were children <18 years), other diseases, animal studies, surgical or pharmacological interventions, biochemical outcomes, other study designs. During the additional searches, these inclusion and exclusion criteria were partially specified and adapted to the specific intervention or assessment.

### 2.3 Selection process

The selection of retrieved studies was based on predefined inclusion criteria. The tool Rayyan was employed to streamline the selection process [19]. Two trained and blinded researchers (SI, SH) independently screened the studies for matching titles, abstracts, and full texts. In cases of ambiguity, an expert (ASS) was consulted to make the final decision on study inclusion or exclusion. 

### 2.4 Analysis of study quality

The quality of the retrieved studies was assessed using the checklists required by the AWMF [15]. The quality of the available literature was evaluated using different tools. 

To assess the quality of systematic reviews, the MeaSurement Tool to Assess systematic Reviews 2 (AMSTAR 2) tool was used [20]. 

To evaluate case-control studies and cross-sectional studies, the Newcastle Ottawa Quality Assessment Scale for Case Control Studies (NOS) was used [21]. 

Randomized controlled trials (RCTs) were assayed using the Cochrane risk-of-bias tool for randomized controlled trials (RoB 2) tool [22]. 

Reliability studies were reviewed using the COSMIN Risk of Bias Tool [23]. 

To finally determine the level of evidence for each included literature, the Oxford Levels of Evidence 2011 were used [24]. Classification is based on a scale of 1 to 5, with the best possible evidence level being represented by a 1.

These evaluations were conducted independently by two blinded reviewers (SI, SH) and subsequently compared. Any discrepancies were resolved through discussion with an expert (ASS). 

### 2.5 Data collection and synthesis of results

An evidence table was created for each PICO question, detailing assessments and interventions (Appendix 2 in Attachment 1). These tables contain information on the references of the included studies, the PICO elements, the main results, and the critical evaluation of the evidence. The assessments were classified into two categories to facilitate a more precise subdivision. The initial category encompassed assessments pertaining to “activity and participation,” whereas the subsequent category encompassed assessments pertaining to “body functions/body structures.” This categorization is based on the classification system outlined in the ICF model. 

To address the third research question, outcomes recorded in the studies were systematically categorized and labeled according to the underlying research question. Outcome labels were assigned to the corresponding recommendation boxes for the interventions, indicating which effects have been explicitly scientifically investigated and validated. 

### 2.6 Formulation of recommendations

Based on the comprehensive literature review, a collaborative exchange was conducted among all participating experts to develop the recommendations. The precise wording and the levels of recommendation were assigned accordingly. The assignment of recommendation levels considered methodologically prepared evidence, clinical experience, relevance and feasibility, consistency of study results, and their applicability to the target patient group and their preferences. Recommendations were categorized as strong (A), standard (B), or open (C). Throughout this process, the expert group systematically evaluated the benefits, side effects, and risks associated with each recommendation. 

### 2.7 Participation of patients

As no patients were involved during the development of the guideline, a focus group discussion was conducted with patient representatives following the guideline’s completion. Patient representatives from Germany and Switzerland participated. This patient group provided various suggestions for modifications to the recommendations, primarily regarding the wording and the levels of recommendation. The expert group reviewed these proposed changes and subsequently incorporated them into the guideline. 

## 3 Results

### 3.1 Study selection

The comprehensive search for systematic reviews yielded 4,374 articles. Following the screening of titles and abstracts, 285 articles were selected for full-text review, resulting in the inclusion of 49 systematic reviews in the guideline. Additional searches for primary literature produced 5,771 results, of which 59 articles underwent full-text screening. Ultimately, 22 primary studies were included in the guideline. 

**Figure 1:**
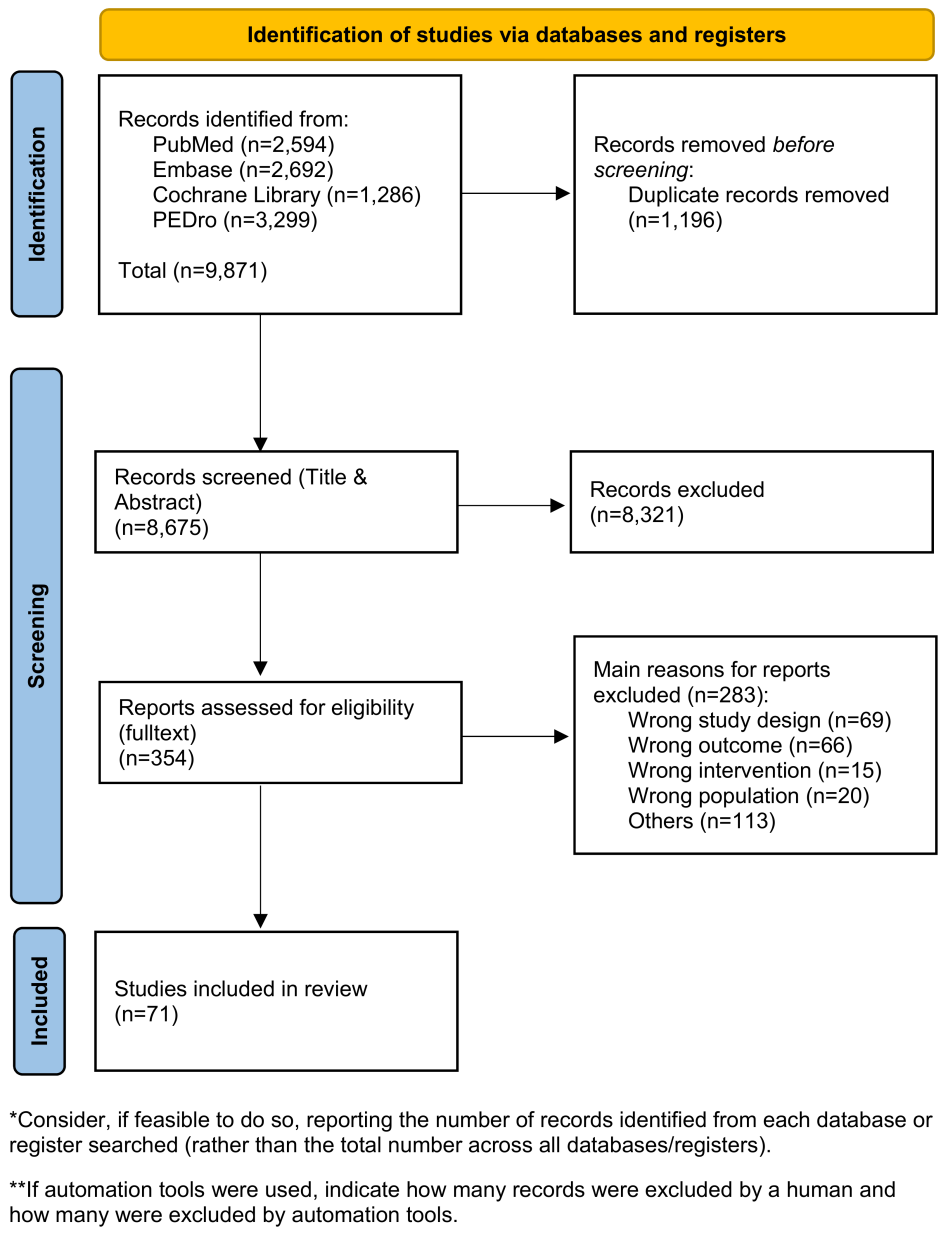
PRISMA flow diagram Adapted from Page et al. [67], licensed under CC BY 4.0 (https://creativecommons.org/licenses/by/4.0/)

After expert group revisions, four systematic reviews and eleven primary studies were utilized to address the key questions and formulate the recommendations. An overview of the selection process is presented in Figure 1 (Fig. 1), and detailed flow charts for the individual searches are available in the supplementary material (Appendix 1 in Attachment 1). A total of 25 recommendations were made, with 20 based on the literature and five derived from expert consensus. 

### 3.2 Assessments

#### Explanation of the recommendations

In this guideline, only assessments that meet psychometric quality requirements (validity, reliability) and align with the guideline’s objectives (walking and standing function) were included. The recommendations from the guideline on outcome assessment in primary treatment [25] served as the foundation for content selection. Additionally, assessments used in systematic reviews of interventions to improve walking and standing function were documented. This guideline does not aim to evaluate all assessments used in specific studies or routine clinical practice. 

In addition to the listed examinations, assessments, and measurement methods, general principles were described to underline the quality framework: a clinical examination based on medical history, inspection, and palpation is essential for individualized and specific treatment. This includes evaluating reflex status, joint examination (including stability), muscle lengths, depth sensitivity, and pain assessment [26], [27]. Joint status encompasses the range of motion (ROM) and should be performed using the neutral zero method (NNM). The manual muscle function test (MMT) should be conducted regularly, especially if there is clinical indication of muscle strength changes [28]. The MMT and joint mobility assessments are tailored to the paralysis pattern and the patient’s specific situation [29]. Muscle lengths are measured and described using established methods [30]. Leg length should be measured if clinically indicated (e.g., leg length discrepancy) for patients with SCI/D. Pain assessment should follow the recommendations of the guideline on pain in paraplegia [31]. Depth sensitivity is assessed using the tuning fork test (vibration) [26]. 

The examinations, assessments, and measurement methods are recommended for all phases: acute (up to 2 weeks after SCI/D onset); subacute (3 weeks to 6 months after SCI/D onset or initial treatment/rehabilitation); and chronic (longer than 6 months after SCI/D onset or post-discharge from initial inpatient treatment) [32]. Assessments should be consistently conducted at admission and discharge or at the start and end of outpatient, day-care, or inpatient rehabilitation, during annual reviews, or when there is clinically observed deterioration in functional capacity. Unless otherwise stated, all assessments are applicable to patients with both complete and incomplete SCI/D. 

In special cases, additional assessments may be utilized [33]. Consequently, the Spinal Cord Injury Functional Ambulation Inventory (SCI-FAI), previously given a “may” recommendation in the first version of the guideline, is no longer included. Following the overarching rehabilitation objectives, assessments for activities and participation are prioritized, followed by assessments for structure and function. 

The recommendations for the assessments are presented in Table 5 (Tab. 5) and Table 6 (Tab. 6). They are organized into two categories: recommendations pertaining to the topic of “activity and participation” and recommendations pertaining to the topic of “body functions/body structures.”

### 3.3 Interventions

#### Explanation of the recommendations

When describing interventions the following five topics evolved as relevant for the individualized adaptation of interventions:

##### Selection of interventions

The selection of interventions for individuals with SCI/D is influenced by several key aspects:

- The person with SCI/D with their biological, psychological and social circumstances
- The expected complications in the context of SCI/D
- The jointly defined goals in terms of improving functional capacity
- The organizational and structural conditions in the cross-sectional center

##### Individual with SCI/D: bio-psycho-social considerations

Interventions are tailored to the sensorimotor, neurological, and medical capabilities of the individual, addressing their functional mobility limitations, cognitive impairments, and complications such as pain and spasticity. Additionally, patient-specific factors such as exhaustion and fatigue are integrated into the individualized therapy design.

##### Anticipated complications in the context of SCI/D

The selection of treatments includes interventions aimed at preventing anticipated complications such as contractures, pain, or fractures.

##### Jointly defined goals for improving functional capacity

Goals should be collaboratively established with the patient, adhering to the SMART criteria (Specific, Measurable, Achievable, Relevant, Time-bound) [10]. The effectiveness of the intervention should be reassessed after a defined period using appropriate assessments [34]. Corresponding assessments should be conducted at the beginning and end of the therapy phase to evaluate the formulated goals or intermediate goals. Additionally, the guideline on outcome assessment in initial treatment after newly acquired spinal cord injury [25] should be referenced.

From a clinical perspective, interventions can directly or indirectly influence all of these aspects. In the table of recommendations, numbers refer to the specific aspects measured in scientific studies. Furthermore, if necessary, compensation mechanisms and the use of assistive devices are trained and adapted as needed. 

##### Rehabilitation management and organizational aspects

Interventions should be selected by therapists experienced in treating SCI/D in routine clinical practice. Based on regional/focal and/or ADL-related movement therapy, which can be hands-on or hands-off with/without the use of aids, other forms of therapy are supplemented individually or in combination for limited periods, depending on individual goals, expected neurological improvements, and predominant health issues (e.g., spasticity, pain).

In the absence of evidence for individual components within the overall intervention of initial treatment, the institution and therapy team have the responsibility and flexibility to design a goal-oriented and individualized treatment plan.

Even when evidence suggests that increased intensity of active therapies, especially during the acute and subacute phases, associated with better recovery outcomes, lower limb rehabilitation must be integrated into the overall rehabilitation plan, considering other goals and therapeutic interventions, which may require compromises regarding ideal intensity.

Due to the unique situations and special needs of individuals with SCI/D, therapies should be generally conducted as individual sessions. Some interventions may be conducted in a group setting when appropriate.

This scientific approach ensures that interventions are optimally designed to address the complex needs of individuals with SCI/D, enhancing their functional capacity and overall quality of life.

All recommendations for the interventions are displayed in Table 7 (Tab. 7).

### 3.4 Outcomes

To address the third key question, all outcomes were systematically categorized and assigned specific labels. The expert group then applied these eight labels to the various identified outcome parameters and matched them with the appropriate recommendations. This process elucidates the quantity and nature of the recommendations that have been subject to scientific investigation and delineates their respective contexts. The majority of recommendations pertain to the outcome category of endurance, while the remaining categories each have two to three corresponding recommendations. Detailed information on all categories and their parameters is presented in Table 8 (Tab. 8).

Furthermore, these categories can be utilized to establish objectives that the patient can attain throughout the rehabilitation process. The aforementioned categories are not self-contained; thus, they can be utilized as standalone entities or in conjunction with one another throughout the course of therapy.

## 4 Discussion

Although sufficient literature has been identified regarding the assessment of functioning outlined in the recommendations, its quality varies widely, and suffers from significant limitations. Many studies feature small sample sizes, and comparability between studies is lacking.

Regarding rehabilitation interventions, literature was not available for all those utilized in clinical settings. As a result, some recommendations are based on expert consensus. However, a balanced combination of literature-based recommendations and those founded on the expert group’s practical experience and expertise was achieved. 

The literature included in this guideline lacks specific treatment parameters such as number of repetitions or intensities, making it impossible to provide precise information in the recommendations. The available technical expertise was essential for developing recommendations applicable in everyday practice. The inclusion of patient representatives and the implementation of focus group discussions elevated the recommendation level from B to A for several recommendations for interventions. This highlights the critical importance of incorporating the patient perspective alongside professional expertise and literature-based knowledge. 

The descriptions of the functions of walking and standing ability facilitated the presentation and description of assessments and interventions in a structured manner. This structure allows for the interconnection of discrete recommendations, thereby facilitating the formulation of a systematic sequence of assessments and interventions that are pertinent to routine clinical practice. This offers a significant advantage over other guidelines [35], [36], which merely list recommendations for walking and standing function without establishing a connection between them. As previously stated, the guideline can thus increasingly reflect everyday clinical practice, in which assessments and interventions are conducted with similar frequency and the interventions are based on the assessments performed. It is therefore important to view these recommendations as a unified set rather than as individual recommendations.

The following topics have emerged as important research questions for the rehabilitation of standing and walking function in people with SCI/D:

**What interventions are used in different cross-sectional centers, e.g. in the acute and subacute phases after SCI/D?**

The grid created to categorize assessments and interventions according to the ICF classification can serve as a basis for further observational studies. The aim could be to compare interventions in specific subgroups of people with SCI/D, considering the expected outcome of rehabilitation in different cross-sectional centers, to better understand rehabilitation of standing and walking function, and to develop targeted interventions to improve rehabilitation.

**How effective are the consensus-based interventions recommended in this guideline?**

All the consensus-based recommendations in this guideline could be a focus for research projects. The overall aim is to be able to base these recommendations on a strong evidence base in the future. This approach will enable continuous improvement in the care of people with SCI/D about their standing and walking function. By integrating evidence-based knowledge, future recommendations can be more precise and effective in improving the quality of life and functional ability of people with SCI/D. 

**What are the milestones in rehabilitation after the onset of SCI/D? Could they be used to guide the planning of rehabilitation?**

Version 1 of this guideline presented a treatment pathway with milestones that had emerged from everyday clinical practice. As there is no evidence to support these milestones, this milestone plan has been dropped. Regarding the comparability of the overall intervention of initial treatment after SCI/D, it may be useful to investigate these milestones scientifically, possibly then subgroup-specific, to structure the treatment pathway and define goals. 

## 5 Conclusion

This review underscores the significance of interdisciplinary cooperation in the development of clinical guidelines. The review revealed the existence of literature and studies pertinent to the rehabilitation of SCI/D though the quality of these sources is generally limited. Additionally, the guideline highlights a notable gap in high-quality studies addressing routine interventions, necessitating the inclusion of consensus-based recommendations despite the preference for evidence-based guidelines. To increase the quality of recommendation a S3 guideline including a structured consensus process might be better for future updates of the guideline.

## Notes

### Guideline

A full version and a comprehensive report in German language was published in the guideline portal of the AWMF [37].

### Shared first authorship

Sophie Irrgang and Sandra Himmelhaus share first authorship.

### Author contributions

The entire team contributed to the development of the underlying guideline. All authors collaboratively developed and approved the key and research questions. SI and SH were responsible for designing search strategies, screening the identified literature, assessing study quality, and collecting data. In cases of ambiguity, ASS made the final decision on study inclusion or exclusion. The decision to conduct additional searches for primary literature was made collectively during expert group discussions. SI prepared the initial manuscript draft, which SH and ASS reviewed and provided feedback on. All other authors reviewed and approved the final version of the manuscript.

### Funding

Swiss Paraplegic Research, Nottwil, Switzerland, supported the development of the guideline by funding the two research assistants and providing materials and access to databases. 

### Competing interests

The authors declare that they have no competing interests.

## Supplementary Material

Supplementary material

## Figures and Tables

**Table 1:**
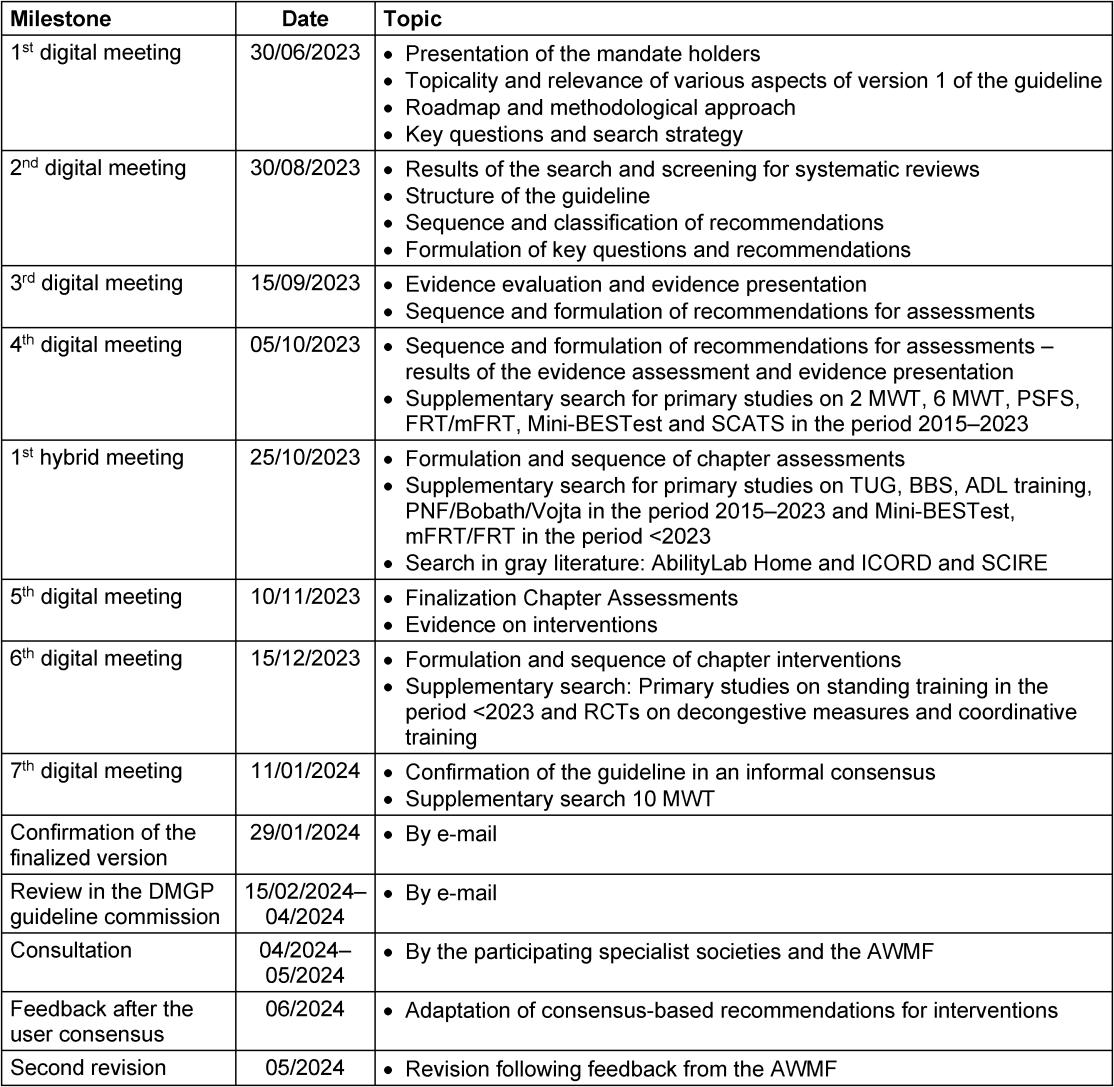
Timetable

**Table 2:**
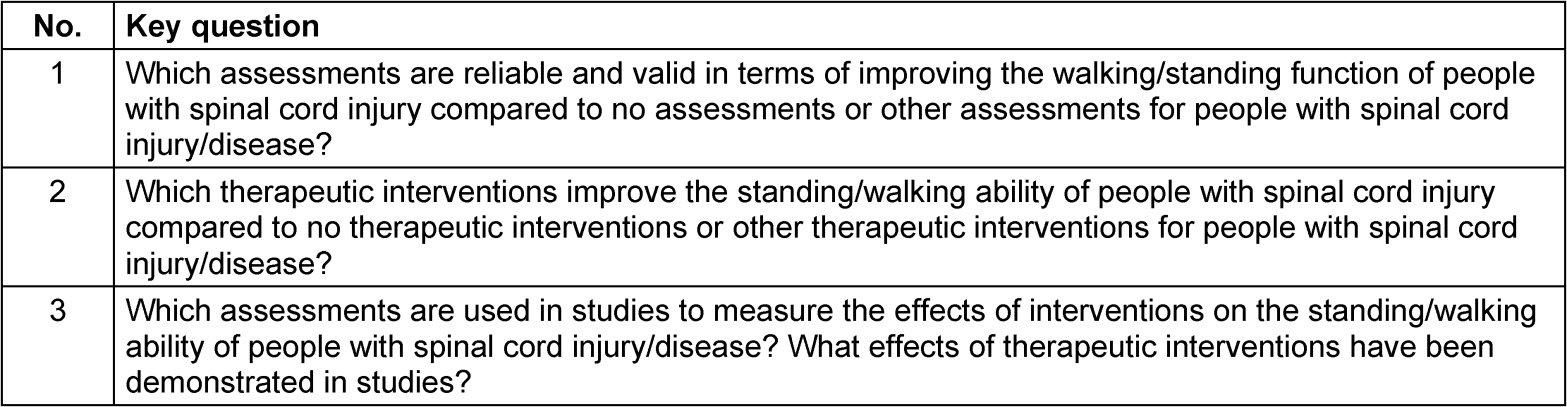
Key questions

**Table 3:**
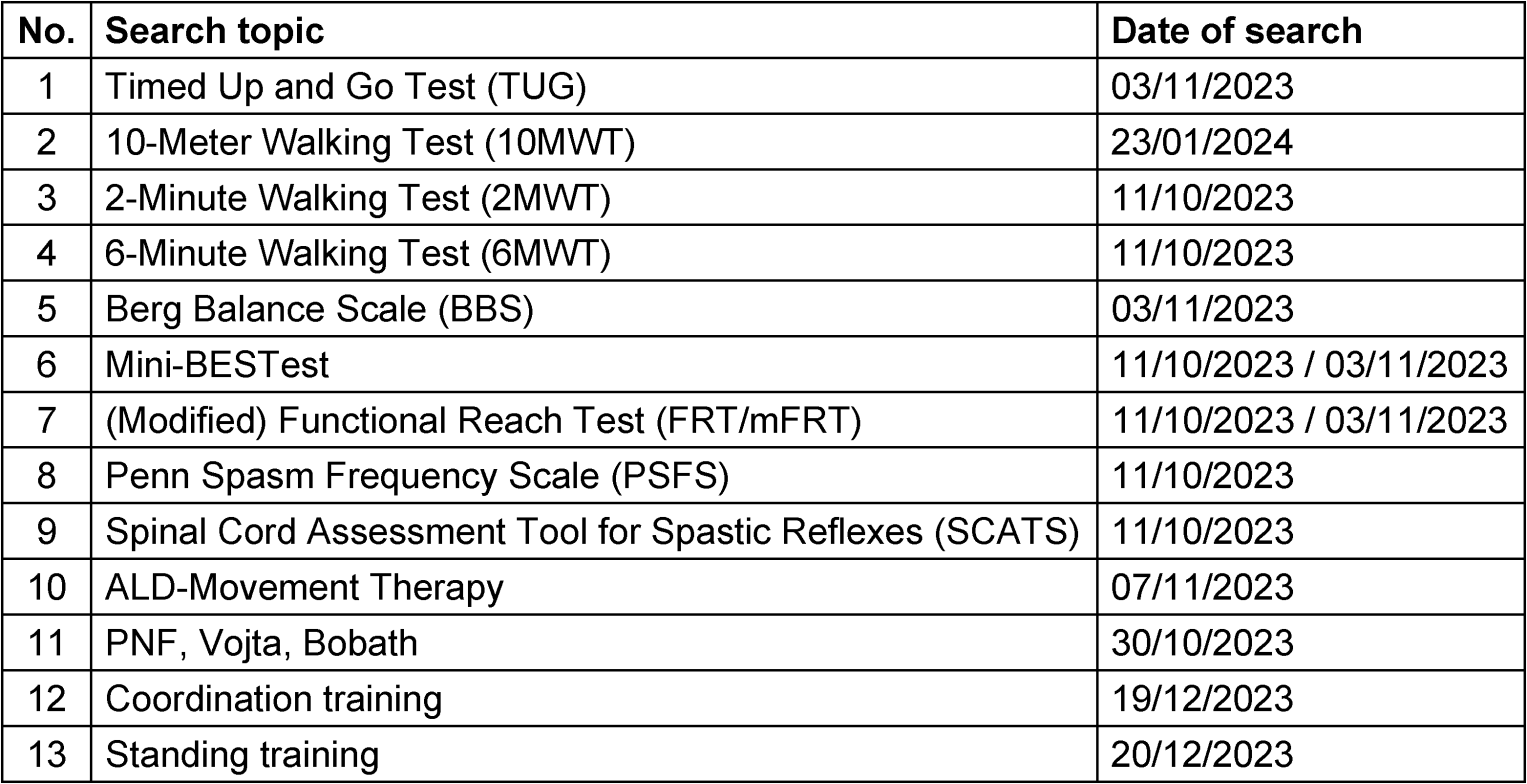
Additional searches

**Table 4:**
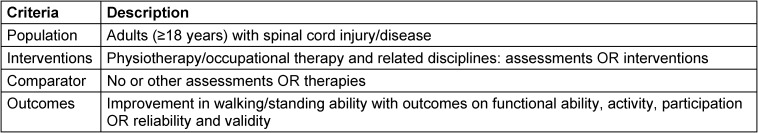
PICO-Framework

**Table 5:**
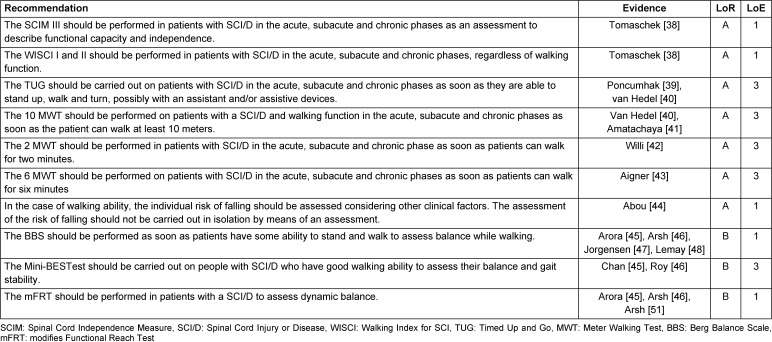
Recommendations for assessments: activity and participation

**Table 6:**
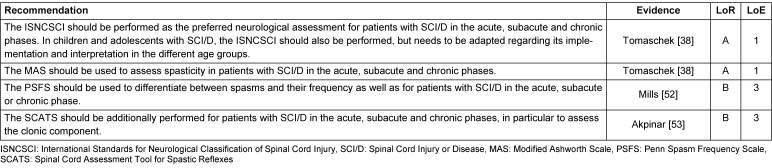
Recommendations for assessments: body functions/body structures

**Table 7:**
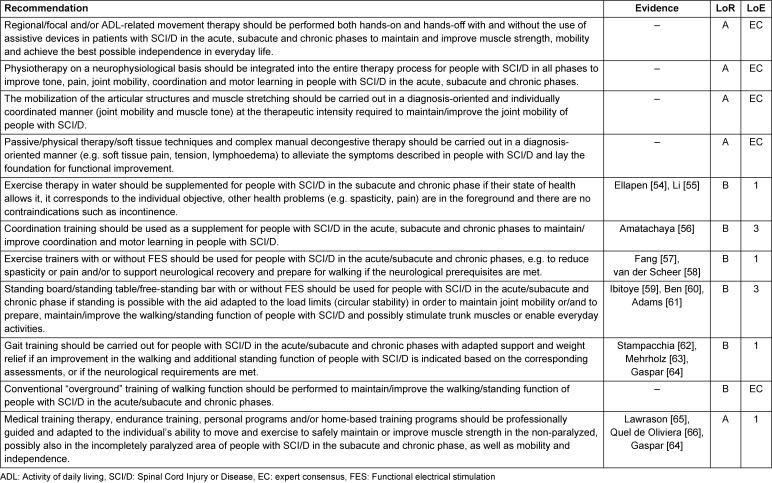
Recommendations for interventions

**Table 8:**
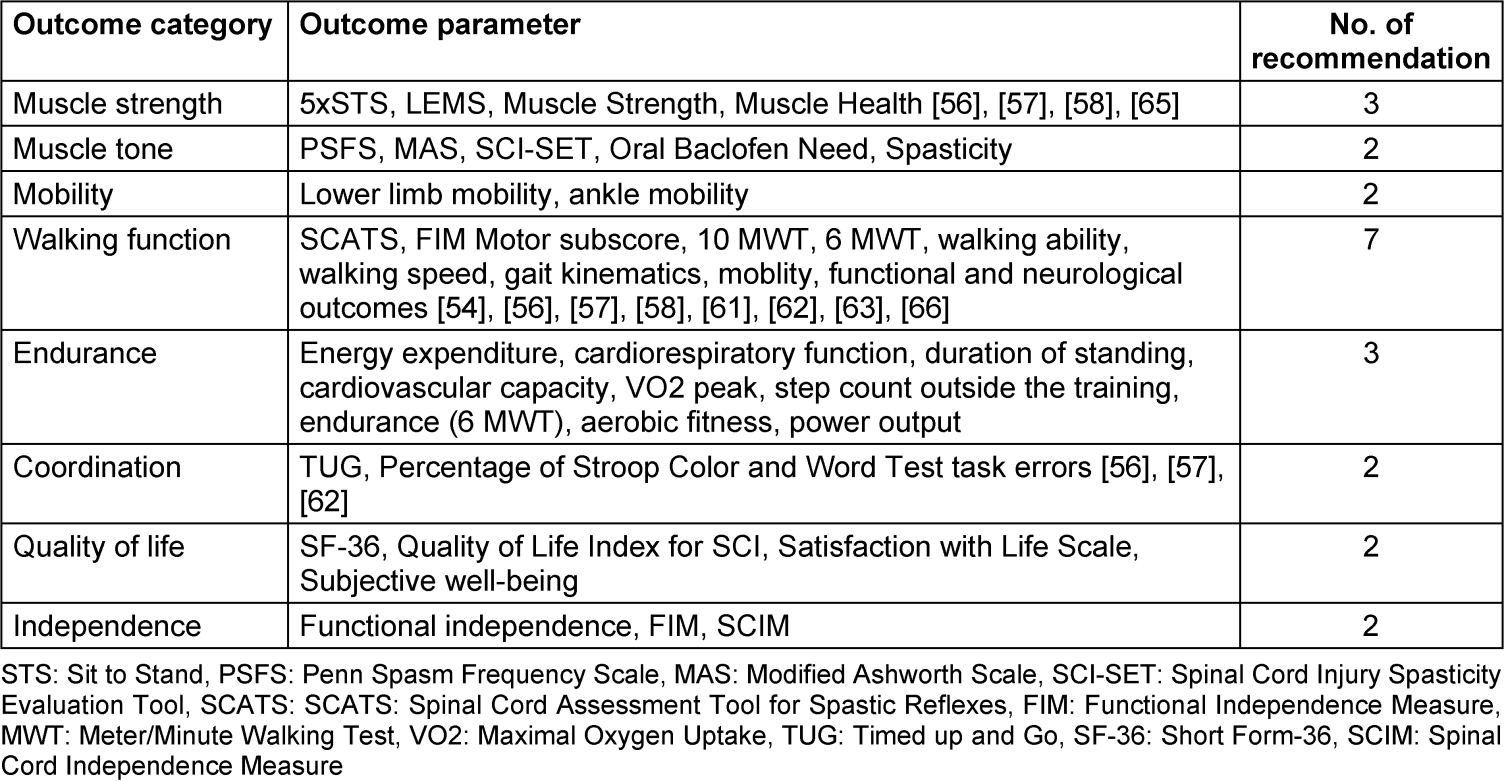
Outcome categories
