# Supplementary material for: Rehabilitation of the lower extremities, standing and walking function in people with spinal cord injury or disease: Guideline of the German-Speaking Medical Society for Spinal Cord Injury
Source: Ger Med Sci. 2025 Jun 2;23:Doc02. doi: 10.3205/000338 (PMC12247552; doi:10.3205/000338)
Supplement: Supplementary material [file GMS-23-02-s-001.pdf]

# Attachment 1: Supplementary Material

## Appendix 1: Search strategies

### A.1.1. Systematic Search for systematic reviews and meta-analyses

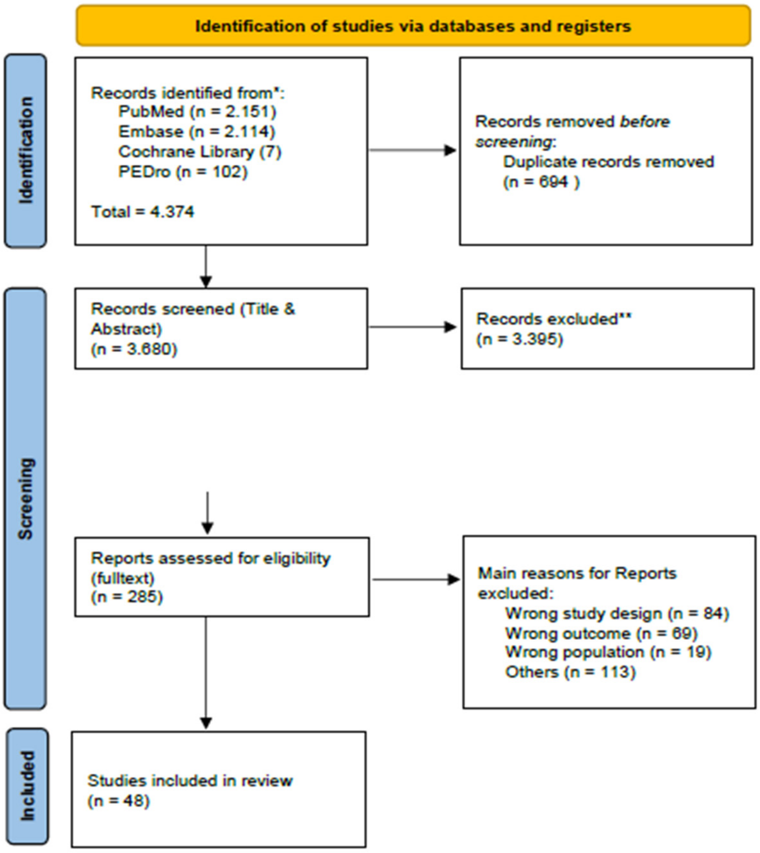


**Figure S1: Flow Chart systematic reviews and meta-analyses**

**Adapted from Page et al. [1], licensed under CC BY 4.0 (https://creativecommons.org/licenses/by/4.0/)**

Attachment to: Irrgang S, Himmelhaus S, Allek K, Bartholet C, Bersch-Porada I, Curt A, Huber B, Kuhn D, Kynast K, Weidner N, Scheel-Sailer A. Rehabilitation of the lower extremities, standing and walking function in people with spinal cord injury or disease: Guideline of the German-Speaking Medical Society for Spinal Cord Injury. GMS Ger Med Sci. 2025;23:Doc02. DOI: 10.3205/000338, URN: urn:nbn:de:0183-0003383

**Table A 1: PubMed search for systematic reviews and meta-analyses**

| # | Concept Block | Pubmed Search | Results 03/04/2023 |
|---|---|---|---|
| **1** | Spinal cord injury | "spinal cord injur*" or "parapleg*" or "paraplegia"[mesh terms] or "tetrapleg*" or "quadripleg*" or "quadriplegia"[mesh terms] or "spinal cord injuries"[mesh terms] and ((review[filter] or systematicreview[filter]) and (humans[filter]) and (english[filter] or german[filter])) | 9,650 |
| **2** | Assistive devices | "robotic" or "wheelchair*" or "wheel chair*" or "mobili*" or "early transfer" or "wheelchairs"[mesh terms] or "walk*" or "self help device*" or "self-help device*" or "equipment*" or "adaptive device*" or "tool*"or "utensil*" or "modification*" or "support system*" or "support-system*" or "adapt*" or "assistive device*" or "self-help devices"[mesh terms] or "orthosis" or "ortheses" or "orthesis" or "orthoses" or "orthotic*" or "splint*" or "splinting*" or "cast" or "casting" or “brace*” or “bracing*” or "external device*" or "orthotic devices"[mesh terms] or "assistive technolog*" or "environmental control unit*" or "joy stick*" or "joy-stick*" and ((review[filter] or systematicreview[filter]) and (humans[filter]) and (english[filter] or german[filter])) | 303,880 |
| **3** | Interventions | “strength* training” or “strength* exercise” or “biofeedback*” or “body weight support*” or “treadmill*” or “functional electrical stimulat*” or “aquatic therap*” or “manual lymphatic drainage*” or “exercise therap*” or “proprioceptive neuromuscular facilitation*” or “pnf” or “bobath*” or “vojta*” or “manual therap*” or "manual lymphatic drainage"[mesh] or "exercise therapy"[mesh] or "aquatic therapy"[mesh] or "musculoskeletal manipulations"[mesh] and ((review[filter] or systematicreview[filter]) and (humans[filter]) and (english[filter] or german[filter])) | 15,792 |
| **4** | Assessments | “assessment validation*” or “responsiveness*” or “evidence-based medicine*” or “outcome measures*” or “clinical assessment tool*” or “scales*” or “measures” or "exercise test"[mesh] or "outcome and process assessment, health care"[mesh] or "international classification of functioning, disability and health"[mesh] or "validation studies as topic"[mesh] or "prognosis"[mesh] or "classification"[mesh] or "evidence-based medicine”[mesh] or "diagnosis"[mesh] or "diagnostic tests, routine"[mesh] or "neurologic examination"[mesh] or "physical examination"[mesh] and ((review[filter] or systematicreview[filter]) and (humans[filter]) and (english[filter] or german[filter])) | 756,999 |
| **5** | Body structures | “muscle strength*” or “muscle function*” or “joint function*” or “joint mobility” or “patient mobility*” or "musculoskeletal system"[mesh] or “range of motion, articular”[mesh] or "evidence-based practice"[mesh] and ((review[filter] or systematicreview[filter]) and (humans[filter]) and (english[filter] or german[filter])) | 140,648 |
| **6** | body functions | “gait*” or “ambulation*” or “walk*” or “locomotion*” or “standing*” or “proprioception*” or “balance*” or "motor skills"[mesh] or "locomotion"[mesh] or "postural balance"[mesh] or "proprioception"[mesh] and ((review[filter] or systematicreview[filter]) and (humans[filter]) and (english[filter] or german[filter])) | 82,208 |
| **6** | Therapeutic profession | “physiotherap*” or “physical therap*” or “occupational therap*” or “exercise*” or “physical therapy modalities”[mesh] or “exercise therapy”[mesh] or "rehabilitation"[mesh] and ((review[filter] or systematicreview[filter]) and (humans[filter]) and (english[filter] or german[filter])) | 92,949 |

| | | | |
|---|---|---|---|
| **7** | Filter | 2015–2023 | |
| 8 | | (#1 and (#2 or #3 or #4 or #5 or #6)) and #7 | **2.151** |

**Table A 2: Embase search for systematic reviews and meta-analyses**

| # | Concept Block | EMBASE Search | Results 03/04/2023 |
|---|---|---|---|
| **1** | Spinal cord injury | 'spinal cord injury'/exp or 'paraplegia'/exp or 'quadriplegia'/exp or "spinal cord injur*" or "parapleg*" or "tetrapleg*" or "quadripleg*" and (2015:py or 2016:py or 2017:py or 2018:py or 2019:py or 2020:py or 2021:py or 2022:py or 2023:py) and ('human'/de or 'meta analysis'/de or 'systematic review'/de) and ([english]/lim or [german]/lim) | 45,426 |
| **2** | Assistive devices | "robotic" or "wheelchair*" or "wheel chair*" or "mobili*" or "early transfer" or "walk*" or "self help device*" or "self-help device*" or "equipment*" or "adaptive device*" or "tool*" or "utensil*" or "modification*" or "support system*" or "support-system*" or "adapt*" or "assistive device*" or "orthosis" or "ortheses" or "orthesis" or "orthoses" or "orthotic*" or "splint*" or "splinting*" or "cast" or "casting" or “brace*” or “bracing*” or "external device*" or "assistive technolog*" or "environmental control unit*" or "joy stick*" or "joy-stick*" or 'wheelchair'/exp or 'self help device'/exp or 'orthosis'/exp and ('human'/de or 'meta analysis'/de or 'systematic review'/de) and ([english]/lim or [german]/lim) | 2,602,735 |
| **3** | Interventions | “strength* training” or “strength* exercise” or “biofeedback*” or “body weight support*” or “treadmill*” or “functional electrical stimulat*” or “aquatic therap*” or “manual lymphatic drainage*” or “exercise therap*” or “proprioceptive neuromuscular facilitation*” or “pnf” or “bobath*” or “vojta*” or “manual therap*” or 'manual lymphatic drainage'/exp or 'kinesiotherapy'/exp or 'aquatic therapy'/exp or 'manipulative medicine'/exp and ('human'/de or 'meta analysis'/de or 'systematic review'/de) and ([english]/lim or [german]/lim) | 172,756 |
| **4** | Assessments | “assessment validation*” or “responsiveness*” or “evidence-based medicine*” or “outcome measures*” or “clinical assessment tool*” or “scales*” or “measures” or 'diagnostic test'/exp or 'outcome assessment'/exp or 'international classification of functioning, disability and health'/exp or 'validation study'/exp or 'clinical classification'/exp or 'evidence based medicine'/exp and ('human'/de or 'meta analysis'/de or 'systematic review'/de) and ([english]/lim or [german]/lim) | 1,542,828 |
| **5** | Body structures | “muscle strength*” or “muscle function*” or “joint function*” or “joint mobility” or “patient mobility*” or 'musculoskeletal system'/exp or 'musculoskeletal function'/exp and ('human'/de or 'meta analysis'/de or 'systematic review'/de) and ([english]/lim or [german]/lim) | 1,950,728 |
| **6** | Body functions | “gait*” or “ambulation*” or “walk*” or “locomotion*” or “standing*” or “proprioception*” or “balance*” or 'physical activity, capacity and performance'/exp or 'locomotion'/exp or 'body equilibrium'/exp and ('human'/de or 'meta analysis'/de or 'systematic review'/de) and ([english]/lim or [german]/lim) | 1,501,480 |
| **7** | Therapeutic profession | “physiotherap*” or “physical therap*” or “occupational therap*” or “exercise*” or 'rehabilitation'/exp and ('human'/de or 'meta analysis'/de or 'systematic review'/de) and ([english]/lim or [german]/lim) | 1,092,164 |
| | | gesamt | 26,668 |
| | Filter | ([cochrane review]/lim or [systematic review]/lim or [meta analysis]/lim) and ([english]/lim or [german]/lim) and [2015–2023]/py | |
| **8** | | #1 and (#2 or #3 or #4 or #5 or #6 or #7) | **2,114** |

**Table A 3: Cochrane search for systematic reviews and meta-analyses**

| # | Concept Block | Cochrane Library Search | Results 03/04/2023 |
|---|---|---|---|
| **1** | Spinal Cord Injury | "spinal cord injur*" OR "parapleg*" OR "tetrapleg*" OR "quadripleg*" | 7 |
| **2** | | MeSH descriptor: [Spinal Cord Injuries] explode all trees | 2,231 |
| **3** | | MeSH descriptor: [Paraplegia] explode all trees | 275 |
| **4** | | MeSH descriptor: [Quadriplegia] explode all trees | 231 |
| **5** | Assistive Devices | "robotic" OR "wheelchair*" OR "wheel chair*" OR "mobili*" OR "early transfer" OR "walk*" OR "self help device*" OR "self-help device*" OR "equipment*" OR "adaptive device*" OR "tool*" OR "utensil*" OR "modification*" OR "support system*" OR "support-system*" OR "adapt*" OR "assistive device*" OR "orthosis" OR "ortheses" OR "orthesis" OR "orthoses" OR "orthotic*" OR "splint*" OR "splinting*" OR "cast" OR "casting" OR “brace*” OR “bracing*” OR "external device*" OR "assistive technolog*" OR "environmental control unit*" OR "joy stick*" OR "joy-stick*" | 108,279 |
| **6** | | MeSH descriptor: [Wheelchairs] explode all trees | 246 |
| **7** | | MeSH descriptor: [Self-Help Devices] explode all trees | 510 |
| **8** | | MeSH descriptor: [Orthotic Devices] explode all trees | 2,098 |
| **9** | Interventions | “strength* training” OR “strength* exercise” OR “biofeedback*” OR “body weight support*” OR “treadmill*” OR “functional electrical stimulat*” OR “aquatic therap*” OR “manual lymphatic drainage*” OR “exercise therap*” OR “proprioceptive neuromuscular facilitation*” OR “PNF” OR “bobath*” OR “vojta*” OR “manual therap*” | 15,732 |
| **10** | | MeSH descriptor: [Manual Lymphatic Drainage] explode all trees | 34 |
| **11** | | MeSH descriptor: [Exercise Therapy] explode all trees | 19,133 |
| **12** | | MeSH descriptor: [Aquatic Therapy] explode all trees | 7 |
| **13** | | MeSH descriptor: [Musculoskeletal Manipulations] explode all trees | 3,915 |
| **14** | Assessments | “assessment*” OR "outcome measure*" OR "responsive*" | 329.919 |
| **15** | | MeSH descriptor: [Diagnostic Tests, Routine] explode all trees | 334 |
| **16** | | MeSH descriptor: [Outcome Assessment, Health Care] explode all trees | 187,950 |
| **17** | | MeSH descriptor: [International Classification of Functioning, Disability and Health] explode all trees | 23 |
| **18** | | MeSH descriptor: [Validation Study] explode all trees | 124 |
| **19** | | MeSH descriptor: [Evidence-Based Medicine] explode all trees | 2,071 |
| **20** | Body Structures | “Muscle strength*” OR “muscle function*” OR “joint function*” OR “joint mobility” OR “patient mobility*” | 24,652 |
| **21** | | MeSH descriptor: [Musculoskeletal System] explode all trees | 43,693 |
| **22** | | MeSH descriptor: [Musculoskeletal Physiological Phenomena] explode all trees | 82,858 |
| **23** | Body Functions | “gait*” OR “ambulation*” OR “walk*” OR “locomotion*” OR “standing*” OR “proprioception*” OR “balance*” | 69,296 |
| **24** | Therapeutic Profession | “physiotherap*” OR “physical therap*” OR “occupational therap*” OR “exercise*” | 124,445 |
| **25** | | MeSH descriptor: [Physical Therapy Modalities] explode all trees | 34,887 |
| **26** | | MeSH descriptor: [Occupational Therapy] explode all trees | 980 |
| **27** | Limits | with Cochrane Library publication date from Jan 2015 to Mar 2023, in Cochrane Reviews | |
| **28** | | ((#1 OR #5 OR #6 OR #7) AND ((#2 OR #8 OR #9 OR #10)) OR (#3 OR #14 OR #15 OR #16 OR #17) OR (#4 OR #19 OR #20 OR #21 OR #22 OR #23) OR (#26 OR #24 OR #25) OR #27 OR (#28 OR #12 OR #13)) AND #27 | **7** |

**Table A 4: PEDro search for systematic reviews and meta-analyses**

| PEDro Search | |
|---|---|
| Abstract & Title: Spinal cord injury | **102** |
| Method: Systematic Review | |
| Published since: 2015 | |

## A.1.2 Systematic Search for primary studies for assessments

### A.1.2.1 Timed up and go Test

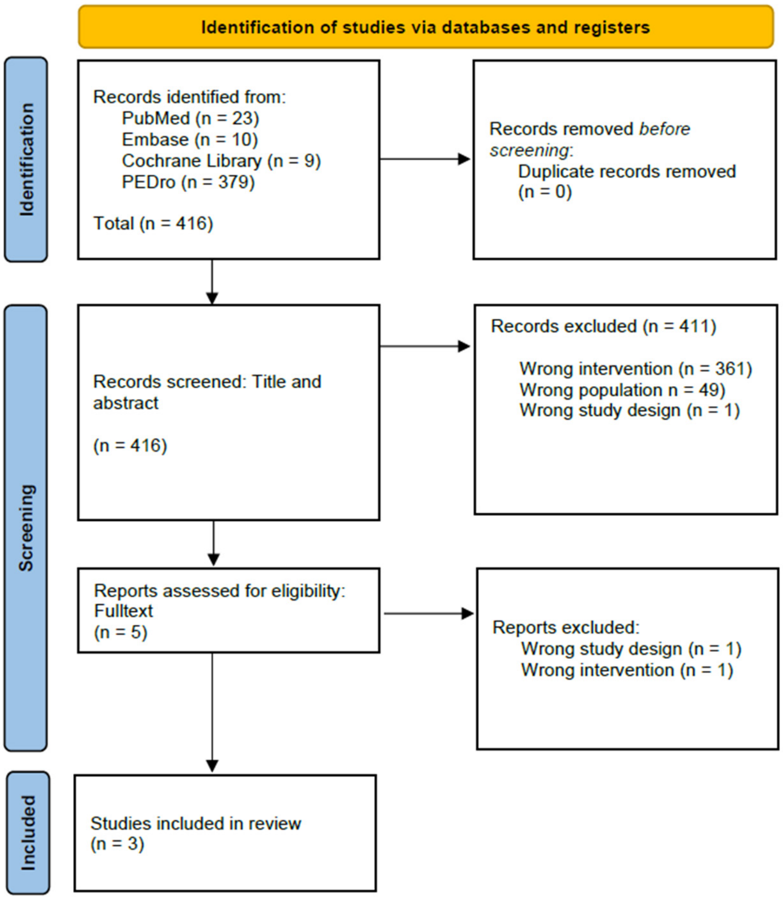


**Figure S2: Flow Chart TUG**

**Adapted from Page et al. [1], licensed under CC BY 4.0 (https://creativecommons.org/licenses/by/4.0/)**

**Table A 5: PubMed search TUG**

| # | Concept Block | PubMed Search | Results 03/11/2023 |
|---|---|---|---|
| **1** | Spinal Cord Injury (P) | "spinal cord injur*" OR "parapleg*" OR "Paraplegia"[MeSH Terms] OR "tetrapleg*" OR "quadripleg*" OR "Quadriplegia"[MeSH Terms] OR "Spinal Cord Injuries"[MeSH Terms] AND (humans[Filter]) AND (english[Filter] OR german[Filter]) | 65,830 |
| **2** | Assessments (I) | „assessment*“ OR „validation*“ OR "valid*" OR „outcome measure*”OR "Exercise Test"[Mesh] OR "Outcome and Process Assessment, Health Care"[Mesh] OR "Validation Studies as Topic"[Mesh] OR "Prognosis"[Mesh] OR "Classification"[Mesh] OR "Evidence-Based Medicine”[Mesh] OR "Neurologic Examination"[Mesh] OR "Physical Examination"[Mesh] AND (humans[Filter]) AND (english[Filter] OR german[Filter]) | 4,260,748 |
| **3** | Assessment (II) | (“timed up and go*” OR TUG) AND (humans[Filter]) AND (english[Filter] OR german[Filter]) | 6,141 |
| **4** | Study type | validation studies OR instrument validation OR external validity OR internal validity OR criterion-related validity OR concurrent validity OR discriminant validity OR content validity OR face validity OR predictive validity OR reliability OR inter-rater reliability OR intra-rater reliability OR test-retest reliability OR reproducibility OR responsiveness OR “sensitivity to change” OR evidence-based AND (humans[Filter]) AND (english[Filter] OR german[Filter]) | 2,707,555 |
| **5** | | (#1 AND #2 AND #3 AND #4) | **23** |

**Table A 6: Embase Search TUG**

| # | Concept Block | Embase Search | Results 03/11/2023 |
|---|---|---|---|
| **1** | Spinal Cord Injury | 'spinal cord injury'/exp OR 'paraplegia'/exp OR 'quadriplegia'/exp OR "spinal cord injur*" OR "parapleg*" OR "tetrapleg*" OR "quadripleg*" AND ('human'/de) AND ([english]/lim OR [german]/lim) | 110,748 |
| **2** | Assessments (I) | “assessment*” OR „validation*“ OR "valid*" OR „outcome measure*” OR 'diagnostic test'/exp OR 'outcome assessment'/exp OR 'clinical classification'/exp OR 'evidence based medicine'/exp AND ('human'/de) AND ([english]/lim OR [german]/lim) | 5,969,831 |
| **3** | Assessment (II) | “timed up and go*” OR TUG AND ('human'/de) AND ([english]/lim OR [german]/lim) | 13,242 |
| **4** | Study type | validation studies OR instrument validation OR external validity OR internal validity OR criterion-related validity OR concurrent validity OR discriminant validity OR content validity OR face validity OR predictive validity OR reliability OR inter-rater reliability OR intra-rater reliability OR test-retest reliability OR reproducibility OR responsiveness OR sensitivity to change OR evidence-based AND ('human'/de) AND ([english]/lim OR [german]/lim) | 426,582 |
| **5** | | #1 AND #2 AND #3 AND #4 | **10** |

**Table A 7: Cochrane Search TUG**

| # | Concept Block | Cochrane Library Search | Results 03/11/2023 |
|---|---|---|---|
| **1** | Spinal Cord Injury | spinal cord injur* OR parapleg* OR tetrapleg* OR quadripleg* | 4,686 |
| **2** | | MeSH descriptor: [Spinal Cord Injuries] explode all trees | 2302 |
| **3** | | MeSH descriptor: [Paraplegia] explode all trees | 293 |
| **4** | | MeSH descriptor: [Quadriplegia] explode all trees | 243 |
| **5** | Assessments (I) | assessment* OR validation* OR valid* OR outcome measure* | 567229 |
| **6** | | MeSH descriptor: [Diagnostic Techniques and Procedures] explode all trees | 310165 |
| **7** | | MeSH descriptor: [Outcome Assessment, Health Care] explode all trees | 192508 |
| **8** | | MeSH descriptor: [Evidence-Based Medicine] explode all trees | 2978 |
| **9** | Assessment (II) | "Timed up and go" OR TUG | 4958 |
| **10** | Study type | validation studies OR instrument validation OR external validity OR internal validity OR criterion-related validity OR concurrent validity OR discriminant validity OR content validity OR face validity OR predictive validity OR reliability OR inter-rater reliability OR intra-rater reliability OR test-retest reliability OR reproducibility OR responsiveness OR sensitivity to change OR evidence-based | 91898 |
| **11** | Limits | | |
| **12** | | ((#1 OR #2 OR #3 OR #4) AND (#5 OR #6 OR #7 OR #8) AND (#9 AND #10) AND #11 | **9** |

**Tabelle A 8: PEDro search TUG**

| **PEDro Search** | |
|---|---|
| Abstract & Title: Spinal cord injury | **379** |
| Method: Clinical trial | |

Attachment to: Irrgang S, Himmelhaus S, Allek K, Bartholet C, Bersch-Porada I, Curt A, Huber B, Kuhn D, Kynast K, Weidner N, Scheel-Sailer A. Rehabilitation of the lower extremities, standing and walking function in people with spinal cord injury or disease: Guideline of the German-Speaking Medical Society for Spinal Cord Injury. GMS Ger Med Sci. 2025;23:Doc02. DOI: 10.3205/000338, URN: urn:nbn:de:0183-0003383

### A.1.2.2 10-Meter Walking Test

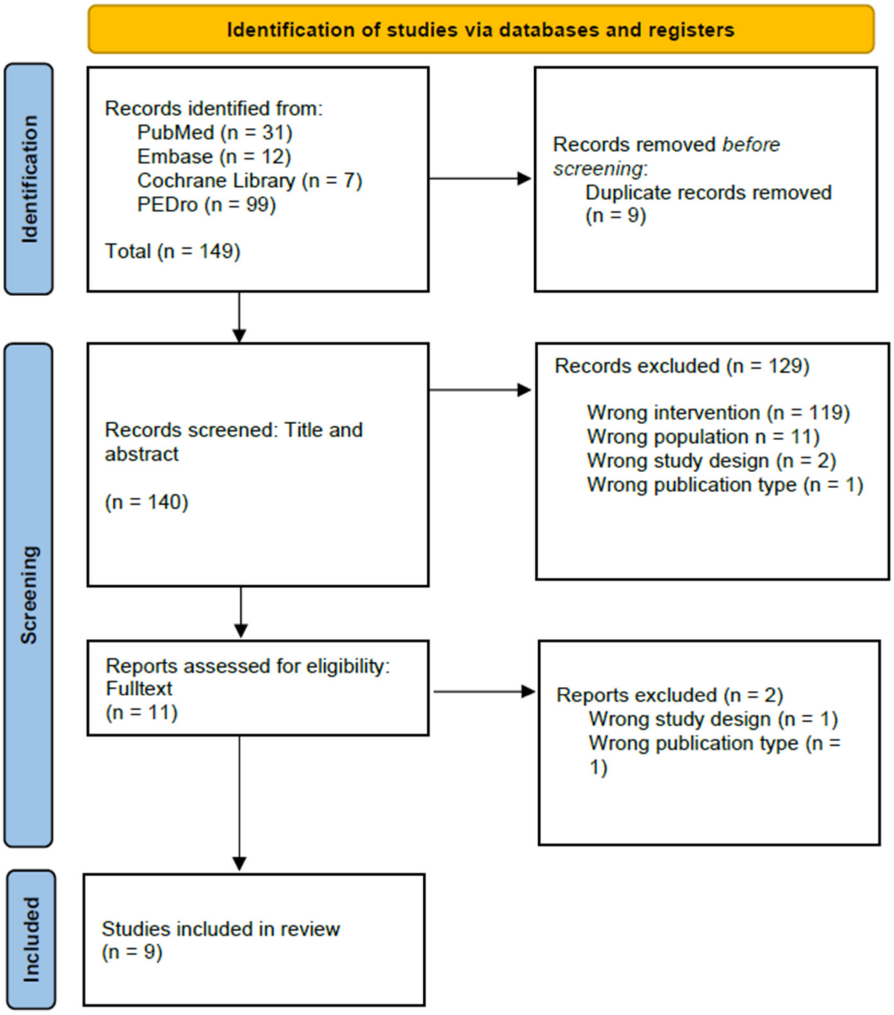


**Figure S3: Flow Chart 10MWT**

**Adapted from Page et al. [1], licensed under CC BY 4.0 (https://creativecommons.org/licenses/by/4.0/)**

**Table A 9: PubMed search 10MWT**

| # | Concept Block | PubMed Search | Results 23/01/2024 |
|---|---|---|---|
| **1** | Spinal Cord Injury (P) | ("spinal cord injur*" OR "parapleg*" OR "Paraplegia"[MeSH Terms] OR "tetrapleg*" OR "quadripleg*" OR "Quadriplegia"[MeSH Terms] OR "Spinal Cord Injuries"[MeSH Terms]) AND (humans[Filter]) AND (english[Filter] OR german[Filter]) | 66,357 |
| **2** | Assessment (II) | ("10MWT" OR "10 MWT" OR "ten meter walk test" OR "10 meter walk test" OR 'ten metre walk test' OR '10 metre walk test') AND (humans[Filter]) AND (english[Filter] OR german[Filter]) | 1,144 |
| **3** | Validity | validation studies OR instrument validation OR external validity OR internal validity OR criterion-related validity OR concurrent validity OR discriminant validity OR content validity OR face validity OR predictive validity OR reliability OR inter-rater reliability OR intra-rater reliability OR test-retest reliability OR reproducibility OR responsiveness OR sensitivity change OR evidence-based AND (humans[Filter]) AND (english[Filter] OR german[Filter]) | 3,005,406 |
| **4** | | #1 AND #2 AND #3 | **31** |

**Tabelle A 10: Embase search 10MWT**

| # | Concept Block | Embase Search | Results 23/01/2024 |
|---|---|---|---|
| **1** | Spinal Cord Injury | ('spinal cord injury'/exp OR 'paraplegia'/exp OR 'quadriplegia'/exp OR "spinal cord injur*" OR "parapleg*" OR "tetrapleg*" OR "quadripleg*") AND ('human'/de) AND ([english]/lim OR [german]/lim) | 114,885 |
| **2** | Assessment (II) | ("10MWT' OR "10 MWT" OR "ten meter walk test" OR "10 meter walk test" OR 'ten metre walk test' OR '10 metre walk test') AND ('human'/de) AND ([english]/lim OR [german]/lim) | 1,669 |
| **3** | Validation | validation studies OR instrument validation OR external validity OR internal validity OR criterion-related validity OR concurrent validity OR discriminant validity OR content validity OR face validity OR predictive validity OR reliability OR inter-rater reliability OR intra-rater reliability OR test-retest reliability OR reproducibility OR responsiveness OR sensitivity to change OR evidence-based AND ('human'/de) AND ([english]/lim OR [german]/lim) | 434,456 |
| **4** | | #1 AND #2 AND #3/#4 | **12** |

**Table A 11: Cochrane search 10MWT**

| # | Concept Block | Cochrane Library Search | Results 23.01.24 |
|---|---|---|---|
| **1** | Spinal Cord Injury | spinal cord injur* OR parapleg* OR tetrapleg* OR quadripleg* | 5362 |
| **2** | | MeSH descriptor: [Spinal Cord Injuries] explode all trees | 2317 |
| **3** | | MeSH descriptor: [Paraplegia] explode all trees | 294 |
| **4** | | MeSH descriptor: [Quadriplegia] explode all trees | 244 |
| **5** | Assessment (II) | 10MWT OR 10 MWT OR ten meter walk test OR 10 meter walk test OR ten metre walk test OR 10 metre walk test | 1606 |
| **6** | | *validation OR *validity OR validiat* OR validity* | 28666 |
| **7** | | ((#1 OR #2 OR #3 OR #4) AND #5 AND #6 | **7** |

**Table A 12: PEDro search 10MWT**

| **PEDro Search** | |
|---|---|
| Abstract & Title: Spinal cord injury | **99** |
| Method: Clinical trial | |
| Published since: <Jan 2024 | |
| Match Search terms: any (AND) | |

### A.1.2.3 2-minute Walking Test

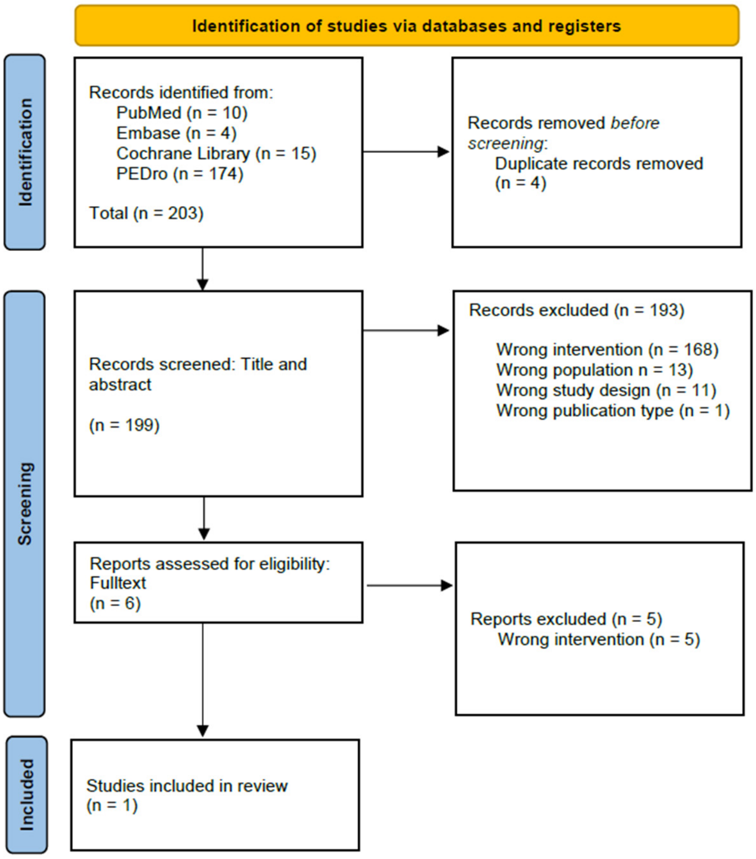


**Figure S4: Flow Chart 2MWT**

**Adapted from Page et al. [1], licensed under CC BY 4.0 (https://creativecommons.org/licenses/by/4.0/)**

Attachment to: Irrgang S, Himmelhaus S, Allek K, Bartholet C, Bersch-Porada I, Curt A, Huber B, Kuhn D, Kynast K, Weidner N, Scheel-Sailer A. Rehabilitation of the lower extremities, standing and walking function in people with spinal cord injury or disease: Guideline of the German-Speaking Medical Society for Spinal Cord Injury. GMS Ger Med Sci. 2025;23:Doc02. DOI: 10.3205/000338, URN: urn:nbn:de:0183-0003383

**Table A 13: PubMed search 2MWT**

| # | Concept Block | PubMed Search | Results 11/10/2023 |
|---|---|---|---|
| **1** | Spinal Cord Injury (P) | "spinal cord injur*" OR "parapleg*" OR "Paraplegia"[MeSH Terms] OR "tetrapleg*" OR "quadripleg*" OR "Quadriplegia"[MeSH Terms] OR "Spinal Cord Injuries"[MeSH Terms] AND (humans[Filter]) AND (english[Filter] OR german[Filter]) | 65,671 |
| **2** | Assessments (I) | „assessment*“ OR „validation*“ OR "valid*" OR „outcome measure*”OR "Exercise Test"[Mesh] OR "Outcome and Process Assessment, Health Care"[Mesh] OR "Validation Studies as Topic"[Mesh] OR "Prognosis"[Mesh] OR "Classification"[Mesh] OR "Evidence-Based Medicine”[Mesh] OR "Neurologic Examination"[Mesh] OR "Physical Examination"[Mesh] AND (humans[Filter]) AND (english[Filter] OR german[Filter]) | 4,245,199 |
| **3** | Assessment (II) | "2MWT" OR "2 MWT" OR "*walking test" OR "two minute walking test" OR "2 minute walking test" AND (humans[Filter]) AND (english[Filter] OR german[Filter]) | 4,231 |
| **4** | Study type | validation studies OR instrument validation OR external validity OR internal validity OR criterion-related validity OR concurrent validity OR discriminant validity OR content validity OR face validity OR predictive validity OR reliability OR inter-rater reliability OR intra-rater reliability OR test-retest reliability OR reproducibility OR responsiveness OR sensitivity to change OR evidence-based AND (humans[Filter]) AND (english[Filter] OR german[Filter]) | 2,804,170 |
| **5** | Filter | 2015–2023 | |
| **6** | | (#1 AND #2 AND #3 AND #4) AND #5 | **10** |

**Table A 14: Embase search 2MWT**

| # | Concept Block | Embase Search | Results 11/10/2023 |
|---|---|---|---|
| **1** | Spinal Cord Injury | 'spinal cord injury'/exp OR 'paraplegia'/exp OR 'quadriplegia'/exp OR "spinal cord injur*" OR "parapleg*" OR "tetrapleg*" OR "quadripleg*" AND ('human'/de) AND ([english]/lim OR [german]/lim) | 110,508 |
| **2** | Assessments (I) | “assessment*” OR „validation*“ OR "valid*" OR „outcome measure*” OR 'diagnostic test'/exp OR 'outcome assessment'/exp OR 'clinical classification'/exp OR 'evidence based medicine'/exp AND ('human'/de) AND ([english]/lim OR [german]/lim) | 5,944,908 |
| **3** | Assessment (II) | "2MWT" OR "2 MWT" OR "walking test" OR "two minute walking test" OR "2 minute walking test" AND ('human'/de) AND ([english]/lim OR [german]/lim) | 6,840 |
| **4** | Study type | validation studies OR instrument validation OR external validity OR internal validity OR criterion-related validity OR concurrent validity OR discriminant validity OR content validity OR face validity OR predictive validity OR reliability OR inter-rater reliability OR intra-rater reliability OR test-retest reliability OR reproducibility OR responsiveness OR sensitivity to change OR evidence-based AND ('human'/de) AND ([english]/lim OR [german]/lim) | 425,010 |
| **5** | | #1 AND #2 AND #3 AND #4 | 4 |
| **6** | Filter | 2015–2023 | **4** |

**Table A 15: Cochrane search 2MWT**

| # | Concept Block | Cochrane Library Search | Results 11/10/2023 |
|---|---|---|---|
| **1** | Spinal Cord Injury | spinal cord injur* OR parapleg* OR tetrapleg* OR quadripleg* | 5,278 |
| **2** | | MeSH descriptor: [Spinal Cord Injuries] explode all trees | 2,297 |
| **3** | | MeSH descriptor: [Paraplegia] explode all trees | 291 |
| **4** | | MeSH descriptor: [Quadriplegia] explode all trees | 243 |
| **5** | Assessments (I) | assessment* OR validation* OR valid* OR outcome measure* | 553197 |
| **6** | | MeSH descriptor: [Diagnostic Tests, Routine] explode all trees | 337 |
| **7** | | MeSH descriptor: [Outcome Assessment, Health Care] explode all trees | 192,216 |
| **8** | | MeSH descriptor: [Evidence-Based Medicine] explode all trees | 2,977 |
| **9** | Assessment (II) | 2MWT OR 2 MWT OR *walking test OR two minute walking test OR 2 minute walking test | 1,3111 |
| **10** | Study type | validation studies OR instrument validation OR external validity OR internal validity OR criterion-related validity OR concurrent validity OR discriminant validity OR content validity OR face validity OR predictive validity OR reliability OR inter-rater reliability OR intra-rater reliability OR test-retest reliability OR reproducibility OR responsiveness OR sensitivity to change OR evidence-based | 74,035 |
| **11** | Limits | with Cochrane Library publication date from Jan 2015 to Mar 2023, in Cochrane Reviews and Trials | |
| **12** | | ((#1 OR #2 OR #3 OR #4) AND (#5 OR #6 OR #7 OR #8) AND (#9 AND #10) AND #11 | **15** |

**Table A 16: PEDro search 2MWT**

| **PEDro Search** | |
|---|---|
| Abstract & Title: Spinal cord injury | **174** |
| Method: Systematic Review | |
| Published since: 2015 | |

Attachment to: Irrgang S, Himmelhaus S, Allek K, Bartholet C, Bersch-Porada I, Curt A, Huber B, Kuhn D, Kynast K, Weidner N, Scheel-Sailer A. Rehabilitation of the lower extremities, standing and walking function in people with spinal cord injury or disease: Guideline of the German-Speaking Medical Society for Spinal Cord Injury. GMS Ger Med Sci. 2025;23:Doc02. DOI: 10.3205/000338, URN: urn:nbn:de:0183-0003383

### A.1.2.4 6-Minute Walking Test

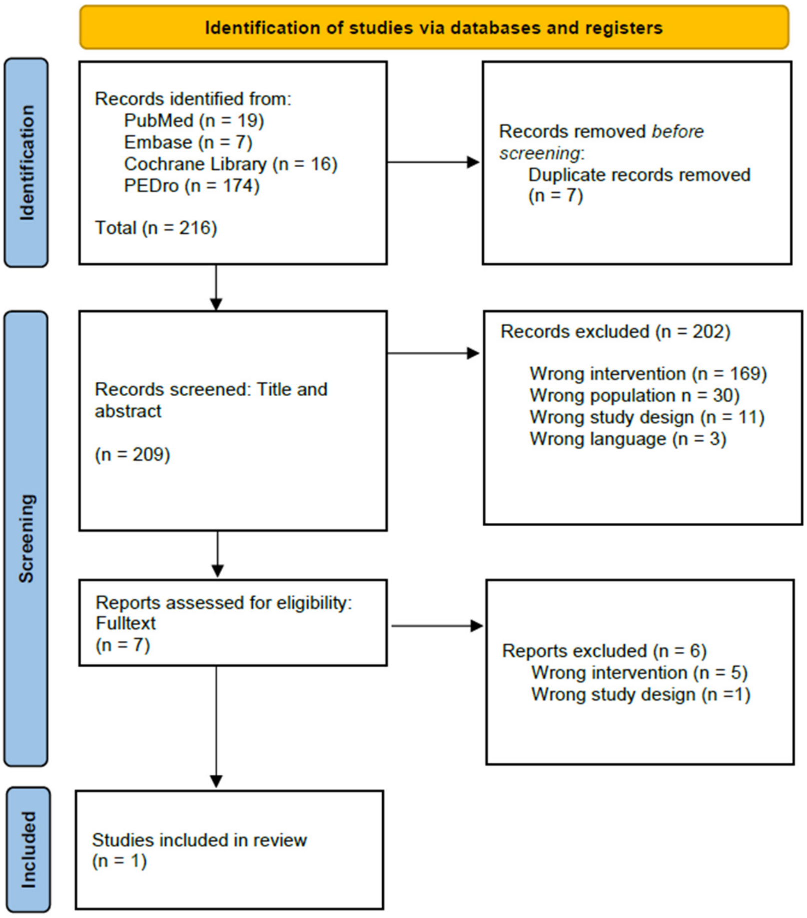


**Figure S5: Flow Chart 6MWT**

**Adapted from Page et al. [1], licensed under CC BY 4.0 (https://creativecommons.org/licenses/by/4.0/)**

**Table A 17: PubMed search 6MWT**

| # | Concept Block | PubMed Search | Results 11/10/2023 |
|---|---|---|---|
| **1** | Spinal Cord Injury (P) | "spinal cord injur*" OR "parapleg*" OR "Paraplegia"[MeSH Terms] OR "tetrapleg*" OR "quadripleg*" OR "Quadriplegia"[MeSH Terms] OR "Spinal Cord Injuries"[MeSH Terms] AND (humans[Filter]) AND (english[Filter] OR german[Filter]) | 65,671 |
| **2** | Assessments (I) | „assessment*“ OR „validation*“ OR "valid*" OR „outcome measure*”OR "Exercise Test"[Mesh] OR "Outcome and Process Assessment, Health Care"[Mesh] OR "Validation Studies as Topic"[Mesh] OR "Prognosis"[Mesh] OR "Classification"[Mesh] OR "Evidence-Based Medicine”[Mesh] OR "Neurologic Examination"[Mesh] OR "Physical Examination"[Mesh] AND (humans[Filter]) AND (english[Filter] OR german[Filter]) | 4,245,199 |
| **3** | Assessment (II) | "6MWT" OR "6 MWT" OR "*walking test" OR "six minute walking test" OR "6 minute walking test" AND (humans[Filter]) AND (english[Filter] OR german[Filter]) | 6,060 |
| **4** | Study type | validation studies OR instrument validation OR external validity OR internal validity OR criterion-related validity OR concurrent validity OR discriminant validity OR content validity OR face validity OR predictive validity OR reliability OR inter-rater reliability OR intra-rater reliability OR test-retest reliability OR reproducibility OR responsiveness OR sensitivity to change OR evidence-based AND (humans[Filter]) AND (english[Filter] OR german[Filter]) | 2,804,170 |
| **5** | Filter | 2015–2023 | |
| **6** | | (#1 AND #2 AND #3 AND #4) AND #5 | **19** |

**Table A 18: Embase search 6MWT**

| # | Concept Block | Embase Search | Results 11/10/2023 |
|---|---|---|---|
| **1** | Spinal Cord Injury | 'spinal cord injury'/exp OR 'paraplegia'/exp OR 'quadriplegia'/exp OR "spinal cord injur*" OR "parapleg*" OR "tetrapleg*" OR "quadripleg*" AND ('human'/de) AND ([english]/lim OR [german]/lim) | 110,479 |
| **2** | Assessments (I) | “assessment*” OR „validation*“ OR "valid*" OR „outcome measure*”OR 'diagnostic test'/exp OR 'outcome assessment'/exp OR 'clinical classification'/exp OR 'evidence based medicine'/exp AND ('human'/de) AND ([english]/lim OR [german]/lim) | 5,943,177 |
| **3** | Assessment (II) | "6MWT" OR "6 MWT" OR "*walking test" OR "six minute walking test" OR "6 minute walking test" AND ('human'/de) AND ([english]/lim OR [german]/lim) | 15,808 |
| **4** | Study type | validation studies OR instrument validation OR external validity OR internal validity OR criterion-related validity OR concurrent validity OR discriminant validity OR content validity OR face validity OR predictive validity OR reliability OR inter-rater reliability OR intra-rater reliability OR test-retest reliability OR reproducibility OR responsiveness OR sensitivity to change OR evidence-based AND ('human'/de) AND ([english]/lim OR [german]/lim) | 424,855 |
| **5** | Filter | 2015–2023 | |
| **6** | | (#1 AND #2 AND #3 AND #4) AND #5 | **7** |

**Table A 19: Cochrane search 6MWT**

| # | Concept Block | Cochrane Library Search | Results 11/10/2023 |
|---|---|---|---|
| **1** | Spinal Cord Injury | spinal cord injur* OR parapleg* OR tetrapleg* OR quadripleg* | 5278 |
| **2** | | MeSH descriptor: [Spinal Cord Injuries] explode all trees | 2297 |
| **3** | | MeSH descriptor: [Paraplegia] explode all trees | 291 |
| **4** | | MeSH descriptor: [Quadriplegia] explode all trees | 243 |
| **5** | Assessments (I) | assessment* OR validation* OR valid* OR outcome measure* | 553197 |
| **6** | | MeSH descriptor: [Diagnostic Tests, Routine] explode all trees | 337 |
| **7** | | MeSH descriptor: [Outcome Assessment, Health Care] explode all trees | 192216 |
| **8** | | MeSH descriptor: [Evidence-Based Medicine] explode all trees | 2977 |
| **9** | Assessment (II) | 6MWT OR 6 MWT OR *walking test OR six minute walking test OR 6 minute walking test | 14830 |
| **10** | Study type | validation studies OR instrument validation OR external validity OR internal validity OR criterion-related validity OR concurrent validity OR discriminant validity OR content validity OR face validity OR predictive validity OR reliability OR inter-rater reliability OR intra-rater reliability OR test-retest reliability OR reproducibility OR responsiveness OR sensitivity to change OR evidence-based | 74035 |
| **11** | Limits | with Cochrane Library publication date from Jan 2015 to Mar 2023, in Cochrane Reviews and Trials | |
| **12** | | ((#1 OR #2 OR #3 OR #4) AND (#5 OR #6 OR #7 OR #8) AND (#9 AND #10) AND #11 | **16** |

**Table A 20: PEDro search 6MWT**

| **PEDro Search** | |
|---|---|
| Abstract & Title: Spinal cord injury | **174** |
| Method: Systematic Review | |
| Published since: 2015 | |

### A.1.2.5 Berg Balance Scale

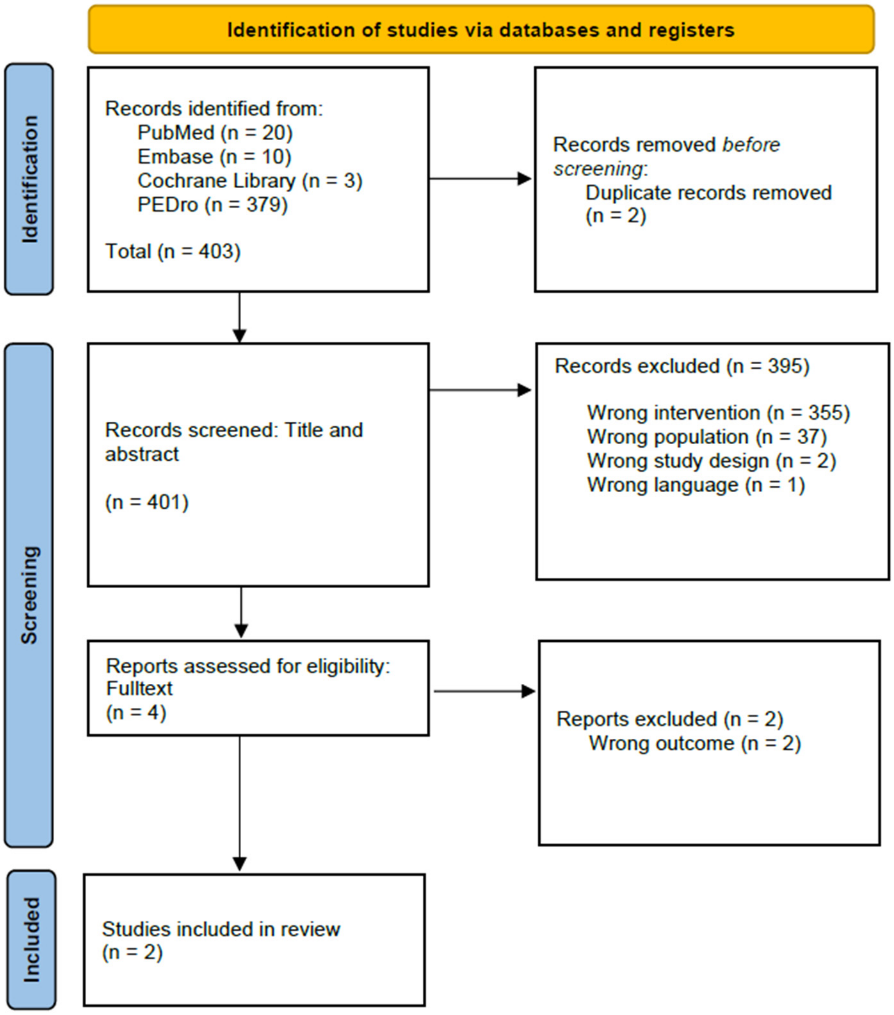


**Figure S6: Flow Chart BBS**

**Adapted from Page et al. [1], licensed under CC BY 4.0 (https://creativecommons.org/licenses/by/4.0/)**

**Table A 21: PubMed search BBS**

| # | Concept Block | PubMed Search | Results 03/11/2023 |
|---|---|---|---|
| **1** | Spinal Cord Injury (P) | "spinal cord injur*" OR "parapleg*" OR "Paraplegia"[MeSH Terms] OR "tetrapleg*" OR "quadripleg*" OR "Quadriplegia"[MeSH Terms] OR "Spinal Cord Injuries"[MeSH Terms] AND (humans[Filter]) AND (english[Filter] OR german[Filter]) | 65,813 |
| **2** | Assessments (I) | „assessment*“ OR „validation*“ OR "valid*" OR „outcome measure*”OR "Exercise Test"[Mesh] OR "Outcome and Process Assessment, Health Care"[Mesh] OR "Validation Studies as Topic"[Mesh] OR "Prognosis"[Mesh] OR "Classification"[Mesh] OR "Evidence-Based Medicine”[Mesh] OR "Neurologic Examination"[Mesh] OR "Physical Examination"[Mesh] AND (humans[Filter]) AND (english[Filter] OR german[Filter]) | 4,258,746 |
| **3** | Assessment (II) | (“berg balance scale” OR “berg balance*” OR BBS) AND (humans[Filter]) AND (english[Filter] OR german[Filter]) | 6,443 |
| **4** | Study type | validation studies OR instrument validation OR external validity OR internal validity OR criterion-related validity OR concurrent validity OR discriminant validity OR content validity OR face validity OR predictive validity OR reliability OR inter-rater reliability OR intra-rater reliability OR test-retest reliability OR reproducibility OR responsiveness OR “sensitivity to change” OR evidence-based AND (humans[Filter]) AND (english[Filter] OR german[Filter]) | 2,706,118 |
| **5** | | (#1 AND #2 AND #3 AND #4) | **20** |

**Table A 22: Embase search BBS**

| # | Concept Block | Embase Search | Results 03/11//2023 |
|---|---|---|---|
| **1** | Spinal Cord Injury | 'spinal cord injury'/exp OR 'paraplegia'/exp OR 'quadriplegia'/exp OR "spinal cord injur*" OR "parapleg*" OR "tetrapleg*" OR "quadripleg*" AND ('human'/de) AND ([english]/lim OR [german]/lim) | 110,748 |
| **2** | Assessments (I) | “assessment*” OR „validation*“ OR "valid*" OR „outcome measure*” OR 'diagnostic test'/exp OR 'outcome assessment'/exp OR 'clinical classification'/exp OR 'evidence based medicine'/exp AND ('human'/de) AND ([english]/lim OR [german]/lim) | 5,969,831 |
| **3** | Assessment (II) | (“Berg Balance Scale” OR “Berg Balance*” OR BBS) AND ('human'/de) AND ([english]/lim OR [german]/lim) | 3,023 |
| **4** | Study type | validation studies OR instrument validation OR external validity OR internal validity OR criterion-related validity OR concurrent validity OR discriminant validity OR content validity OR face validity OR predictive validity OR reliability OR inter-rater reliability OR intra-rater reliability OR test-retest reliability OR reproducibility OR responsiveness OR sensitivity to change OR evidence-based AND ('human'/de) AND ([english]/lim OR [german]/lim) | 426,582 |
| **5** | | #1 AND #2 AND #3 AND #4 | **10** |

**Table A 23: Cochrane search BBS**

| # | Concept Block | Cochrane Library Search | Results 03/11/2023 |
|---|---|---|---|
| **1** | Spinal Cord Injury | spinal cord injur* OR parapleg* OR tetrapleg* OR quadripleg* | 4,686 |
| **2** | | MeSH descriptor: [Spinal Cord Injuries] explode all trees | 2,302 |
| **3** | | MeSH descriptor: [Paraplegia] explode all trees | 293 |
| **4** | | MeSH descriptor: [Quadriplegia] explode all trees | 243 |
| **5** | Assessments (I) | assessment* OR validation* OR valid* OR outcome measure* | 567,229 |
| **6** | | MeSH descriptor: [Diagnostic Test, Routine] explode all trees | 337 |
| **7** | | MeSH descriptor: [Outcome Assessment, Health Care] explode all trees | 192,508 |
| **8** | | MeSH descriptor: [Evidence-Based Medicine] explode all trees | 2,978 |
| **9** | Assessment (II) | "Berg Balance Scale" OR "Berg Balance" OR BBS | 2,952 |
| **10** | Study type | validation studies OR instrument validation OR external validity OR internal validity OR criterion-related validity OR concurrent validity OR discriminant validity OR content validity OR face validity OR predictive validity OR reliability OR inter-rater reliability OR intra-rater reliability OR test-retest reliability OR reproducibility OR responsiveness OR sensitivity to change OR evidence-based | 91,899 |
| **11** | Limits | | |
| **12** | | ((#1 OR #2 OR #3 OR #4) AND (#5 OR #6 OR #7 OR #8) AND (#9 AND #10) AND #11 | **3** |

**Table A 24: PEDro search BBS**

| **PEDro Search** | |
|---|---|
| Abstract & Title: Spinal cord injury | **379** |
| Method: Clinical trial | |

### A.1.2.6 Mini-BESTest

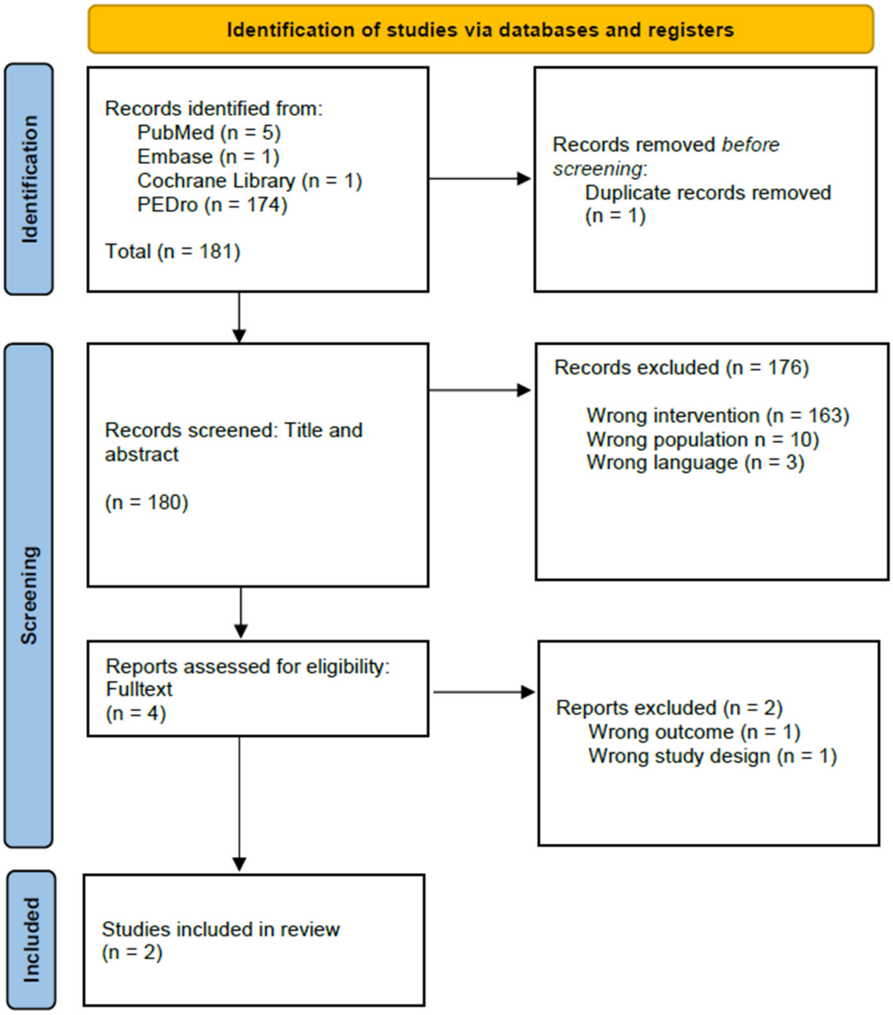


**Figure S7: Flow Chart Mini-BESTest**

**Adapted from Page et al. [1], licensed under CC BY 4.0 (https://creativecommons.org/licenses/by/4.0/)**

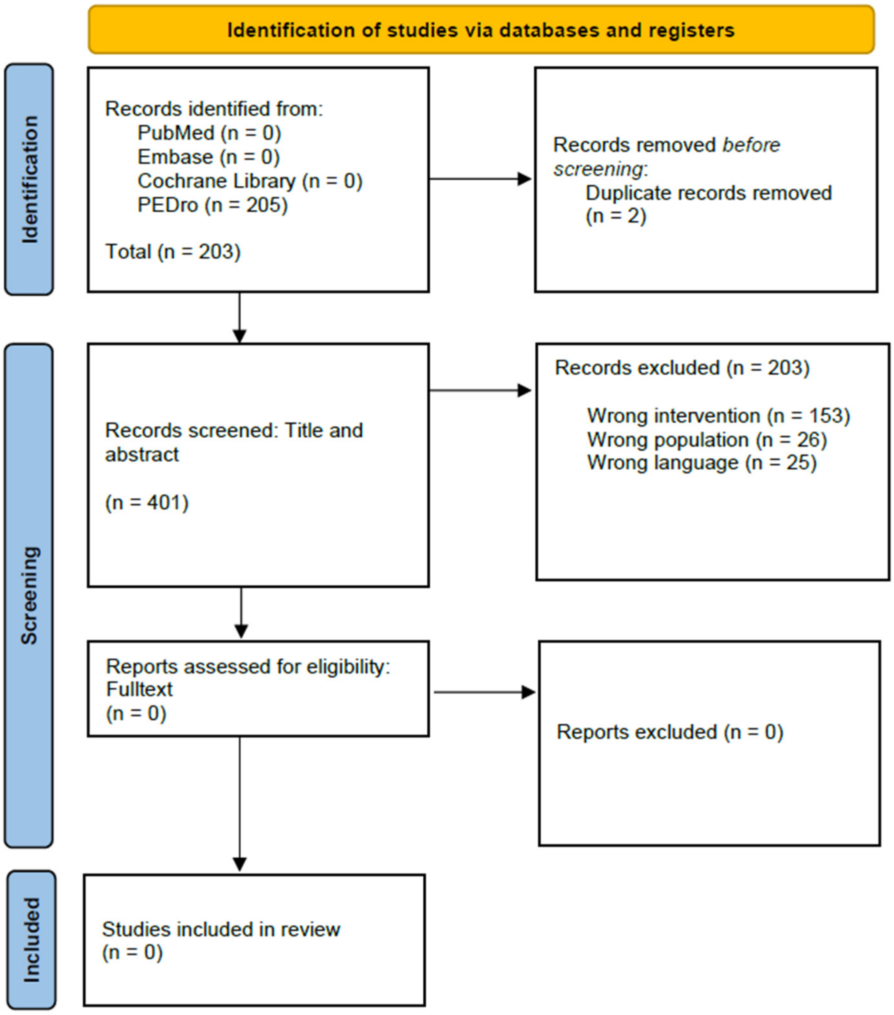


**Figure S8: Flow Chart Mini-BESTest**

**Adapted from Page et al. [1], licensed under CC BY 4.0 (https://creativecommons.org/licenses/by/4.0/)**

**Table A 25: PubMed search Mini-BESTest**

| # | Concept Block | PubMed Search | Results 11/10/2023 (03/11/2023) |
|---|---|---|---|
| **1** | Spinal Cord Injury (P) | "spinal cord injur*" OR "parapleg*" OR "Paraplegia"[MeSH Terms] OR "tetrapleg*" OR "quadripleg*" OR "Quadriplegia"[MeSH Terms] OR "Spinal Cord Injuries"[MeSH Terms] AND (humans[Filter]) AND (english[Filter] OR german[Filter]) | 65,671 (65,787) |
| **2** | Assessments (I) | „assessment*" OR „validation*" OR "valid*" OR „outcome measure*"OR "Exercise Test"[Mesh] OR "Outcome and Process Assessment, Health Care"[Mesh] OR "Validation Studies as Topic"[Mesh] OR "Prognosis"[Mesh] OR "Classification"[Mesh] OR "Evidence-Based Medicine"[Mesh] OR "Neurologic Examination"[Mesh] OR "Physical Examination"[Mesh] AND (humans[Filter]) AND (english[Filter] OR german[Filter]) | 4,245,199 (4,255,646) |
| **3** | Assessment (II) | Mini Bes Test OR "Mini-BESTest" OR "Mini-Balance Evaluation Systems Test" AND (humans[Filter]) AND (english[Filter] OR german[Filter]) | 242 (244) |
| **4** | Study type | validation studies OR instrument validation OR external validity OR internal validity OR criterion-related validity OR concurrent validity OR discriminant validity OR content validity OR face validity OR predictive validity OR reliability OR inter-rater reliability OR intra-rater reliability OR test-retest reliability OR reproducibility OR responsiveness OR sensitivity to change OR evidence-based AND (humans[Filter]) AND (english[Filter] OR german[Filter]) | 2,804,170 (2,811,803) |
| **5** | Filter | 2015–2023 (bis 2015) | |
| **6** | | (#1 AND #2 AND #3 AND #4) AND #5 | **5 (0)** |

**Table A 26: Embase search Mini-BESTest**

| # | Concept Block | Embase Search | Results 11/10/2023 (03/11/2023) |
|---|---|---|---|
| **1** | Spinal Cord Injury | 'spinal cord injury'/exp OR 'paraplegia'/exp OR 'quadriplegia'/exp OR "spinal cord injur*" OR "parapleg*" OR "tetrapleg*" OR "quadripleg*" AND ('human'/de) AND ([english]/lim OR [german]/lim) | 110,479 (110,742) |
| **2** | Assessments (I) | "assessment*" OR „validation*" OR "valid*" OR „outcome measure*" OR 'diagnostic test'/exp OR 'outcome assessment'/exp OR 'clinical classification'/exp OR 'evidence based medicine'/exp AND ('human'/de) AND ([english]/lim OR [german]/lim) | 5,943,177 (5.968,698) |
| **3** | Assessment (II) | Mini Bes Test OR "Mini-BESTest" OR "Mini-Balance Evaluation Systems Test" AND ('human'/de) AND ([english]/lim OR [german]/lim) | 509 (515) |
| **4** | Study type | validation studies OR instrument validation OR external validity OR internal validity OR criterion-related validity OR concurrent validity OR discriminant validity OR content validity OR face validity OR predictive validity OR reliability OR inter-rater reliability OR intra-rater reliability OR test-retest reliability OR reproducibility OR responsiveness OR sensitivity to change OR evidence-based AND ('human'/de) AND ([english]/lim OR [german]/lim) | 424,855 (426,515) |
| **5** | Filter | #1 AND #2 AND #3 AND #4 | 1 (1) |
| **6** | | 2015–2023 (<1996–2015) | **1 (0)** |

**Table A 27: Cochrane search Mini-BESTest**

| # | Concept Block | Cochrane Library Search | Results 11/10/2023 (03/11/2023) |
|---|---|---|---|
| **1** | Spinal Cord Injury | spinal cord injury OR paraplegia OR tetraplegia OR quadriplegia | 5,278 (4,686) |
| **2** | | MeSH descriptor: [Spinal Cord Injuries] explode all trees | 2,297 (2,302) |
| **3** | | MeSH descriptor: [Paraplegia] explode all trees | 291 (293) |
| **4** | | MeSH descriptor: [Quadriplegia] explode all trees | 243 (243) |
| **5** | Assessments (I) | assessment* OR validation* OR valid* OR outcome measure* | 553,197 (447,050) |
| **6** | | MeSH descriptor: [Diagnostic Tests, Routine] explode all trees | 337 (310,165) |
| **7** | | MeSH descriptor: [Outcome Assessment, Health Care] explode all trees | 192,216 (192,508) |
| **8** | | MeSH descriptor: [Evidence-Based Medicine] explode all trees | 2,977 (2,978) |
| **9** | Assessment (II) | Mini Bes Test OR Mini-BESTest OR Mini-Balance Evaluation Systems Test | 267 (300) |
| **10** | Study type | validation studies OR instrument validation OR external validity OR internal validity OR criterion-related validity OR concurrent validity OR discriminant validity OR content validity OR face validity OR predictive validity OR reliability OR inter-rater reliability OR intra-rater reliability OR test-retest reliability OR reproducibility OR responsiveness OR sensitivity to change OR evidence-based | 74,035 (91,898) |
| **11** | Limits | with Cochrane Library publication date from Jan 2015 to Mar 2023, in Cochrane Reviews and Trials (with Cochrane Library publication date to Jan 2015, in Cochrane Reviews and Trials) | |
| **12** | | ((#1 OR #2 OR #3 OR #4) AND (#5 OR #6 OR #7 OR #8) AND (#9 AND #10) AND #11 | **1 (0)** |

**Table A 28: PEDro search Mini-BESTest**

| **PEDro Search** | |
|---|---|
| Abstract & Title: Spinal cord injury | **379** |
| Method: Clinical trial | |
| Published since: 2015 | |
| Mit Rayyan gefiltert vor 2015 | **205** |

### A.1.2.7 Functional Reach Test / modified Functional Reach Test

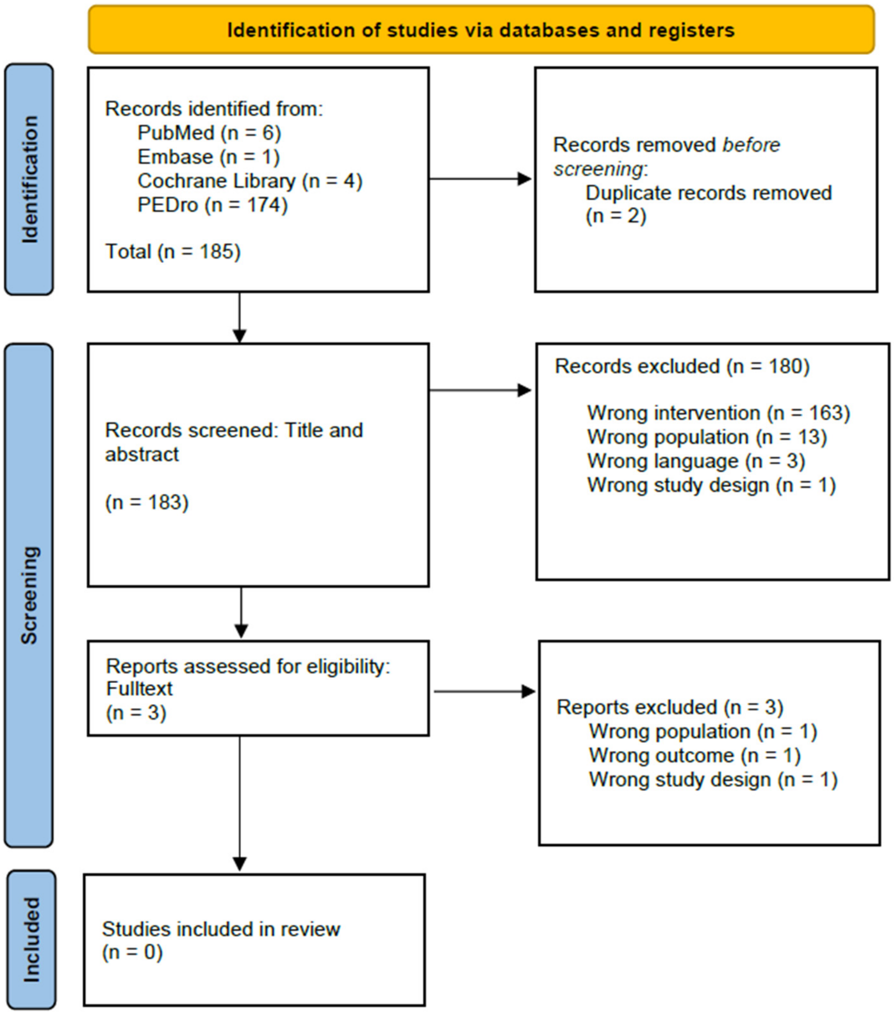


**Figure S9: Flow Chart FRT/mFRT**

**Adapted from Page et al. [1], licensed under CC BY 4.0 (https://creativecommons.org/licenses/by/4.0/)**

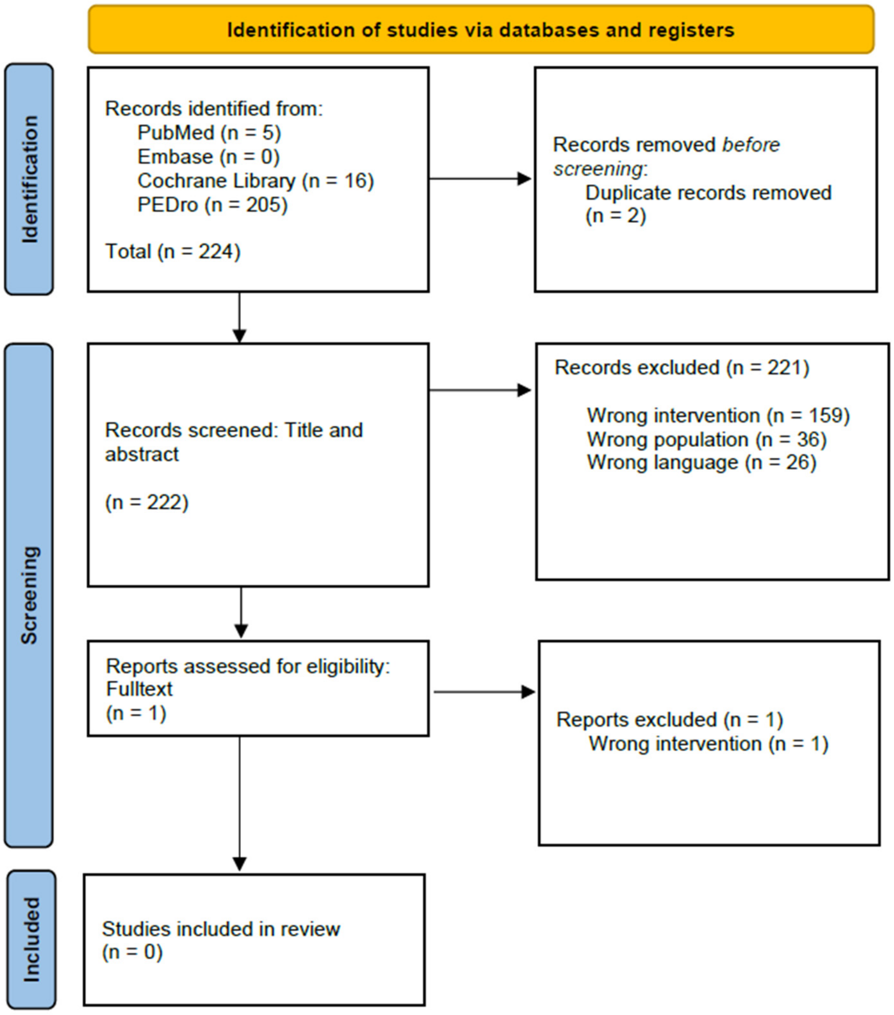


**Figure S10: Flow Chart FRT/mFRT**

**Adapted from Page et al. [1], licensed under CC BY 4.0 (https://creativecommons.org/licenses/by/4.0/)**

**Table A 29: PubMed search FRT/mFRT**

| # | Concept Block | PubMed Search | Results 11/10/2023 (03/11/2023) |
|---|---|---|---|
| **1** | Spinal Cord Injury (P) | "spinal cord injur*" OR "parapleg*" OR "Paraplegia"[MeSH Terms] OR "tetrapleg*" OR "quadripleg*" OR "Quadriplegia"[MeSH Terms] OR "Spinal Cord Injuries"[MeSH Terms] AND (humans[Filter]) AND (english[Filter] OR german[Filter]) | 65,671 (65,813) |
| **2** | Assessments (I) | „assessment*" OR „validation*" OR "valid*" OR „outcome measure*"OR "Exercise Test"[Mesh] OR "Outcome and Process Assessment, Health Care"[Mesh] OR "Validation Studies as Topic"[Mesh] OR "Prognosis"[Mesh] OR "Classification"[Mesh] OR "Evidence-Based Medicine"[Mesh] OR "Neurologic Examination"[Mesh] OR "Physical Examination"[Mesh] AND (humans[Filter]) AND (english[Filter] OR german[Filter]) | 4,245,199 (4,258,746) |
| **3** | Assessment (II) | "FRT" OR "mFRT" OR "Functional Reach Test" OR "modified Functional Reach Test" OR "*Reach Test" AND (humans [Filter]) AND (english[Filter] OR german[Filter]) | 1,641 (1,650) |
| **4** | Study type | validation studies OR instrument validation OR external validity OR internal validity OR criterion-related validity OR concurrent validity OR discriminant validity OR content validity OR face validity OR predictive validity OR reliability OR inter-rater reliability OR intra-rater reliability OR test-retest reliability OR reproducibility OR responsiveness OR "sensitivity to change" OR evidence-based AND (humans[Filter]) AND (english[Filter] OR german[Filter]) | 2,804,170 (2,706,118) |
| **5** | Filter | 2015–2023 (1000/1/1–2015/1/1) | |
| **6** | | (#1 AND #2 AND #3 AND #4) AND #5 | **6 (5)** |

**Table A 30: Embase search FRT/mFRT**

| # | Concept Block | Embase Search | Results 11/10/2023 (03/11/2023) |
|---|---|---|---|
| **1** | Spinal Cord Injury | 'spinal cord injury'/exp OR 'paraplegia'/exp OR 'quadriplegia'/exp OR "spinal cord injur*" OR "parapleg*" OR "tetrapleg*" OR "quadripleg*" AND ('human'/de) AND ([english]/lim OR [german]/lim) | 110,479 (110,748) |
| **2** | Assessments (I) | "assessment*" OR „validation*" OR "valid*" OR „outcome measure*"OR 'diagnostic test'/exp OR 'outcome assessment'/exp OR 'clinical classification'/exp OR 'evidence based medicine'/exp AND ('human'/de) AND ([english]/lim OR [german]/lim) | 5,943,177 (5,969,831) |
| **3** | Assessment (II) | "FRT" OR "mFRT" OR "Functional Reach Test" OR "modified Functional Reach Test" OR "Reach Test" AND ('human'/de) AND ([english]/lim OR [german]/lim) | 3,014 (3,023) |
| **4** | Study type | validation studies OR instrument validation OR external validity OR internal validity OR criterion-related validity OR concurrent validity OR discriminant validity OR content validity OR face validity OR predictive validity OR reliability OR inter-rater reliability OR intra-rater reliability OR test-retest reliability OR reproducibility OR responsiveness OR sensitivity to change OR evidence-based AND ('human'/de) AND ([english]/lim OR [german]/lim) | 424,855 (426,582) |
| **5** | Filter | #1 AND #2 AND #3 AND #4 | **1 (10)** |
| **6** | | 2015–2023 (<1966–2015) | **1 (0)** |

**Table A 31: Cochrane search FRT/mFRT**

| # | Concept Block | Cochrane Library Search | Results 11/10/2023 (03/11/2023) |
|---|---|---|---|
| **1** | Spinal Cord Injury | spinal cord injur* OR parapleg* OR tetrapleg* OR quadripleg* | 5,278 (4,686) |
| **2** | | MeSH descriptor: [Spinal Cord Injuries] explode all trees | 2,297 (2,302) |
| **3** | | MeSH descriptor: [Paraplegia] explode all trees | 291 (293) |
| **4** | | MeSH descriptor: [Quadriplegia] explode all trees | 243 (243) |
| **5** | Assessments (I) | assessment* OR validation* OR valid* OR outcome measure* | 553,197 (567,229) |
| **6** | | MeSH descriptor: [Diagnostic Tests, Routine] explode all trees | 337 (337) |
| **7** | | MeSH descriptor: [Outcome Assessment, Health Care] explode all trees | 192,216 (192,508) |
| **8** | | MeSH descriptor: [Evidence-Based Medicine] explode all trees | 2,977 (2,978) |
| **9** | Assessment (II) | FRT OR mFRT OR Functional Reach Test OR modified Functional Reach Test OR *Reach Test | 7,845 (11,072) |
| **10** | Study type | validation studies OR instrument validation OR external validity OR internal validity OR criterion-related validity OR concurrent validity OR discriminant validity OR content validity OR face validity OR predictive validity OR reliability OR inter-rater reliability OR intra-rater reliability OR test-retest reliability OR reproducibility OR responsiveness OR sensitivity to change OR evidence-based | 74,035 (91,899) |
| **11** | Limits | with Cochrane Library publication date from Jan 2015 to Mar 2023, in Cochrane Reviews and Trials (with Cochrane Library publication date to Jan 2015, in Cochrane Reviews and Trials | |
| **12** | | ((#1 OR #2 OR #3 OR #4) AND (#5 OR #6 OR #7 OR #8) AND (#9 AND #10)) AND #11 | **4 (16)** |

**Table A 32: PEDro search FRT/mFRT**

| **PEDro Search** | |
|---|---|
| Abstract & Title: Spinal cord injury | **379** |
| Method: Clinical trial | |
| Published since: 2015 | |
| Mit Rayyan gefiltert vor 2015 | **205** |

### A.1.2.8 Penn Spasm Frequency Scale

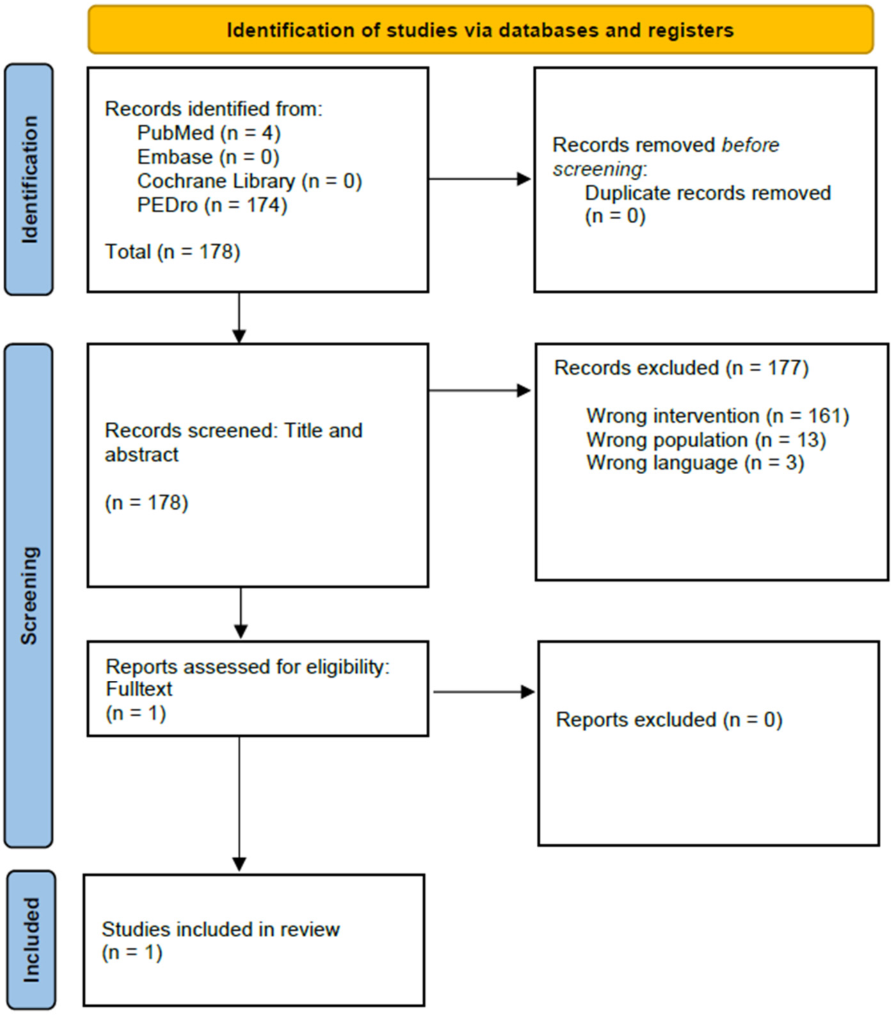


**Figure S11: Flow Chart PSFS**

**Adapted from Page et al. [1], licensed under CC BY 4.0 (https://creativecommons.org/licenses/by/4.0/)**

**Table A 33: PubMed search PSFS**

| # | Concept Block | PubMed Search | Results 11/10/2023 |
|---|---|---|---|
| **1** | Spinal Cord Injury (P) | "spinal cord injur*" OR "parapleg*" OR "Paraplegia"[MeSH Terms] OR "tetrapleg*" OR "quadripleg*" OR "Quadriplegia"[MeSH Terms] OR "Spinal Cord Injuries"[MeSH Terms] AND (humans[Filter]) AND (english[Filter] OR german[Filter]) | 65,671 |
| **2** | Assessments (I) | „assessment*" OR „validation*" OR "valid*" OR „outcome measure*"OR "Exercise Test"[Mesh] OR "Outcome and Process Assessment, Health Care"[Mesh] OR "Validation Studies as Topic"[Mesh] OR "Prognosis"[Mesh] OR "Classification"[Mesh] OR "Evidence-Based Medicine"[Mesh] OR "Neurologic Examination"[Mesh] OR "Physical Examination"[Mesh] AND (humans[Filter]) AND (english[Filter] OR german[Filter]) | 4,245,199 |
| **3** | Assessment (II) | "Penn Spasm Frequency Scale" OR Penn Frequency Scale OR "PSFS" AND (humans[Filter]) AND (english[Filter] OR german[Filter]) | 842 |
| **4** | Study type | validation studies OR instrument validation OR external validity OR internal validity OR criterion-related validity OR concurrent validity OR discriminant validity OR content validity OR face validity OR predictive validity OR reliability OR inter-rater reliability OR intra-rater reliability OR test-retest reliability OR reproducibility OR responsiveness OR sensitivity to change OR evidence-based AND (humans[Filter]) AND (english[Filter] OR german[Filter]) | 2,804,170 |
| **5** | Filter | 2015–2023 | |
| **6** | | (#1 AND #2 AND #3 AND #4) AND #5 | **4** |

**Table A 34: Embase search PSFS**

| # | Concept Block | Embase Search | Results 11/10/2023 |
|---|---|---|---|
| **1** | Spinal Cord Injury | 'spinal cord injury'/exp OR 'paraplegia'/exp OR 'quadriplegia'/exp OR "spinal cord injur*" OR "parapleg*" OR "tetrapleg*" OR "quadripleg*" AND ('human'/de) AND ([english]/lim OR [german]/lim) | 110,479 |
| **2** | Assessments (I) | "assessment*" OR „validation*" OR "valid*" OR „outcome measure*"OR 'diagnostic test'/exp OR 'outcome assessment'/exp OR 'clinical classification'/exp OR 'evidence based medicine'/exp AND ('human'/de) AND ([english]/lim OR [german]/lim) | 5,943,177 |
| **3** | Assessment (II) | "Penn Spasm Frequency Scale" OR Penn Frequency Scale OR "PSFS" AND ('human'/de) AND ([english]/lim OR [german]/lim) | 864 |
| **4** | Study type | validation studies OR instrument validation OR external validity OR internal validity OR criterion-related validity OR concurrent validity OR discriminant validity OR content validity OR face validity OR predictive validity OR reliability OR inter-rater reliability OR intra-rater reliability OR test-retest reliability OR reproducibility OR responsiveness OR sensitivity to change OR evidence-based AND ('human'/de) AND ([english]/lim OR [german]/lim) | 424,855 |
| **5** | Filter | 2015–2023 | 0 |
| **6** | | (#1 AND #2 AND #3 AND #4) AND #5 | **0** |

**Table A 35: Cochrane search PSFS**

| # | Concept Block | Cochrane Library Search | Results 11/10/2023 |
|---|---|---|---|
| **1** | Spinal Cord Injury | spinal cord injur* OR parapleg* OR tetrapleg* OR quadripleg* | 5,278 |
| **2** | | MeSH descriptor: [Spinal Cord Injuries] explode all trees | 2,297 |
| **3** | | MeSH descriptor: [Paraplegia] explode all trees | 291 |
| **4** | | MeSH descriptor: [Quadriplegia] explode all trees | 243 |
| **5** | Assessments (I) | assessment* OR validation* OR valid* OR outcome measure* | 553,197 |
| **6** | | MeSH descriptor: [Diagnostic Tests, Routine] explode all trees | 337 |
| **7** | | MeSH descriptor: [Outcome Assessment, Health Care] explode all trees | 192,216 |
| **8** | | MeSH descriptor: [Evidence-Based Medicine] explode all trees | 2,977 |
| **9** | Assessment (II) | Penn Spasm Frequency Scale OR Penn Frequency Scale OR PSFS | 250 |
| **10** | Study type | validation studies OR instrument validation OR external validity OR internal validity OR criterion-related validity OR concurrent validity OR discriminant validity OR content validity OR face validity OR predictive validity OR reliability OR inter-rater reliability OR intra-rater reliability OR test-retest reliability OR reproducibility OR responsiveness OR sensitivity to change OR evidence-based | 74,035 |
| **11** | Limits | with Cochrane Library publication date from Jan 2015 to Mar 2023, in Cochrane Reviews and Trials | |
| **12** | | ((#1 OR #2 OR #3 OR #4) AND (#5 OR #6 OR #7 OR #8) AND (#9 AND #10) AND #11 | **0** |

**Table A 36: PEDro search PSFS**

| **PEDro Search** | |
|---|---|
| Abstract & Title: Spinal cord injury | **174** |
| Method: Systematic Review | |
| Published since: 2015 | |

Attachment to: Irrgang S, Himmelhaus S, Allek K, Bartholet C, Bersch-Porada I, Curt A, Huber B, Kuhn D, Kynast K, Weidner N, Scheel-Sailer A. Rehabilitation of the lower extremities, standing and walking function in people with spinal cord injury or disease: Guideline of the German-Speaking Medical Society for Spinal Cord Injury. GMS Ger Med Sci. 2025;23:Doc02. DOI: 10.3205/000338, URN: urn:nbn:de:0183-0003383

### A.1.2.9 Spinal Cord Assessment Tool for Spastic Reflexes

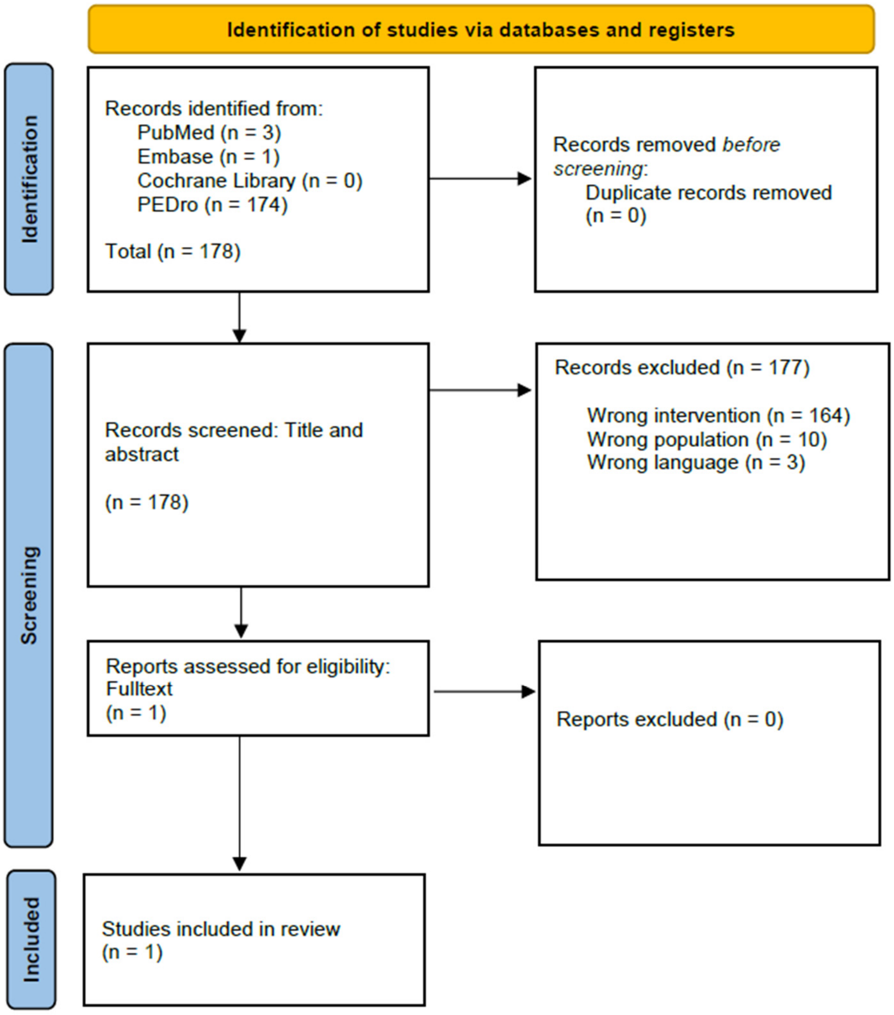


**Figure S12: Flow Chart SCATS**

**Adapted from Page et al. [1], licensed under CC BY 4.0 (https://creativecommons.org/licenses/by/4.0/)**

**Table A 37: PubMed search SCATS**

| # | Concept Block | PubMed Search | Results 11/10/2023 |
|---|---|---|---|
| **1** | Spinal Cord Injury (P) | "spinal cord injur*" OR "parapleg*" OR "Paraplegia"[MeSH Terms] OR "tetrapleg*" OR "quadripleg*" OR "Quadriplegia"[MeSH Terms] OR "Spinal Cord Injuries"[MeSH Terms] AND (humans[Filter]) AND (english[Filter] OR german[Filter]) | 65,671 |
| **2** | Assessments (I) | „assessment*" OR „validation*" OR "valid*" OR „outcome measure*"OR "Exercise Test"[Mesh] OR "Outcome and Process Assessment, Health Care"[Mesh] OR "Validation Studies as Topic"[Mesh] OR "Prognosis"[Mesh] OR "Classification"[Mesh] OR "Evidence-Based Medicine"[Mesh] OR "Neurologic Examination"[Mesh] OR "Physical Examination"[Mesh] AND (humans[Filter]) AND (english[Filter] OR german[Filter]) | 4,245,199 |
| **3** | Assessment (II) | "Spinal Cord Assessment Tool for Spastic Reflexes" OR "SCATS" AND (humans[Filter]) AND (english[Filter] OR german[Filter]) | 53 |
| **4** | Study type | validation studies OR instrument validation OR external validity OR internal validity OR criterion-related validity OR concurrent validity OR discriminant validity OR content validity OR face validity OR predictive validity OR reliability OR inter-rater reliability OR intra-rater reliability OR test-retest reliability OR reproducibility OR responsiveness OR sensitivity to change OR evidence-based AND (humans[Filter]) AND (english[Filter] OR german[Filter]) | 2,804,170 |
| **5** | Filter | 2015–2023 | |
| **6** | | (#1 AND #2 AND #3 AND #4) AND #5 | **3** |

**Table A 38: Embase search SCATS**

| # | Concept Block | Embase Search | Results 11/10/2023 |
|---|---|---|---|
| **1** | Spinal Cord Injury | 'spinal cord injury'/exp OR 'paraplegia'/exp OR 'quadriplegia'/exp OR "spinal cord injur*" OR "parapleg*" OR "tetrapleg*" OR "quadripleg*" AND ('human'/de) AND ([english]/lim OR [german]/lim) | 110,479 |
| **2** | Assessments (I) | "assessment*" OR „validation*" OR "valid*" OR „outcome measure*"OR 'diagnostic test'/exp OR 'outcome assessment'/exp OR 'clinical classification'/exp OR 'evidence based medicine'/exp AND ('human'/de) AND ([english]/lim OR [german]/lim) | 5,943,177 |
| **3** | Assessment (II) | "Spinal Cord Assessment Tool for Spastic Reflexes" OR "SCATS" AND ('human'/de) AND ([english]/lim OR [german]/lim) | 66 |
| **4** | Study type | validation studies OR instrument validation OR external validity OR internal validity OR criterion-related validity OR concurrent validity OR discriminant validity OR content validity OR face validity OR predictive validity OR reliability OR inter-rater reliability OR intra-rater reliability OR test-retest reliability OR reproducibility OR responsiveness OR sensitivity to change OR evidence-based AND ('human'/de) AND ([english]/lim OR [german]/lim) | 424,855 |
| **5** | Filter | #1 AND #2 AND #3 AND #4 | 1 |
| **6** | | 2015–2023 | **1** |

**Table A 39: Cochrane search SCATS**

| # | Concept Block | Cochrane Library Search | Results 11/10/2023 |
|---|---|---|---|
| **1** | Spinal Cord Injury | spinal cord injur* OR parapleg* OR tetrapleg* OR quadripleg* | 5,278 |
| **2** | | MeSH descriptor: [Spinal Cord Injuries] explode all trees | 2,297 |
| **3** | | MeSH descriptor: [Paraplegia] explode all trees | 291 |
| **4** | | MeSH descriptor: [Quadriplegia] explode all trees | 243 |
| **5** | Assessments (I) | assessment* OR validation* OR valid* OR outcome measure* | 553,197 |
| **6** | | MeSH descriptor: [Diagnostic Tests, Routine] explode all trees | 337 |
| **7** | | MeSH descriptor: [Outcome Assessment, Health Care] explode all trees | 192,216 |
| **8** | | MeSH descriptor: [Evidence-Based Medicine] explode all trees | 2,977 |
| **9** | Assessment (II) | Spinal Cord Assessment Tool for Spastic Reflexes OR SCATS | 6 |
| **10** | Study type | validation studies OR instrument validation OR external validity OR internal validity OR criterion-related validity OR concurrent validity OR discriminant validity OR content validity OR face validity OR predictive validity OR reliability OR inter-rater reliability OR intra-rater reliability OR test-retest reliability OR reproducibility OR responsiveness OR sensitivity to change OR evidence-based | 7,4035 |
| **11** | Limits | with Cochrane Library publication date from Jan 2015 to Mar 2023, in Cochrane Reviews and Trials | |
| **12** | | ((#1 OR #2 OR #3 OR #4) AND (#5 OR #6 OR #7 OR #8) AND (#9 AND #10) AND #11 | **0** |

**Table A 40: PEDro search SCATS**

| **PEDro Search** | |
|---|---|
| Abstract & Title: Spinal cord injury | **174** |
| Method: Systematic Review | |
| Published since: 2015 | |

Attachment to: Irrgang S, Himmelhaus S, Allek K, Bartholet C, Bersch-Porada I, Curt A, Huber B, Kuhn D, Kynast K, Weidner N, Scheel-Sailer A. Rehabilitation of the lower extremities, standing and walking function in people with spinal cord injury or disease: Guideline of the German-Speaking Medical Society for Spinal Cord Injury. GMS Ger Med Sci. 2025;23:Doc02. DOI: 10.3205/000338, URN: urn:nbn:de:0183-0003383

## A.1.3 Systematic Search for primary studies on Interventions

### A.1.3.1 ADL-Movementtherapy

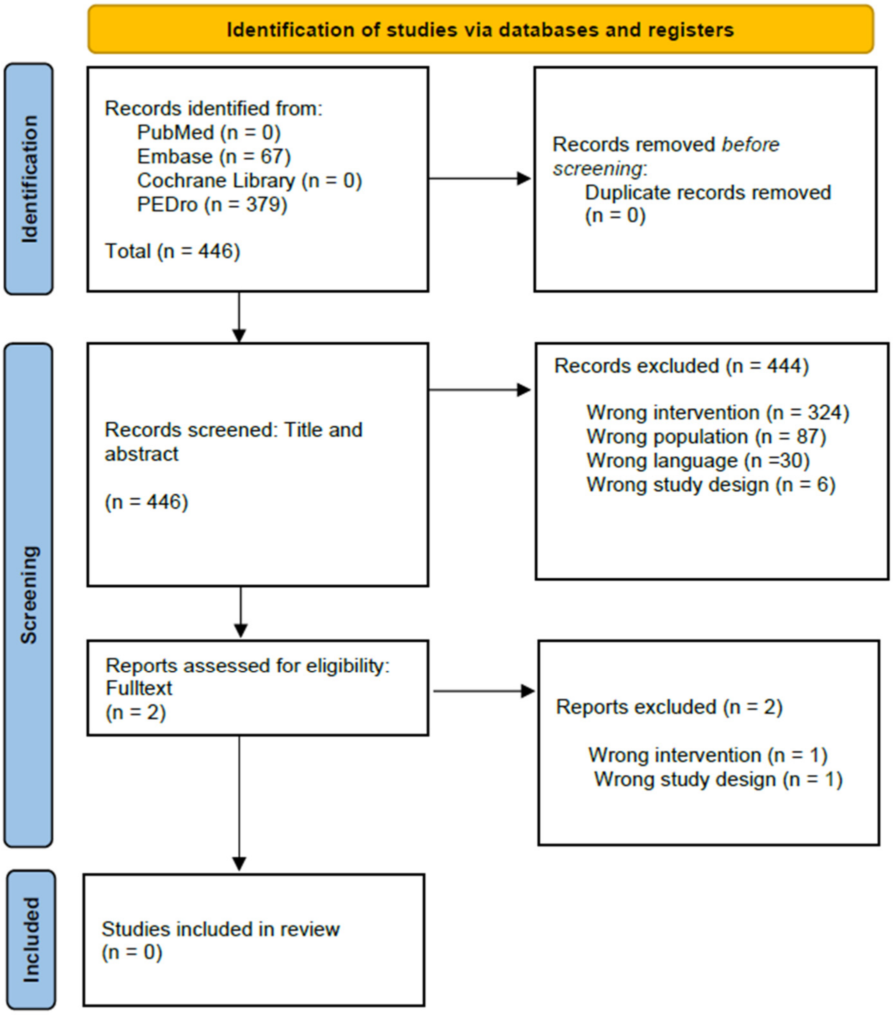


**Figure S13: Flow Chart ADL-Movementtherapy**

**Adapted from Page et al. [1], licensed under CC BY 4.0 (https://creativecommons.org/licenses/by/4.0/)**

**Table A 41: PubMed search ADL-Movementtherapy**

| **#** | **Concept Block** | **PubMed Search** | **Results 07/11/2023** |
|---|---|---|---|
| **1** | Spinal Cord Injury (P) | "spinal cord injur*" OR "parapleg*" OR "Paraplegia"[MeSH Terms] OR "tetrapleg*" OR "quadripleg*" OR "Quadriplegia"[MeSH Terms] OR "Spinal Cord Injuries"[MeSH Terms] AND (humans[Filter]) AND (english[Filter] OR german[Filter]) | 65,830 |
| **2** | Intervention | “ADL training*” OR ADL OR “activities of daily life*” OR Activities of Daily Living [MeSH], Stimulation Training [MeSH], Physical Conditioning, Human [MeSH] AND (humans[Filter]) AND (english[Filter] OR german[Filter]) | 1 |
| **3** | Outcome | “standing*” OR “walking*” OR Standing Position [MeSH], Walking [MeSH], Gait [MeSH], Locomotion [MeSH] AND (humans[Filter]) AND (english[Filter] OR german[Filter]) | 17,833 |
| **4** | | #1 AND #2 AND #3 | **0** |

**Table A 42: Embase search ADL-Movementtherapy**

| # | Concept Block | Embase Search | Results 07/11/2023 |
|---|---|---|---|
| **1** | Spinal Cord Injury | 'spinal cord injury'/exp OR 'paraplegia'/exp OR 'quadriplegia'/exp OR "spinal cord injur*" OR "parapleg*" OR "tetrapleg*" OR "quadripleg*" AND ('human'/de) AND ([english]/lim OR [german]/lim) | 110,791 |
| **2** | Intervention | 'ADL training*" OR ADL OR "activities of daily life*" OR 'ADL disability'/exp AND ('human'/de) AND ([english]/lim OR [german]/lim) | 24,150 |
| **3** | Outcome | "standing*" OR "walking*" OR 'standing'/exp OR 'walking'/exp AND ('human'/de) AND ([english]/lim OR [german]/lim) | 266,998 |
| **4** | | #1 AND #2 AND #3 | **67** |

**Table A 43: Cochrane search ADL-Movementtherapy**

| # | Concept Block | Cochrane Library Search | Results 07/11/2023 |
|---|---|---|---|
| **1** | Spinal Cord Injury | spinal cord injur* OR parapleg* OR tetrapleg* OR quadripleg* | 4,686 |
| **2** | | MeSH descriptor: [Spinal Cord Injuries] explode all trees | 2,302 |
| **3** | | MeSH descriptor: [Paraplegia] explode all trees | 293 |
| **4** | | MeSH descriptor: [Quadriplegia] explode all trees | 243 |
| **5** | Intervention | 'ADL training*" OR ADL OR "activities of daily life*" | 0 |
| **6** | | MeSH descriptor: [Activities of Daily Living] explode all trees | 13,110 |
| **7** | Outcomes | Standing OR walking | 39,854 |
| **8** | | MeSH descriptor: [Standing Position] explode all trees | 118 |
| **9** | | MeSH descriptor: [Walking] explode all trees | 8,525 |
| **10** | Limits | | |
| **11** | | (#1 OR #2 OR #3 OR #4) AND (#5 OR #6) AND (#7 OR #8 OR #9) | **0** |

**Table A 44: PEDro search ADL-Movementtherapy**

| PEDro Search | |
|---|---|
| Abstract & Title: Spinal cord injury | **379** |
| Method: Clinical trial | |

### A.1.3.2 PNF, Vojta, Bobath

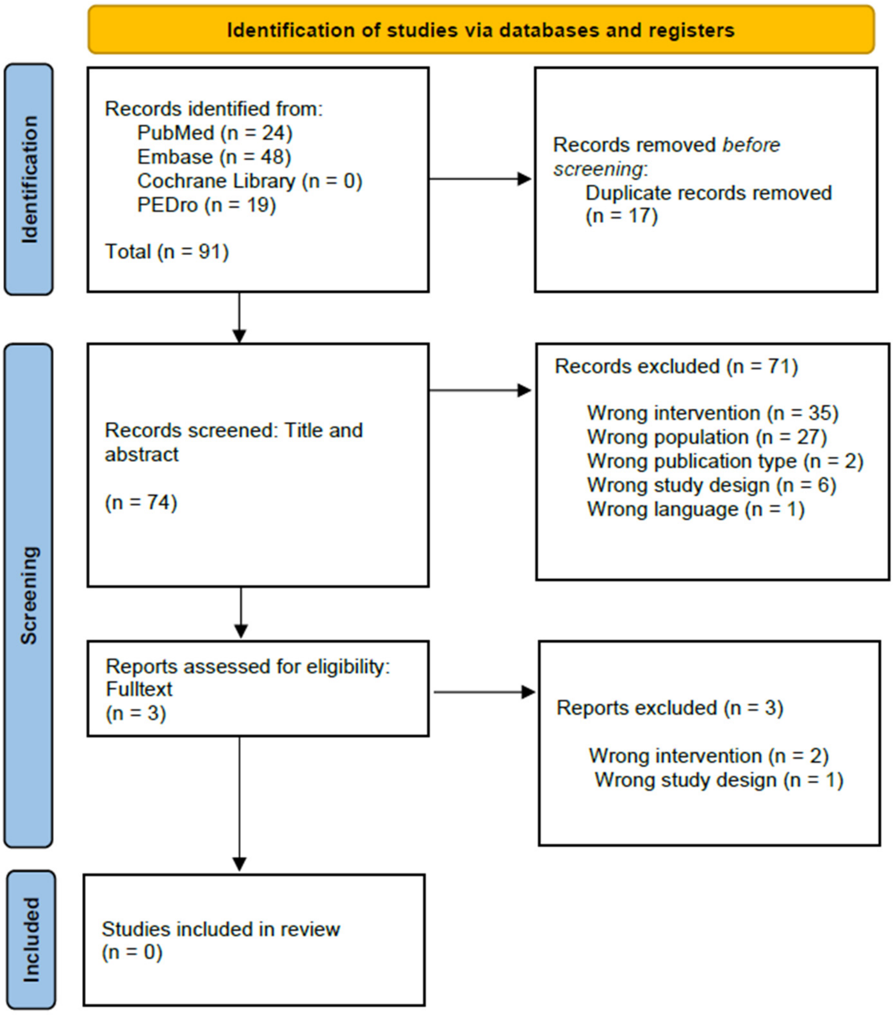


**Figure S14: Flow Chart PNF, Vojta, Bobath**

**Adapted from Page et al. [1], licensed under CC BY 4.0 (https://creativecommons.org/licenses/by/4.0/)**

**Table A 45: PubMed search PNF, Vojta, Bobath**

| **#** | **Concept Block** | **PubMed Search** | **Results 30/10/2023** |
|---|---|---|---|
| **1** | Spinal Cord Injury | "spinal cord injur*" OR "parapleg*" OR "Paraplegia"[MeSH Terms] OR "tetrapleg*" OR "quadripleg*" OR "Quadriplegia"[MeSH Terms] OR "Spinal Cord Injuries"[MeSH Terms] AND ((humans[Filter]) AND (english[Filter] OR german[Filter])) | 65,749 |
| **2** | Interventions | "proprioceptive neuromuscular facilitation*" OR "PNF" OR "bobath*" OR "vojta*" AND (humans[Filter]) AND (english[Filter] OR german[Filter]) | 1,618 |
| | Filter | | |
| 3 | | #1 AND #2 | **24** |

**Table A 46: Embase search PNF, Vojta, Bobath**

| # | Concept Block | Embase Search | Results 30/10/2023 |
|---|---|---|---|
| **1** | Spinal Cord Injury | 'spinal cord injury'/exp OR 'paraplegia'/exp OR 'quadriplegia'/exp OR "spinal cord injur*" OR "parapleg*" OR "tetrapleg*" OR "quadripleg*" AND ('human'/de) AND ([english]/lim OR [german]/lim) | 110,633 |
| **3** | Interventions | "proprioceptive neuromuscular facilitation*" OR "PNF" OR "bobath*" OR "vojta*" AND ('human'/de) AND ([english]/lim OR [german]/lim) | 3,248 |
| | | | |
| | Filter | | |
| **8** | | #1 AND #2 | 48 |

**Table A 47: Cochrane search PNF, Vojta, Bobath**

| # | Concept Block | Cochrane Library Search | Results 30/10/2023 |
|---|---|---|---|
| **1** | Spinal Cord Injury | "spinal cord injur*" OR "parapleg*" OR "tetrapleg*" OR "quadripleg*" | 7 |
| **2** | | MeSH descriptor: [Spinal Cord Injuries] explode all trees | 2,297 |
| **3** | | MeSH descriptor: [Paraplegia] explode all trees | 291 |
| **4** | | MeSH descriptor: [Quadriplegia] explode all trees | 243 |
| **5** | Interventions | "proprioceptive neuromuscular facilitation*" OR "PNF" OR "bobath*" OR "vojta*" | 1,026 |
| | Limits | | |
| **6** | | (#1 OR #2 OR #3 OR #4) AND #5 | 0 |

**Table A 48: PEDro search PNF, Vojta, Bobath**

| **PEDro Search** | |
|---|---|
| Abstract & Title: Spinal cord injury | **19** |
| Therapy: neurodevelopmental therapy, neurofacilitation | |

### A.1.3.3 Coordinationtraining

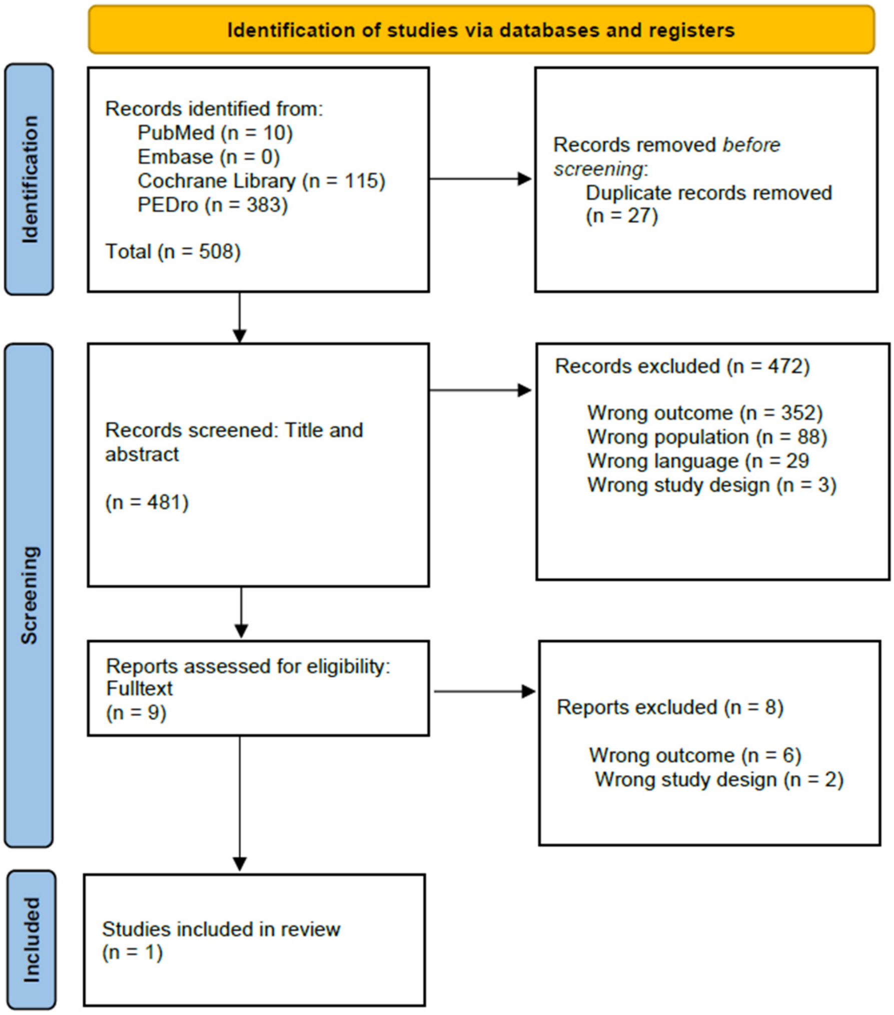


**Figure S15: Flow Chart Coordinationtraining**

**Adapted from Page et al. [1], licensed under CC BY 4.0 (https://creativecommons.org/licenses/by/4.0/)**

**Table A 49: PubMed search Coordinationtraining**

| # | Concept Block | PubMed Search | Results 19/12/2023 |
|---|---|---|---|
| **1** | Spinal Cord Injury | "spinal cord injur*" OR "parapleg*" OR "Paraplegia"[MeSH Terms] OR "tetrapleg*" OR "quadripleg*" OR "Quadriplegia"[MeSH Terms] OR "Spinal Cord Injuries"[MeSH Terms] AND (humans[Filter]) AND (english[Filter] OR german[Filter]) | 66,131 |
| **2** | Interventions | Coordinat* training OR coordinat* education OR coordinat* exercis* OR motor learning AND (humans[Filter]) AND (english[Filter] OR german[Filter]) | 67,270 |
| **3** | Body functions | motor skills* OR fine motor* OR gross motor* OR motor coordination* AND (humans[Filter]) AND (english[Filter] OR german[Filter]) | 47,123 |
| | Filter | Randomized controlled trials | |
| **4** | | #1 AND #2 AND #3 | 144 |
| **5** | | #1 AND #2 AND #3 AND Filter | **10** |

**Tabelle A 50: Embase search Coordinationtraining**

| # | Concept Block | Embase Search | Results 19/12/2023 |
|---|---|---|---|
| **1** | Spinal Cord Injury | 'spinal cord injury'/exp OR 'paraplegia'/exp OR 'quadriplegia'/exp OR "spinal cord injur*" OR "parapleg*" OR "tetrapleg*" OR "quadripleg*" AND ('human'/de) AND ([english]/lim OR [german]/lim) | 114,495 |
| **2** | Interventions | ‘Coordinat* training’ OR ‘coordinat* education’ OR ‘coordinat* exercis*’ OR ‘motor learning’ AND ('human'/de) AND ([english]/lim OR [german]/lim) | 8,617 |
| **3** | Body functions | ‘motor skills*’ OR ‘fine motor*’ OR ‘gross motor*’ OR ‘motor coordination*’ AND ('human'/de) AND ([english]/lim OR [german]/lim) | 47,161 |
| **4** | | #1 AND #2 AND #3 | 20 |
| | Filter | #1 AND #2 AND #3 AND [randomized controlled trial]/lim | **0** |

**Table A 51: Cochrane search Coordinationtraining**

| # | Concept Block | Cochrane Library Search | Results 19/12/2023 |
|---|---|---|---|
| **1** | Spinal Cord Injury | "spinal cord injur*" OR "parapleg*" OR "tetrapleg*" OR "quadripleg*" | 5,350 |
| **2** | | MeSH descriptor: [Spinal Cord Injuries] explode all trees | 2,312 |
| **3** | | MeSH descriptor: [Paraplegia] explode all trees | 294 |
| **4** | | MeSH descriptor: [Quadriplegia] explode all trees | 243 |
| **5** | Interventions | (Coordinat* training OR coordinat* education OR coordinat* exercis* OR motor learning):ti,ab,kw | 9,386 |
| **6** | Body functions | (motor skills* OR fine motor* OR gross motor* OR motor coordination*):ti,ab,kw | 8,625 |
| | Limits | | |
| **7** | | (#1 OR #2 OR #3 OR #4) AND #5 AND #5 | **115** |

**Table A 52: PEDro searcb Coordinationtraining**

| **PEDro Search** | |
|---|---|
| Abstract & Title: Spinal cord injury | **379** |
| Method: Clinical trial | |

### A.1.3.4 Standing training

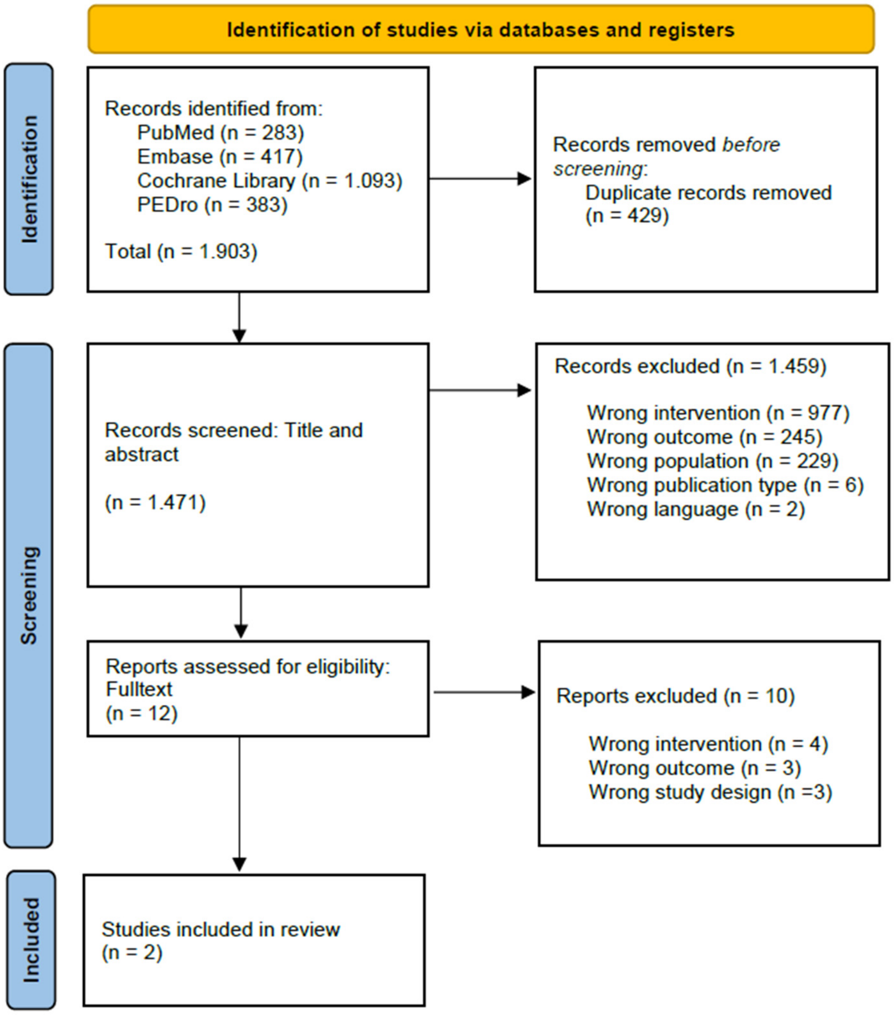


**Figure S16: Flow Chart Standingtraining**

**Adapted from Page et al. [1], licensed under CC BY 4.0 (https://creativecommons.org/licenses/by/4.0/)**

**Table A 53: PubMed search Standingtraining**

| # | Concept Block | PubMed Search | Results 20/12/2023 |
|---|---|---|---|
| **1** | Spinal Cord Injury | "spinal cord injur*" OR "parapleg*" OR "Paraplegia"[MeSH Terms] OR "tetrapleg*" OR "quadripleg*" OR "Quadriplegia"[MeSH Terms] OR "Spinal Cord Injuries"[MeSH Terms] AND (humans[Filter]) AND (english[Filter] OR german[Filter]) | 66,131 |
| **2** | Interventions | standing task OR standing training OR standing exercis* OR standing balance OR prolonged standing OR standing exercis* in parallel bars OR stand* OR postural control OR standing position [MeSH Terms] AND (english[Filter] OR german[Filter]) | 2,408,699 |
| | Filter | Randomized controlled trials | |
| **4** | | #1 AND #2 AND #3 | 6,646 |
| **5** | | #1 AND #2 AND #3 AND Filter | **283** |

**Table A 54: Embase search Standingtraining**

| # | Concept Block | Embase Search | Results 20/12/2023 |
|---|---|---|---|
| **1** | Spinal Cord Injury | 'spinal cord injury'/exp OR 'paraplegia'/exp OR 'quadriplegia'/exp OR "spinal cord injur*" OR "parapleg*" OR "tetrapleg*" OR "quadripleg*" AND ('human'/de) AND ([english]/lim OR [german]/lim) | 114,495 |
| **2** | Interventions | 'standing task' OR 'standing training' OR 'standing exercis*' OR 'standing balance' OR 'prolonged standing' OR 'standing exercis* in parallel bars' OR 'stand*' OR 'postural control' OR 'standing'/exp | 3,071,628 |
| **4** | | #1 AND #2 | 10,554 |
| | Filter | #1 AND #2 | **417** |

**Table A 55: Cochrane search Standingtraining**

| # | Concept Block | Cochrane Library Search | Results 20/12/2023 |
|---|---|---|---|
| **1** | Spinal Cord Injury | "spinal cord injur*" OR "parapleg*" OR "tetrapleg*" OR "quadripleg*" | 5,350 |
| **2** | | MeSH descriptor: [Spinal Cord Injuries] explode all trees | 2,312 |
| **3** | | MeSH descriptor: [Paraplegia] explode all trees | 294 |
| **4** | | MeSH descriptor: [Quadriplegia] explode all trees | 243 |
| **5** | Interventions | (standing task OR standing training OR standing exercis* OR standing balance OR prolonged standing OR standing exercis* in parallel bars OR stand* OR postural control):ti,ab,kw | 288,835 |
| **6** | | MeSH descriptor: [Standing Position] explode all trees | 118 |
| | Limits | Trials | |
| **7** | | (#1 OR #2 OR #3 OR #4) AND (#5 OR #6) AND Limits | **1093** |

**Table A 56: PEDro search Standingtraining**

| **PEDro Search** | |
|---|---|
| Abstract & Title: Spinal cord injury | **379** |
| Method: Clinical trial | |

# Appendix 2: Evidence Tables

**Table A 57: Evidence Table for Assessments**

| Reference<br>Study design | Population (number of participants and characteristics)<br>Included studies (design) | Intervention | Control | Outcome | Results | Quality Assessment |
|---|---|---|---|---|---|---|
| **Abou 2019 [1]**<br>**Systematic Review** | N=887 participants with an age range from 42–62 years<br>* Inclusion: Adults (≥18 years old) with a traumatic or nontraumatic SCI, regardless of the time since injury, level of injury, and completeness of injury.<br>* Exclusion: Studies with mixes neurological populations<br><br>* 10 studies (6 prospective studies, 4 cross-sectional studies)<br>** Amata Chaya 2015; Amatachaya 2018; Jorgensen 2016; Jorgensen 2017]; Jorgensen 2017; Kumprou 2018; Phonthee 2013; Phonthee 2013; Srisim 2015; Wannapakhe 2014* | Clinical balance measures to predict falls | n.a. | Clinical balance measures | **TUG between non-fallers and fallers:** 5 prospective studies<br>Mean difference (95% CI)=–6.65 (–13.12 to –0.17); p=0.04<br>**BBS between non-fallers and fallers:** 2 prospective studies<br>Mean difference (95% CI)=–5.25 (–10.20 to –0.29); p=0.04<br>**5x STS between non-fallers and fallers:** 3 prospective studies<br>Mean difference 95% CI)=1.30 (–0.50 to 3.09); p=0.16<br>**TUG between non-fallers and fallers:** 2 cross-sectional studies<br>Mean difference (95% CI)=6.78 (2.48 to 11.07); p=0.002<br>**TUG between infrequent fallers and recurrent fallers:** 2 cross-sectional studies<br>Mean difference (95% CI)=–0.18 (–2.49 to 2.13); p=0.88<br>**BBS between infrequent fallers and recurrent fallers:** 2 cross-sectional studies<br>Mean difference (95% CI): –3.38 (–8.55 to 1.79); p=0.20 | Critically low |
| **Aigner 2017 [2]**<br>**Case-Control Study** | N=2854 participants with acute SCI<br>* Inclusion: Single event traumatic or ischemic para- or tetraplegia, first EMSCI assessment possible within the first 6 weeks after incidence, patient capable and willing of giving written informed consent, 18 years and older<br>* Exclusion: non-traumatic SCI, pre-known dementia or severe reduction of intelligence, peripheral nerve lesions above the level of lesion, pre-known polyneuropathy, severe craniocerebral injury | 6MWT measurement at each stage: within the first 2 weeks after injury (stage 1), after 4 weeks (stage 2), after 3 months (stage 3), after 6 months (stage 4) and after 12 months (stage 5) | n.a. | 6MWT | **Spearman correlations between 6MWT and S-LEMS**:<br>Stage 1: 0.42, stage 2: 0.43, stage 4: 0.58, stage 4: 0.65, stage 5: 0.65<br>**Spearman correlation between 6MWT and I-LEMS:**<br>Stage 1: 0.70, stage 2: 0.51, stage 3: 0.60, stage 4: 0.68, stage 5: 0.69 | Moderate |

| | | | | | | |
|---|---|---|---|---|---|---|
| **Amatachaya 2014 [3] Cross-sectional Study** | N=94 participants with most of them having a SCI at chronic stage from a non-traumatic lesion* Inclusion: Age >18, able to walk independently with or without a walking device for at least 15 meters (FIM-L 5–7)* Exclusion: Signs and symptoms that might affect walking ability (pain, deformity of the spine and lower extremities, medical complications that limited mobility) | 10MWT | 6MWT | Concurrent validity of the 10MWT | **Correlation between 10MWT and 6MWT**<br>**FIM-L 5:** r**=**0.306; p=0.113<br>**FIM-L 6:** r=0.74; p<0.001<br>**FIM-L 7:**0.83; p<0.001 | Moderate |
| **Akpinar 2017 [4] Cross-Sectional Study** | N=47 participants with an age range from 18–88 years, recruited from the inpatient rehabilitation unit of an education and research hospital. The mean age was 44.19 years. Duration of SCI ranged from 6–197 months. 21 subjects were taking antispastic medication. 25.5% ASIA A, 12.8% ASIA B, 23.4% ASIA C, 38.3% ASIA D with neurological levels of 31.9% cervical, 49% thoracic and 19.1% lumbosacral.<br>* Inclusion: Patients with ASIA A-D who had spasticity and were at least 6 months postinjury; stable drug program (no change in treatment within 30 days, no botulinum injection <90 days)<br>* Exclusion: Multiple central nervous system lesion sites, presence of significant complications that affect spasticity (decubitus ulcer, heterotopic ossification, urinary tract infection, other infections), inability to give informed consent | Spinal Cord Assessment Tools for Spastic Reflexes (SCATS) | n.a. | Reliability of the SCATS | **Interrater agreement on clonus:**<br>κ= 0.854<br>**Test-Retest agreement on clonus:**<br>κ=0.759<br>**Interrater agreement on flexor spasm:**<br>κ=0.669<br>**Test-Retest agreement on flexor spasm:**<br>κ=0.614<br>**Interrater agreement on extensor spasm:**<br>κ=1.000<br>**Test-Retest agreement on extensor spasm:**<br>κ=1.000 | Moderate |

| | | | | | | |
|---|---|---|---|---|---|---|
| **Arora 2018 [5] Systematic Review** | Participants had a level of injury from C1-L5, time since injury between 0.1–48 years and an age between 15–85.7 years* Inclusion: Adult with SCI* Exclusion: n.a. * 127 studies ** not listed here* | Any measure of balance during sitting, standing, walking, or transferring. | n.a. | Balance control or ability to maintain balance | *"* 31 balance measures were identified* Most common used biomechanical measures: Center of Pressure, Center of Mass* Most commonly used balance measure assessing balance during activity: BBS* Most commonly used balance measure assessing sitting balance: mFRT* 20 balance scales are considered to have a high clinical utility score"* | Critically low quality |
| **Arsh 2021 [6] Systematic Review** | N=470 participants<br>* Inclusion: Patients with SCI<br>* Exclusion: Paraplegic patients with causes other than SCI<br><br>* 16 studies<br>** Abou 2020; Adegoke 2002; Boswell-Ruys 2009; Chan 2019; Field-Fote 2010; Jorgensen 2017; Jorgensen 2011; Lemay 2010; Lync 1998; Roy 2021; Sprigle 2003; Sprigle 2007; Srisim 2015; Tamburella 2014; Wadhwa 2016; Wirz 2010* | Validity and/or reliability of any of the clinical instrument used for the assessment of balance function | n.a. | Assessments of balance function | *"* 10 clinical instruments were identified: BBS, mini-BEST, FRT, Functioning in Sitting, T-Shirt Test, Motor Assessment Scale item 3, Sitting Balance Score, 5x STS, Tinetti scale, Sitting Balance measure*<br>** FRT: good-to-excellent test-retest reliability*<br>** BBS: excellent intra-rater reliability, high internal consistency, concurrent validity*<br>** Mini-BEST: excellent test-retest reliability, excellent inter-rater reliability, high internal consistency, good validity*<br>** T-shirt Test: excellent test-retest reliability*<br>** Function in sitting test: excellent test-retest reliability, internal consistency, concurrent validity*<br>** Motor Assessment Scale item 3: good-to-excellent inter-rater reliability, criterion validity*<br>** Sitting Balance Score: good-to-excellent inter-rater reliability, criterion validity*<br>** 5x STS: excellent inter-rater reliability*<br>** Tinetti scale: good-to-excellent intra-rater reliability*<br>** Sitting Balance Measure: high internal consistency, content validity"* | Critically low quality |
| **Arsh 2021 [7] Systematic Review** | N=154 participants with motor-complete and incomplete SCI<br>* Inclusion: Adults with SCI<br>* Exclusion: n.a.<br><br>** 6 studies*<br>** Adegoke 2002; Boswell-Ruys 2009; Field-Fote 2010; Lynch 1998; Sprigle 2003; Sprigle 2007* | Reliability of functional reach test in assessing balance function | n.a. | Functional reach test | *"* Good-to-excellent test-retest reliability of mFRT"* | Critically low quality |

| | | | | | | |
|---|---|---|---|---|---|---|
| **Chan 2019 [8] Cross-Sectional Study** | N=21 participants with chronic motor incomplete SCI and a mean of 7.3 years since injury. 14 patients were female, 7 male with a mean age of 56.9* Inclusion: >18 years of age, traumatic or non-progressive non-traumatic SCI, injury or onset of neurological symptoms occurred <1 year prior, injury rated ASIA C/D, moderate level of trunk control, no condition other than SCI that affected walking or balance ability* Exclusion: Severe contractures or spasticity in the lower extremities that interfered with maintaining an upright posture in standing | Completion of the mini-BESTest at two sessions spaced two weeks apart | n.a. | * mini-BESTest (t1 & t2)* Measures of postural sway during standing (eyes open and eyes closed on a force plate) and lower extremity muscle strength (manual muscle testing) (t2) | **Evaluation of test-retest reliability:**<br>**Anticipatory:** ICC (95% CI)=0.98 (0.95 to 0.99); p<0.01<br>**Reactive:** ICC (95% CI)=0.94 (0.84 to 0.97); p<0.01<br>**Sensory:** ICC (95% CI)=0.95 (0.87 to 0.98); p<0.01<br>**Dynamic:** ICC (95% CI)=0.97 (0.93 to 0.99); p<0.01<br>**Total:** ICC (95% CI)=0.98 (0.95 to 0.99); p<0.01 | Moderate |
| | | | | | **Concurrent validity:**<br>**Pearson's r between mini-BESTest and center of pressure velocity in anterior posterior (eyes open):**<br>Total (r)=–0.56, p=0.01; sensory (r): –0.48, p=0.03<br>**Pearson's r between mini-BESTest and center of pressure velocity in medial-lateral (eyes open):**<br>Total (r)=–0.71, p<0.01; sensory (r): –0.64, p<0.01<br>**Pearson's r between mini-BESTest and center of pressure velocity in anterior posterior (eyes closed):**<br>Total (r)=–0.04, p=0.86; sensory (r): –0.23, p=0.36<br>**Pearson's r between mini-BESTest and center of pressure velocity in medial-lateral (eyes-closed):**<br>Total (r)=0.07, p=0.78; sensory (r): –0.10, p=0.70 | |
| | | | | | **Convergent validity:**<br>**Correlation lower extremity strength with mini-BESTest total scores:**<br>r=0.73, p<0.001 | |

Attachment to: Irrgang S, Himmelhaus S, Allek K, Bartholet C, Bersch-Porada I, Curt A, Huber B, Kuhn D, Kynast K, Weidner N, Scheel-Sailer A. Rehabilitation of the lower extremities, standing and walking function in people with spinal cord injury or disease: Guideline of the German-Speaking Medical Society for Spinal Cord Injury. GMS Ger Med Sci. 2025;23:Doc02. DOI: 10.3205/000338, URN: urn:nbn:de:0183-0003383

| | | | | | | |
|---|---|---|---|---|---|---|
| **Jorgensen 2017 [9] Cross-Sectional Study** | N=46 participants of which 74% were able to walk 10m without walking aid and 48% classified themselves as community walkers not using walking aids. 70% were male, the mean age was 54.5 years, the median time since injuries were 6.5 years and 85% had AIS D.<br>* Inclusion: Traumatic SCI, ASIA A-D at least one year post injury and at least 18 years of age, able to walk 10m independently with or without walking aids<br>* Exclusion: Motor complete injuries above C5 and lesions below L5 | BBS, Mini BESTest | SCIM III, TUG, 10MWT, WISI II, The International Spinal Cord Injury Quality of Life Basic Dataset | Validity of the BBS | *"* Ceiling effect in the BBS (28% score the maximum score)<br>* Good overall internal consistency (α=0.94)"*<br><br>**Convergent validity:**<br>**Mini BESTest and BBS:** r=0.899; p<0.001<br>**Mini BESTest and TUG**: r=–0.75; p<0.001<br>**Mini BESTest and SCIM:** r=0.88; p<0.001<br>**Mini BESTest and 10MWT**: r=–0.88; p<0.001<br>**Mini BESTest and WISCI II:** r=0.63; p<0.001<br>**Mini BESTest and FES-I:** r=–0.62; p<0.001<br>**Mini BESTest and fear of falling:** r=–0.32; p=0.83<br><br>**Divergent validity:**<br>**Mini BESTest and general QOL question:** r=0.19; p=0.20<br><br>**Known-groups validity:**<br>*"*Ability to discriminate between community walkers with and without walking aids (p<0.001)<br>* Cut-off points: 47/56 points<br>* No discrimination between infrequent/recurrent fallers (p=0.78)"* | Moderate |
| **Lemay 2010 [10] Cross-Sectional Study** | N=32 participants (7 women, 25 men) with mean age of 47.9 years, mean time post lesion of 77.2 days and more tetraplegic participants (17/15).* Inclusion: Adults with ASIA D either of traumatic or nontraumatic etiology and the ability to walk 10m independently with or without upper-extremity assistive devices* Exclusion: Other concomitant neurological conditions in addition to the SCI or who had walking difficulties before the SCI | BBS | SCI-FAI, WISCI II, 10MWT, TUG | Validity of the BBS | **BBS - SCI-FAI parameter:** Spearman's r=0.747<br>**BBS - SCI-FAI assistive devices:** Spearman's r=0.714<br>**BBS - SCI-FAI mobility:** Spearman's r=0.740<br>**BBS - 2MWT:** Spearman's r=0.781<br>**BBS - WISCI II:** Spearman's r=0.816<br>**BBS - 10MWT:** Spearman's r=0.792<br>**BBS - TUG:** Spearman's r=–0.815<br><br>*"* Paraplegia: walker users had scores below 30/56<br>* Tetraplegia: walker users had a broad range of scores (31–55/56)"* | Moderate |

| | | | | | | |
|---|---|---|---|---|---|---|
| **Mills 2018 [11] Cross-Sectional Study** | N=61 participants with an age range from 19–65 years (median: 35.5 years). Time since injury was 1.1–30.3 years (median: 7 years).<br>* Inclusion: >1 year duration of traumatic SCI, 18–65 years, currently experiencing spasticity on history, history of stable spasticity over the past 2 weeks, no anticipated change in spasticity treatment during study enrolment<br>* Exclusion: not English speaking, not able to provide informed consent (cognitive disorders, e.g. traumatic brain injury) | Administration of the Penn Spasm Frequency Scale (PSFS) over phone | n.a. | PSFS | **Intra-rater reliability:**<br>**5-level spasm frequency:**<br>T1 vs T2: κ (95% CI)=0.822 (0.709 to 0.935)<br>**5-level spasm frequency:**<br>T1 vs T4: κ (95% CI)=0.734 (0.586 to 0.883)<br>**13-level spasm frequency-severity combination**:<br>T1 vs T2: κ (95% CI)=0.812 (0.705 to 0.919)<br>**13-level spasm frequency-severity combination:**<br>T1 vs T4: κ (95% CI)=0.729 (0.586 to 0.872)<br>**Inter-rater reliability:**<br>**5-level spasm frequency:**<br>T2 vs T3: κ (95% CI)=0.862 (0.759 to 0.965)<br>**13-level spasm frequency-severity combination:**<br>T2 vs T3: κ (95% CI)=0.857 (0.762–0.952) | Adequate |
| **Poncumhak 2013 [12] Cross-Sectional Study** | N=66 participants with mean age of 50.9 years, more male than females and more non-traumatic causes*<br>Inclusion: Ability to stand up from a chair or bed independently without use of hands and to walk independently with or without a walking device for at least 50m continuously (FIM-L score 6–7)*<br>Exclusion: n.a | TUG | FIM-L scores | TUG | **Inter-test reliability TUG and FIM-L 6:** ICCs (95% CI)=0.999 (0.999 to 1.000)<br>**Inter-test reliability TUG and FIM-L 7:** ICCs (95% CI)=1.000 (0.999 to 1.000)<br>**Correlation between TUG and FIM-L scores:** r=–0.692 | Moderate |
| **Roy 2021 [13] Cohort Study** | N=23 participants with an injury level between C2 and L5. The mean age was 55.2 years, the time post-surgery 49.3 days. More male than female (17/6) were included, 13 tetraplegic and 10 paraplegic participants with more ASIA D (B/D: 1/22)<br>* Inclusion: undergone surgery, age between 18 and 75 years old, traumatic or non-traumatic SCI, complete or incomplete SCI, able to stand without aid for 30 seconds, spoke French or English, tolerated 20 minutes of evaluation with rest periods, able to provide an informed consent<br>* Exclusion: severe neurological condition other than SCI, musculo-skeletal or medical condition that would interfere with the measurements, psychiatric condition or dementia | mini-BESTest at baseline for half the participants and a few days before discharge for the other half | n.a. | mini-BESTest | **Test-retest reliability overall:**<br>ICC (95% CI)=0.94 (0.87 to 0.97)<br>**Inter-rater reliability overall:**<br>ICC (95% CI)=0.96 (0.91 to 0.98)<br>**Minimal detectable change:**<br>3.43–3.82 points | Adequate |

| | | | | | | |
|---|---|---|---|---|---|---|
| **Tomaschek 2019 [14] Systematic Review** | N=4929 participants from the acute and subacute phase<br>* Inclusion: Patients with acute and subacute SCI during initial rehabilitation<br>* Exclusion: Patients with different health conditions or in the chronic phase of SCI<br><br>* 33 studies (19 validation studies, 1 systematic review, 13 observational studies)<br>** Aigner 2016; Akpinar 2017; Akpinar 2017; Anton 2017; Bergamaschi 2014; Chan 2017; Delparte 2016; Ditunno 2013; Eaton 2018; Fekete 2014; Freund 2013; Gagnon 2016; Glennie 2014; Jette 2015; Kalsi-Ryan 2014; Kalsi-Ryan 2016; Klyce 2015; Krause 2015; Krishnan 2016; Marino 2016; Marino 2018; Misirlioglu 2016; Noijen 2015; Rognoni 2014; Scivoletto 2014; Street 2015; Tate 2013; Unalan 2015; van Diemen 2017; Velstra 2014; Velstra 2015; Walden 2016; Zanca 2013* | Assessments for all functioning aspects of patients with an acute or subacute SCI during initial rehabilitation | No comparator | Psychometric properties, Recommendations concerning the use of outcome measures and assessments | *"* 29 assessments were identified<br>* Body functions: SAVES<br>* Mental functions: ADAPSS-SF, Body Experience Questionnaire, FSS, Interview for Prolonged Grief Disorder<br>* Sensory functions & pain: MAS, TAS, self-reported pain locations, self-reported rating of pain intensity<br>* Neuro-musculoskeletal function: ISNCSCI, MRI, S3 Pressure Sensation, SCATS<br>* Activity: FIM, DHI, GRASSP, SCI-FI/AT, SCIM III-RS, SCIM-III, WISCI<br>* Participation: WORQ-SELF<br>* Mobility: CB&M, CUE-T, CUE-Q, Propulsion tests (slalom, 6m, 20m)<br>* QOL: Illness Perception Questionnaire, SF–36<br>* Functions of the skin & related structures: SCIPUS"* | Critically low quality |
| **van Hedel 2005 [15] Cross-Sectional Study** | N=75 participants with a mean age of 54 years, more males (60%) and more AISA D classes (81%)<br>* Inclusion: Able to perform most of the walking tests<br>* Exclusion: Additional gait-impairing deficits | TUG, 10MWT, 6MWT | WISCI II | TUG | **Correlation between WISCI II and TUG:**<br>r=–0.76<br>**Correlation between TUG and 10MWT:**<br>r=0.89<br>**Correlation between TUG and 6MWT:**<br>r=–0.88 | Moderate |
| **Willi 2022 [16] Cross-Sectional Study** | N=50 participants with a mean age of 52.6 years and more male participants (33/17). ASIA D was most represented (41) and more traumatic injuries were included (28).<br>* Inclusion: acute or chronic SCI, >18 years and a minimum walking speed of 0.17 m/s, ability to walk without physical assistance<br>* Exclusion: current orthopedic problems, major psychosis or depression, history of severe heart condition | * 2 MWT<br>* Protocol: 2 test days (separated by 1–7 days). Day 1: Familiarization run of the 2MWT, break of 30min, performance of 2MWT and 10MWT in randomized order, break of at least 30min, performance of WISCI II. Day 2: 2MWT and 6MWT performed in a randomized order and separated by a 30min break | n.a. | * 2MWT<br>* Secondary measures: 6MWT, 10MWT, WISCI II | **Construct validity:**<br>**2MWT - 6MWT:**<br>r (95% CI)=0.992 (0.986 to 0.996)<br>**2MWT - Self 10MWT:**<br>r (95% CI)=0.964 (0.941 to 0.986)<br>**2MWT - Max 10MWT**:<br>r (95% CI)=0.974 (0.956 to 0.988)<br>**2MWT - WISCI II:**<br>r (95% CI)=0.571 (0.356 to 0.784)<br>**Test-retest reliability:2MWT (T1 vs T2):** ICC: 0.980; p<0.001<br>**SE of measurement 2MWT overall:** 7.5m<br>**MDC 2MWT overall:** 20.9m | High |

**Table A 58: Evidence Table for Interventions**

| Reference<br>**Study design** | Population (number of participants and characteristics)<br>**Included studies (design)** | Intervention | Control | Outcome | Results | Level of evidence |
|---|---|---|---|---|---|---|
| **Amatachaya 2019 [17]<br>Randomized cross-over trial** | N=22 participants with an average age of 49.81 years and a post-injury time of 60.86 months. A large proportion (95%) had incomplete paraplegia and a mild lesion severity (AIS D, 77%). 55% of the participants were able to walk without a walking device.<br>* Inclusion: age over 18 years, a BMI between 18.50 and 29.90 kg/m², AIS A/D from trauma or nonprogressive disease, post-injury time of at least 12 months, walking independently for over 17 meters with or without assistive devices, ability to read Thai<br>* Exclusion: Not able to complete training program, condition that might affect the participation (e.g. pain, deformity of joints, color blindness) | * Dual-task obstacle crossing training<br>* Four obstacles of varying sizes were randomly placed along a 10-meter walkway at 2-meter intervals<br>* Navigation over the obstacles using their legs and walking devices (if any) as continuously as possible<br>* Simultaneously performing a Stroop Color and Word Test task<br>* Training program lasted 30 minutes, excluding resting periods | * Single-task obstacle crossing training<br>* Four obstacles of varying sizes were randomly placed along a 10-meter walkway at 2-meter intervals<br>* Navigation over the obstacles using their legs and walking devices (if any) as continu-ously as possible<br>* Training program lasted 30 minutes, excluding resting periods | * 10 MWT<br>* Percent of the Stroop Color and Word Test task errors<br>* TUG<br>* 5x STS | **Single-task 10MWT:**<br>IG (95% CI)=0.03±0.05 (0.01 to 0.06), CG (95% CI)=0.04±0.05 ( 0.01 to 0.06); p=0.66<br>**Dual-task 10WMT:**<br>IG (95% CI)=0.05±0.06 (0.02 to 0.08), CG (95% CI)=0.06±0.07 (0.02 to 0.09); p=0.73<br>**Percent of Stroop Color and Word Test task errors:**<br>IG (95% CI)=–8.19±10.90 (–13.02 to –3.36), CG (95% CI)=–2.45±9.06 (–6.47 to 1.56); p=0.014<br>**TUG:**<br>IG (95% CI)=2.40±2.92 (1.11 to 3.69), CG (95% CI)=1.70±2.63 (0.54 to 2.87); p=0.06<br>**FTSTS:**<br>IG (95% CI)=1.79±1.57 (1.10 to 2.49), CG (95% CI)=1.33±1.25 (0.77 to 1.89); p=0.53 | Some concerns for risk of bias |
| **Adams 2011 [18]<br>Randomized cross-over trial** | N=7 participants (6 male, 1 female) with mean time since injury of 5 years and motor complete or incomplete paraplegia or tetraplegia<br>* Inclusion: Chronic (>1 year) complete or incomplete paraplegia or tetraplegia, self-reported presence of stable spasticity, consistent medication, physical activity/physiotherapy routines during the previous 6 months, reliance on a wheelchair as a primary mode of mobility<br>* Exclusion: Participation in BWSTT during the previous 6 months, medical contraindications to the performance of BWSTT or TTS | * 12 sessions of TTS over 4 weeks<br>* One session was at least 45 minutes | * 12 sessions of BWSTT over 4 weeks<br>* One session was at least 45 minutes | * MAS<br>* SCATS<br>* SCI-SET<br>* PSFS<br>* FIM Motor subscore | *"** Effect after one session:<br>Decrease in extensor spasm following TTS (ES pre/post=0.68)<br>* Greater reduction in passive resistance to movement (ES change score=0.69) and flexor spasm (ES change score=0.57) in BWSTT group<br>** Effect after 4 weeks:<br>* Reduction of extensor spasm following TTS (ES pre/post=0.95)<br>* Greater reduction of extensor spasm following TTS in comparison to BWSTT ( ES change score=1.32)<br>* Greater reduction of flexor spasm in BWSTT than in TTS (ES change score=0.79)<br>* More improvement in clonus after BWSTT compared to TTS (ES change score=0.66)<br>* No results in group changes of the SCI-SET or the PSFS<br>* More positive changes in quality of life (ES change score=0.50) and FIM Motor subscore (ES change score=1.24) in BWSTT"* | Some concerns for risk of bias |

| | | | | | | |
|---|---|---|---|---|---|---|
| **Ben 2005 [19] Randomized controlled trial** | N=20 participants with a mean time since injury of 4 months, mean age of 34 years and more male participants (n=16)<br>* Inclusion: SCI within the last 12 months and admitted to one of two SCI units in Sidney, commencing sitting out of bed, less than grade 2/5 strength in the lower limbs<br>* Exclusion: History of trauma to the pelvis or legs, unable to tolerate standing, likelihood to be discharged from hospital within three months, unlikely to co-operate | * Weight-bearing and stretched leg<br>* Standing in a tilt-table for 30 minutes, three times per week for 12 weeks<br>* A wedge inclined 15 degrees was placed on a high block and positioned under the intervention foot ensuring a dorsiflexed position with body weight borne solely through this leg | * Non-weight-bearing and non-stretched leg<br>* Standing in a tilt-table for 30 minutes, three times per week for 12 weeks<br>* Control foot was left in an unsupported plantarflexed position with no body weight borne through it | * Ankle mobility: passive ankle dorsiflex measured with a specified device | **Overall treatment effect in ankle mobility (degrees):**<br>Mean (95% CI)=4 (2 to 6) | Some concerns for risk of bias |
| **Ellappen 2018 [20] Systematic Review** | N=142 participants with participant age varying from 5 to 70 years<br>* Inclusion: People with SCI<br>* Exclusion: n.a.<br><br>* 15 studies (4 systematic reviews, 7 non-*RCTs, 2 RCTs, 2 case studies)*<br>** Becker 2009; Gass 2001; Gass 2002; Jung 2014; Kesiktas 2004; Li 2017; Lucksch 2013; Prosser 2007; Recio 2017; Stevens 2014; Stevens 2015; Tamburella 2013; Tweedy 2016; Wall 2017; Zamparo 1998* | Hydrotherapy | n.a. | * Hydrotherapy interventions for PWSCI<br>* The impact of hydrotherapy on PWSCI gait kinematics | *"* Kinematic gait analysis: Patient's gait kinematics, walking speed and stride length improved after completion of hydrotherapy*<br>** Decreased spasticity: Reduced muscle spasticity with reduced dosage of oral baclofen due to hydrotherapy [2]*<br>** Cardiorespiratory benefits: Greater energy expenditure at slower speed and prolonged activity and enhanced cardiorespiratory function." [7]* | Critically low quality |

| | | | | | | |
|---|---|---|---|---|---|---|
| **Fang 2021 [21] Systematic Review** | N=177 participants (99 subject for the quantitative analysis, 78 patients for the qualitative analysis). Age ranged from 20–67 years, time since injury was below 4 weeks to 17 years. ASIA level was from A-D, with most subjects with ASIA A<br>* Inclusion: Adults with SCI, regardless of traumatic or non-traumatic injury, the time since injury, humans.<br>and gender<br>* Exclusion: n.a.<br><br>** 12 studies (1 RCT, 11 non-RCTs)*<br>** Fattal 2018; Krause 1008; Kuhn 2014; Mazzoleni 2013; Mazzoleni 2017; Popovic-Maneski 2018; Ralston 2013; Reichenfelser 2012; Sadowsky 2013; Skéld 2002; Szecsi 2009; Yasae 2015* | Functional electrical stimulation combined with active cycling training for lower limbs | Control group or no intervention | * Primary outcome: spasticity (MAS)<br>* Secondary outcome: walking abilities (6MWT, TUG, LEMS) | **Spasticity after FES-cycling training:** 2 prospective studies, 2 single group studies, 1 RCT, 1 two group study<br>Difference in means (95% CI)=–0.860 (–1.538 to –0.182); p=0.013<br>**Spasticity after one session of FES-cycling:** 1 cross-over study, 1 single group study<br>Difference in means (95% CI)=–1.067 (–1.270 to –0.865); p=0.000<br>**Subgroup analysis for spasticity:** 2 single group studies, 2 prospective studies, 1 RCT, 1 two group study<br><20 sessions: Difference in means (95% CI)=–0.384 (–0.580 to –0.189); p=0.000<br>>20 sessions: Difference in means (95% CI)=–1.336 (–1.448 to –1.223); p=0.000<br>overall: Difference in means (95% CI)=–1.100 (–1.198 to –1.003); p=0.000<br>**6MWT after FES-cycling training:** 1 prospective study, 1 single group study<br>Difference in means (95% CI)=12.335 (7.690–16.981); p=0.000<br>**TUG after FES-cycling training:** 1 prospective study, 1 single group study<br>Difference in means (95% CI)=–31.944 (–51.040 to –12.949); p=0.001<br>**LEMS after FES-cycling training:** 1 prospective study, 1 two group study<br>Difference in means (95% CI)=4.650 (1.308 to 7.991); p=0.006 | High quality |

| | | | | | | |
|---|---|---|---|---|---|---|
| **Gaspar 2019 [22] Systematic Review** | N=631 participants with heterogenous age, type/level of injury and time since injury<br>* Inclusion: Subjects with SCI<br>* Exclusion: n.a.<br><br>** 25 studies*<br>** Benito-Penalva 2012; Bizzarini 2005; Boswell-Ruys 2010; Brurok 2011; Fleerkotte 2014; Giangregorio 2012; Ginis 2003; Harvey 2010; Hicks 2003; Jayaraman 2013; Kapadia 2014; Labrueyere 2014; Lam 2015; Mirbagheri 2015; Nooijen 2009; Postans 2004; Rosety-Rodriguez 2014; Sayenko 2010; Schwartz 2011; Sharif 2014; Silva 1998; Tawashy 2010; Totosy 2015; Trasher 2006; Vasiliadis 2014* | Aerobic exercises, resistance training, balance training, gait training | n.a. | Based on the ICF framework as well as physiological parameters for exercise prescription. | *"* Aerobic exercise had main observed outcomes in improved endurance of ventilatory musculature, increase in cardiorespiratory fitness and influenced sex hormone levels*<br>** Resistance training increased the isometric and dynamic muscle strength of the lower limbs in participants with incomplete SCI*<br>** No clear results for balance training due to low number of included studies*<br>** Gait training was performed in association with other devices (e.g. FES). Interventions resulted in increased speed, endurance, and gait control*<br>** Overall, further observed outcomes were benefits in QOL, stress reduction, decrease in perception of pain and depression, increase in physical self-concept"* | Low Quality |
| **Ibitoye 2019 [23] Systematic Review** | N=95 participants<br>* Inclusion: Participants with low-cervical or thoracic spinal cord lesions<br>* Exclusion: Lesion levels above C4<br><br>* 25 studies<br>* Abbas 1991; Audu 2017; Bajd 1999; Braz 2009; Davis 1997; Davis 1999; Davis 2001; Erwins 1988; Fisher 2008; Fujita 1995; Gollee 2004; Harkema 2011; Hunt 2001; Jaeger 1989; Jaime 2002; Kobravi 2012; Kralj 1986; Mihelj 1997; Nataraj 2013; Rejc 2015; Rejc 2017; Triolo 2012; Ulhir 2000 | Standing task with a duration of standing greater than 20min supported by FES | n.a. | Duration of standing, Dexterity/degree of freedom, level of arm engagement | *"* Limited success for the maintenance of upright stance and posture (short duration and poor quality of standing)*<br>** Different closed-loop FES-supported strategies, but none of them is widely accepted*<br>** FES-supported standing is most effective in certain SCI-populations (low tetraplegia, thoracic level paraplegia), but it requires adequate muscle force or power"* | Critically low quality |

| | | | | | | |
|---|---|---|---|---|---|---|
| **Lawrason 2020 [24] Systematic Review** | N=531 participants with an average age of 46.31 years and an average time since injury of 9.62 years<br>* Inclusion: Adults with SCI (traumatic/non-traumatic) who ambulate as their primary mode of mobility. Gaid aids were allowed<br>* Exclusion: Individuals with a neurological condition that is not a SCI. Individuals with a SCI who use a wheelchair as their primary mode of mobility<br><br>* 17 studies (1 RCT, 4 Pre-post studies, 11 cross-sectional studies, 1 mixed-methods study)<br>** Blauwet 2013; Bowditch 2015; DiPiro 2015; DiPiro 2016; Freixes 2012; Garshick 2016; Leech 2017; Ginis 2010; Ginis 2017; Rauch 2017; Rauch 2016; Roberton 2011; Rosly 2018; Scelza 2005; Stapleton 2014; Wouda 2016* | All types of physical activity | n.a. | * Amount and types of physical activity performed by SCI ambulators<br>* Correlates and outcomes of physical activity among SCI ambulators | *"* Leisure-time physical activity: in average 3h/week light LTPA, 2h/week moderate LTPA, 1h/week strenuous LTPA and 1h/week muscle-strengthening exercises<br>* Strength-related benefits were reported from a 12-week resistance training program (muscle strength, cardiovascular capacity, VO2 peak, steps taken outside training)<br>* Decrease in pain and fatigue outcomes after an aerobic exercise program (25% decrease in pain, 46% decrease in pain interference, 20% decrease in fatigue)<br>* Decrease in depression (60%) after an aerobic exercise program"* | Critically low quality |
| **Li 2017 [25] Systematic Review** | N=146 participants with SCI. More male were reported than female with an age between 18–63 years. The time since injury was from 7 months to 28 years<br>* Inclusion: Patients with acute or chronic, complete, or incomplete SCI<br>* Exclusion: Mixed sample without data specific on participants with SCI<br><br>* 8 studies<br>** Broach 1997; da Sivla 2005; Jung 2014; Kesiktas 2004; Pachalski 1980; Stevens 2015: Stevens 2015; Stowell 2001* | Aquatic exercises (e.g. swimming, underwater treadmill training) | n.a. | Physical function (e.g. functional independence, muscle contraction, mobility, walking ability) | *"* Physical function: Functional independence and walking ability was improved with aquatic exercises"* | Low quality |

| | | | | | | |
|---|---|---|---|---|---|---|
| **Mehrholz 2017 [26] Systematic Review** | N=586 participants with ASIA A-D and injury level C1-L4. Mean age was 37 years<br>* Inclusion: Participants of any age and gender and any type of traumatic SCI regardless of time since injury or level of initial walking ability<br>* Exclusion: n.a.<br><br>* 113 studies (13 RCTs)<br>* *Alexeeva 2011; Dobkin 2006; Esclarin-Ruz 2014; Field-Fote 2011; Hornby 20007; Kapadia 2014; Labruyere 2014; Lucareli 2011; Postans 2004; Sadeghi 2015; Senthilvelkumar 2015; Shin 2014; Yang 2014* | Body-weight-supported treadmill training (includes treadmill gait training with body-weight-support, manual guidance, FES, or some additional overground training), robotic assisted gait training (included gait training with automated electromechanical devices or robotic devices with or without body-weight-support, manual guidance, FES, or additional overground training) | Overground gait training and other forms of physiotherapy (gait training that involved moving across the floor with or without body-weight support. FES, orthoses, aids, or manual assistance) | * Walking speed (10MWT /15MWT) and walking distance (6MWT, 2MWT)<br>* Adverse events | **BWSTT vs overground gait training and other forms of physiotherapy on walking speed:**<br>9 RCTs<br>Mean difference (95% CI)=–0.03 (–0.10 to 0.04); p=0.35<br>**BWSTT vs overground gait training and other forms of physiotherapy on walking distance:**<br>8 RCTs<br>Mean difference (95% CI)=–6.77 (–44.74 to 31.20); p=0.73<br>**Robotic-assisted gait training vs overground gait training and other forms of physiotherapy on walking speed:** 3 RCTs<br>Mean difference (95% CI)=–0.04 (–0.21 to 0.13); p=0.66<br>**Robotic-assisted gait training vs overground gait training and other forms of physiotherapy on walking distance:** 3 RCTs<br>Mean difference (95% CI)=–6.14 (–85.92 to 73.63); p=0.88 | Critically low quality |
| **Quel de Oliviera 2016 [27] Systematic Review** | N=639 participants with mean age from 28.6–59.0 years and mean time since injury from 4.5 weeks to 9.5 years. 26% with ASIA A, 58% with ASIA C/D<br>* Inclusion: Acute and chronic traumatic SCI with ASIA A-D<br>* Exclusion: n.a.<br><br>* 19 studies (16 RCTs, 3 CTs)<br>* *Adams 2011; Alcobendas-Maestro 2012; Alexeeva 2011; Beekhuizen 2008; Dobkin 2006; Esclarin-Ruz 2014; Harness 2008; Harvey 2016; Hitzig 2013; Jones 2014; Kapadia 2013; Kapadia 2014; Labruyere 2014; Nasser 2014; Niu 2014; Popovic 2006; Popovic 2011; Schwartz 2011; Yang 2014* | Activity-based therapy | Conventional rehabilitation (task-specific training or electrical stimulation targeting the areas above the site of injury), aerobic training, progressive resistance training, training of ADLs, no intervention) | * General mobility (BBS, WISCI, Jebsen-Taylor Hand Function Test)<br>* Functional ability (FIM, SCIM)<br>* QOL (SF–36, Quality of Life Index for SCI, Satisfaction with Life Scale) | **Activity-based interventions vs no intervention for lower limb mobility:** 2 RCTs<br>Mean difference (95%CI)=0.2362 (–0.2076 to 0.681)<br>**Activity-based interventions for lower limbs vs conventional physical intervention for independence:** 8 RCTs, 1 CT<br>Mean difference (95% CI)=0.1266 (–0.139 to 0.3927)<br>**Activity-based interventions for lower limbs vs conventional physical intervention for mobility:** 8 RCTs, 2 CTs<br>Mean difference (95% CI)=0.2051 (–0.0859 to 0.4962)<br>**Activity-based interventions vs conventional physical intervention for QOL:** 4 RCTs<br>Mean difference (95% CI)=0.1146 (–0.225 to 0.4543) | Low quality |

| | | | | | | |
|---|---|---|---|---|---|---|
| **Stampacchia 2022 [28] Systematic Review** | N=1487 participants with a median age of 46–47 years, more male participants and more chronic lesions<br>* Inclusion: Persons with SCI, no specific criteria related to injury type, injury onset or injury severity<br>* Exclusion: n.a.<br><br>* 62 studies (18 RCTs, 4 quasi-RCTs, 20 observational studies, 5 case series)<br>** Alobendas-Maestro 2012, Asselin 2015, Asselin 2021; Baunsgaard 2018; Birch 2017; Brinkemper 2021; Cheung 2019; Choi 2020; Corbianco 2021; Escalona 2018; Escalona 2020; Esclarin-Ruz 2014; Esquenazi 2012; Evans 2015; Evans 2021; Faulker 2021; Field-Fote 2005; Field-Fote 2011; Gagnon 2017; Gagnon 2018; Gorgey 2017; Gorman 2016; Hartigan 2015; Hong 2020; Hornby 2005; Jansen 2017; Jansen 2017; Kerdraon 2021; Kim 2021; Knezevic 2021; Koljonen 2021; Kressler 2019; Kwohn 2020; Labruyere 2014; Lam 2005; Maher 2020; Martinez 2019; Mazzoleni 2017; McIntosh 2020; Meijneke 2021; Mirbagheri 2015; Mirbagheri 2015; Nandor 2021; Niu 2014; Noijen-Hoeve 2009; Okawara 2020; Park 2021; Piira 2019; Shapkova 2016; Shin 2014; Simis 2012; Stampacchia 2020; Swank 2020; Talaty 2013; Tefertiller 2017; Tsai 2020; van Dijsseldonk 2017; Wu 2016; Xiang 2020; Yang 2015; Yatsuya 2018* | Body weight supported treadmill-based robotic training, overground robotic exoskeleton training | n.a. | Walking outcomes | *"* Total session: BWSTRT: 31, ORET: 20<br>* Duration of intervention (weeks): BWSTRT: 8, ORET: 8<br>* Sessions frequency (sessions/week): BWSTRT: 3, ORET: 3<br>* Duration of one session: BWSTRT: 45, ORET: 60<br>* Increased walking speed (10MWT) & improved endurance (6MWT) after training with BWSTRT<br>* Walking speed, endurance and TUG improved after ORET<br>* No conclusive results on improvements in FIM/SCIM"* | Low quality |

| | | | | | | |
|---|---|---|---|---|---|---|
| **van der Scheer 2021 [29] Systematic Review** | N=999 participants with a mean age of 36 years, mean time since injury of 9.0 years and more men than women (782/143). The averaged lesion level was from C6-T8 with most participants with AISA A.<br>* Inclusion: Sample with at least 50% with adults (>16 years) with traumatic or non-traumatic SCI (any time post-onset SCI) who were eligible and responsive to FES cycling<br>* Exclusion: Congenital condition (e.g. spina bifida) or progressive disease (e.g. MS with spinal cord involvement)<br><br>* 97 studies<br>** not listed here* | * FES lower body cycling exercise intervention (defined as a modality whereby transcutaneous electrical currents are applied to paralyzed or paretic muscles)<br>* Describing exercise prescription parameters (period, frequency, duration) | * Not receiving FES cycling exercise interventions<br>* Receiving usual care<br>* Comparison of two FES cycling interventions | Any type of health or fitness-related outcome measures | *"** Significant improvements in the outcome categories:*<br>** Muscle health in 30 out of 36 studies*<br>** Power output in 34 out of 35 studies*<br>** Aerobic fitness in 20 out of 26 studies*<br>** Bone health in 11 out of 23 studies*<br>** Cardiovascular and metabolic factors in 16 out of 21 studies*<br>** Fat mass in 8 out of 16 studies*<br>** Muscle strength in 12 out of 14 studies*<br>** Other secondary health conditions in 7 out of 13 studies*<br>** Subjective well-being in 7 out of 10 studies*<br>** Functional and neurological outcomes in 3 out of 5 studies*<br>*** Exercise prescription:*<br>** Period: 16 weeks*<br>** Frequency: 3 times/week*<br>** Duration: 30 min/session*<br>** Min cycle speed: 35 RPM / Max cycle speed: 50 RPM*<br>** Puls width: 300 µs*<br>** Amplitude: 140 MA*<br>** Stimulation frequency: 35 Hz*<br>*** Adverse events:*<br>** 18 participants experienced suspected adverse events*<br>** 1 out of 18 participants experienced a serious adverse event (development of haemotoma in the ischial region)*<br>** 17 out of 18 participants experiences other suspected adverse reactions (post-exercise hypotension, increased spasticity, light-headedness, skin redness, bowl accident, autonomic dysreflexia, increased leg swelling, small quadriceps haematoma)"* | Moderate quality |

## References

### Appendix 1

1. Page MJ, McKenzie JE, Bossuyt PM, Boutron I, Hoffmann TC, Mulrow CD, Shamseer L, Tetzlaff JM, Akl EA, Brennan SE, Chou R, Glanville J, Grimshaw JM, Hróbjartsson A, Lalu MM, Li T, Loder EW, Mayo-Wilson E, McDonald S, McGuinness LA, Stewart LA, Thomas J, Tricco AC, Welch VA, Whiting P, Moher D. The PRISMA 2020 statement: an updated guideline for reporting systematic reviews. BMJ. 2021 Mar 29;372:n71. DOI: 10.1136/bmj.n71

### Appendix 2

1. Abou L, Ilha J, Romanini F, Rice LA. Do clinical balance measures have the ability to predict falls among ambulatory individuals with spinal cord injury? A systematic review and meta-analysis. Spinal Cord. 2019 Dec;57(12):1001-13. DOI: 10.1038/s41393-019-0346-5
2. Aigner A, Curt A, Tanadini LG, Maathuis MH. Concurrent validity of single and groups of walking assessments following acute spinal cord injury. Spinal Cord. 2017 May;55(5):435-40. DOI: 10.1038/sc.2016.148
3. Amatachaya S, Naewla S, Srisim K, Arrayawichanon P, Siritaratiwat W. Concurrent validity of the 10-meter walk test as compared with the 6-minute walk test in patients with spinal cord injury at various levels of ability. Spinal Cord. 2014 Apr;52(4):333-6. DOI: 10.1038/sc.2013.171
4. Akpinar P, Atici A, Ozkan FU, Aktas I, Kulcu DG, Kurt KN. Reliability of the Spinal Cord Assessment Tool for Spastic Reflexes. Arch Phys Med Rehabil. 2017 Jun;98(6):1113-8. DOI: 10.1016/j.apmr.2016.09.119
5. Arora T, Oates A, Lynd K, Musselman KE. Current state of balance assessment during transferring, sitting, standing and walking activities for the spinal cord injured population: A systematic review. J Spinal Cord Med. 2020 Jan;43(1):10-23. DOI: 10.1080/10790268.2018.1481692
6. Arsh A, Darain H, Ullah I, Shakil-Ur-Rehman S. Diagnostic tests to assess balance in patients with spinal cord injury: a systematic review of their validity and reliability. Asian Biomed (Res Rev News). 2021 Jun 30;15(3):111-8. DOI: 10.2478/abm-2021-0014
7. Arsh A, Darain H, Rahman MU, Ullah I, Shakil-Ur-Rehman S. Reliability of modified functional reach test in the assessment of balance function in people with spinal cord injury: A systematic review. J Pak Med Assoc. 2021 Aug;71(8):2040-4. DOI: 10.47391/JPMA.1276
8. Chan K, Unger J, Lee JW, Johnston G, Constand M, Masani K, Musselman KE. Quantifying balance control after spinal cord injury: Reliability and validity of the mini-BESTest. J Spinal Cord Med. 2019 Oct;42(sup1):141-8. DOI: 10.1080/10790268.2019.1647930
9. Jørgensen V, Opheim A, Halvarsson A, Franzén E, Roaldsen KS. Comparison of the Berg Balance Scale and the Mini-BESTest for Assessing Balance in Ambulatory People With Spinal Cord Injury: Validation Study. Phys Ther. 2017 Jun 1;97(6):677-87. DOI: 10.1093/ptj/pzx030
10. Lemay JF, Nadeau S. Standing balance assessment in ASIA D paraplegic and tetraplegic participants: concurrent validity of the Berg Balance Scale. Spinal Cord. 2010 Mar;48(3):245-50. DOI: 10.1038/sc.2009.119
11. Mills PB, Vakil AP, Phillips C, Kei L, Kwon BK. Intra-rater and inter-rater reliability of the Penn Spasm Frequency Scale in People with chronic traumatic spinal cord injury. Spinal Cord. 2018 Jun;56(6):569-74. DOI: 10.1038/s41393-018-0063-5
12. Poncumhak P, Saengsuwan J, Kamruecha W, Amatachaya S. Reliability and validity of three functional tests in ambulatory patients with spinal cord injury. Spinal Cord. 2013 Mar;51(3):214-7. DOI: 10.1038/sc.2012.126
13. Roy A, Higgins J, Nadeau S. Reliability and minimal detectable change of the mini-BESTest in adults with spinal cord injury in a rehabilitation setting. Physiother Theory Pract. 2021 Jan;37(1):126-34. DOI: 10.1080/09593985.2019.1622161
14. Tomaschek R, Gemperli A, Rupp R, Geng V, Scheel-Sailer A; German-speaking Medical SCI Society (DMGP) Ergebniserhebung Guideline Development Group. A systematic review of outcome measures in initial rehabilitation of individuals with newly acquired spinal cord injury: providing evidence for clinical practice guidelines. Eur J Phys Rehabil Med. 2019 Oct;55(5):605-17. DOI: 10.23736/S1973-9087.19.05676-4

15. van Hedel HJ, Wirz M, Dietz V. Assessing walking ability in subjects with spinal cord injury: validity and reliability of 3 walking tests. Arch Phys Med Rehabil. 2005 Feb;86(2):190-6. DOI: 10.1016/j.apmr.2004.02.010
16. Willi R, Widmer M, Merz N, Bastiaenen CHG, Zörner B, Bolliger M. Validity and reliability of the 2-minute walk test in individuals with spinal cord injury. Spinal Cord. 2023 Jan;61(1):15-21. DOI: 10.1038/s41393-022-00847-1
17. Amatachaya S, Srisim K, Arrayawichanon P, Thaweewannakij T, Amatachaya P. Dual-Task Obstacle Crossing Training Could Immediately Improve Ability to Control a Complex Motor Task and Cognitive Activity in Chronic Ambulatory Individuals With Spinal Cord Injury. Top Spinal Cord Inj Rehabil. 2019 Summer;25(3):260-70. DOI: 10.1310/sci18-00038
18. Adams MM, Hicks AL. Comparison of the effects of body-weight-supported treadmill training and tilt-table standing on spasticity in individuals with chronic spinal cord injury. J Spinal Cord Med. 2011;34(5):488-94. DOI: 10.1179/2045772311Y.0000000028
19. Ben M, Harvey L, Denis S, Glinsky J, Goehl G, Chee S, Herbert RD. Does 12 weeks of regular standing prevent loss of ankle mobility and bone mineral density in people with recent spinal cord injuries? Aust J Physiother. 2005;51(4):251-6. DOI: 10.1016/s0004-9514(05)70006-4
20. Ellapen TJ, Hammill HV, Swanepoel M, Strydom GL. The benefits of hydrotherapy to patients with spinal cord injuries. Afr J Disabil. 2018 May 16;7(0):450. DOI: 10.4102/ajod.v7i0.450
21. Fang CY, Lien AS, Tsai JL, Yang HC, Chan HL, Chen RS, Chang YJ. The Effect and Dose-Response of Functional Electrical Stimulation Cycling Training on Spasticity in Individuals With Spinal Cord Injury: A Systematic Review With Meta-Analysis. Front Physiol. 2021 Nov 19;12:756200. DOI: 10.3389/fphys.2021.756200
22. Gaspar R, Padula N, Freitas TB, de Oliveira JPJ, Torriani-Pasin C. Physical Exercise for Individuals With Spinal Cord Injury: Systematic Review Based on the International Classification of Functioning, Disability, and Health. J Sport Rehabil. 2019 Jul 1;28(5):505-16. DOI: 10.1123/jsr.2017-0185
23. Ibitoye MO, Hamzaid NA, Hayashibe M, Hasnan N, Davis GM. Restoring prolonged standing via functional electrical stimulation after spinal cord injury: A systematic review of control strategies. Biomedical Signal Processing and Control. 2019;49:34-47. DOI: 10.1016/j.bspc.2018.11.006
24. Lawrason SVC, Todd KR, Shaw RB, Martin Ginis KA. Physical activity among individuals with spinal cord injury who ambulate: a systematic scoping review. Spinal Cord. 2020 Jul;58(7):735-45. DOI: 10.1038/s41393-020-0460-4
25. Li C, Khoo S, Adnan A. Effects of aquatic exercise on physical function and fitness among people with spinal cord injury: A systematic review. Medicine (Baltimore). 2017 Mar;96(11):e6328. DOI: 10.1097/MD.0000000000006328
26. Mehrholz J, Harvey LA, Thomas S, Elsner B. Is body-weight-supported treadmill training or robotic-assisted gait training superior to overground gait training and other forms of physiotherapy in people with spinal cord injury? A systematic review. Spinal Cord. 2017 Aug;55(8):722-9. DOI: 10.1038/sc.2017.31
27. Quel de Oliveira C, Refshauge K, Middleton J, de Jong L, Davis GM. Effects of Activity-Based Therapy Interventions on Mobility, Independence, and Quality of Life for People with Spinal Cord Injuries: A Systematic Review and Meta-Analysis. J Neurotrauma. 2017 May 1;34(9):1726-43. DOI: 10.1089/neu.2016.4558
28. Stampacchia G, Gazzotti V, Olivieri M, Andrenelli E, Bonaiuti D, Calabro RS, Carmignano SM, Cassio A, Fundaro C, Companini I, Mazzoli D, Cerulli S, Chisari C, Colombo V, Dalise S, Mazzoleni D, Melegari C, Merlo A, Boldrini P, Mazzoleni S, Posteraro F, Mazzucchelli M, Benanti P, Castelli E, Draicchio F, Falabella V, Galeri S, Gimigliano F, Grigioni M, Mazzon S, Molteni F, Morone G, Petrarca M, Picelli A, Senatore M, Turchetti G, Bizzarrini E. Gait robot-assisted rehabilitation in persons with spinal cord injury: A scoping review. NeuroRehabilitation. 2022;51(4):609-47. DOI: 10.3233/NRE-220061
29. van der Scheer JW, Goosey-Tolfrey VL, Valentino SE, Davis GM, Ho CH. Functional electrical stimulation cycling exercise after spinal cord injury: a systematic review of health and fitness-related outcomes. J Neuroeng Rehabil. 2021 Jun 12;18(1):99. DOI: 10.1186/s12984-021-00882-8
